# Within-family plasticity of nervous system architecture in Syllidae (Annelida, Errantia)

**DOI:** 10.1186/s12983-020-00359-9

**Published:** 2020-06-23

**Authors:** Hannah Schmidbaur, Thomas Schwaha, Rico Franzkoch, Günter Purschke, Gerhard Steiner

**Affiliations:** 1 Department of Integrative Zoology, Faculty of Life Sciences, University of Vienna, Althanstraße 14, 1090 Vienna, Austria; 2 Present address: Department of Molecular Evolution and Development, Faculty of Life Sciences, University of Vienna, Althanstraße 14, 1090 Vienna, Austria; 3 Zoology and Developmental Biology, Department of Biology and Chemistry, University of Osnabrück, Barbarastr. 11, 49069 Osnabrück, Germany; 4 Present address: Microbiology, Department of Biology and Chemistry, University of Osnabrück, Barbarastr. 11, 49069 Osnabrück, Germany

**Keywords:** Nervous system evolution, Neuroanatomy, Syllidae, Phyllodocida, Annelida

## Abstract

**Background:**

The ground pattern underlying the nervous system of the last common ancestor in annelids was long thought to be settled, consisting of a dorsal brain, circumoesophageal connectives and a subepithelial, ladder-like ventral nerve cord with segmental ganglia connected by paired connectives. With the advent of immunocytochemical stainings and confocal laser scanning microscopy, it becomes evident that its architecture is extremely diverse, which makes the reconstruction of a ground pattern in annelida challenging. Whereas the nervous systems of many different families has already been described, only very few studies looked at the diversity of nervous systems within such clades to give a closer estimate on how plastic the annelid nervous system really is. So far, little is known on syllid nervous system architecture, one of the largest and most diverse groups of marine annelids.

**Results:**

The position of the brain, the circumoesophageal connectives, the stomatogastric nervous system, the longitudinal nerves that traverse each segment and the innervation of appendages are relatively uniform within the clade. Both the number of connectives within the ventral nerve cord and the number of segmental nerves, which in earlier studies were used to infer phylogenetic relationships and to reconstruct an annelid ground pattern, are highly diverse and differ between genera or even within a given genus. Differences in the distribution of somata of the brain, the nuchal innervation and its associated cell bodies were found between Syllinae and Exogoninae and may be subfamily-specific.

**Conclusions:**

The nervous system morphology of syllids very likely depends on the taxon-specific ecological requirements. Thus, it is not surprising that in a clade, which occupies such diverse niches as the Annelida, we find similar patterns in phylogenetically widely separated species in similar niches and a high degree of modularity within a family. Only standardized protocols and staining methods can lead to comparable results, but so far different approaches have been taken to describe annelid nervous systems, making homologization of certain structures difficult. This study provides the first thorough description of the nervous system in the family Syllidae, allowing more detailed comparisons between annelid families in the future.

## Background

An increasing amount of studies on the nervous system of various invertebrates are being published as methods such as immunocytochemical stainings and confocal laser scanning microscopy advance [1–24]. To date, however, there is very little information on nervous system plasticity and diversity at different taxonomic levels. Often, only one or a few species have been described and are regarded as representatives for the entire family.

The nervous system of annelids was generally described to consist of a rope-ladder like ventral nerve cord with ventral connectives joining segmentally arranged ganglia. The actual situation is more complex [25–27] and the underlying ground pattern is being discussed to date [27], with authors suggesting two [7, 28] or five [26] connectives present in the ventral nerve cord of the last common ancestor of annelids.

The incorporation of data on the nervous system of 14 annelid families within a phylogenomic framework led to equivocal results regarding the ventral nerve cord and its commissures in the last common ancestor of annelids [29]. The high diversity of nervous system architecures in recent annelid clades, including the likely reduction of features in early branching families such as the Oweniidae [30] and Sipunculidae [31], makes it difficult to reconstruct the ground pattern at present.

The ventral nerve cord and the brain are connected by a dorsal and a ventral root of the circumoesophageal connectives, which may fuse to different degrees [26]. These connectives give rise to the nerves innervating the tentacular cirri [32], if present. Each root of the circumoesophageal connectives forms two commissures within the brain [25, 26, 32]. Roots emanating from the dorsal and ventral commissures of the dorsal and ventral root of the circumoesophageal connectives innervate the anterior appendages called palps [25]. The antennae are innervated from the dorsal commissure alone. Several clusters of somata can be found in the anterior region such as posterior ganglia, palp ganglia and ganglia of the cirumoesophageal connective [25, 32]. In many annelid species structures resembling arthropod mushroom bodies have been described [33], but these most likely have evolved independently in Annelida [34–36].

The pharynx is, especially in annelids with a muscular axial protrusible proboscis, highly complex and little is known on its innervation patterns [27]. It is innervated by the stomatogastric nervous system arising from the brain and the circumoesophageal connectives [27]. The peripheral innervation of body segments was first described in three species of Nereididae [37]. It consists of several segmental nerves which can form dorsal commissures [25, 27]. Usually, the second one is the largest and innervates the parapodium [37], but the nomenclature can change depending on the number of segmental nerves. It has been reported that the number of segmental nerves can vary among species of the same family [2, 26, 27] and their homologisation is at present difficult [11, 25, 27]. In parapodia-bearing annelids, the parapodial nerve is considered to be homologous [2]. Reaching the parapodial lobe it splits into two roots, one innervating the dorsal and one the ventral cirrus [2, 38]. These cirri are important sensory organs comprising numerous receptor cells which send their processes into the ventral cord.

In addition to the ventral nerve cord, several longitudinal nerves running ventrally, laterally and dorsally have been described for many species [27]. They seem to be part of the ground pattern in annelids, but at present only the unpaired dorsal longitudinal nerve can be homologised across annelids [14].

Even though the significance of evolutionary changes in central nervous systems is still discussed, it is often regarded as conserved within phyla [38–40]. However, it has long been observed that the annelid nervous system varies considerably across the phylum, thus reflecting the broad ecological and morphological diversity of the phylum which relativates the statement above [27, 29]. Detailed morphological analysis can yield valuable information on nervous system evolution and add information on phylogenetic relationships, even if the involved taxa differ in other morphological aspects [41, 42]. While the development of the nervous system in annelids becomes focus of more studies, and comparisons between families help to disentangle the phylogenetic tree [29], little is known on generic variation. It has been shown in Naididae, the biggest clitellate family with about one thousand species [43], that much of the variation of neuronal features in the family was overlooked before a detailed study of the clade [20].

**Fig. 1:**
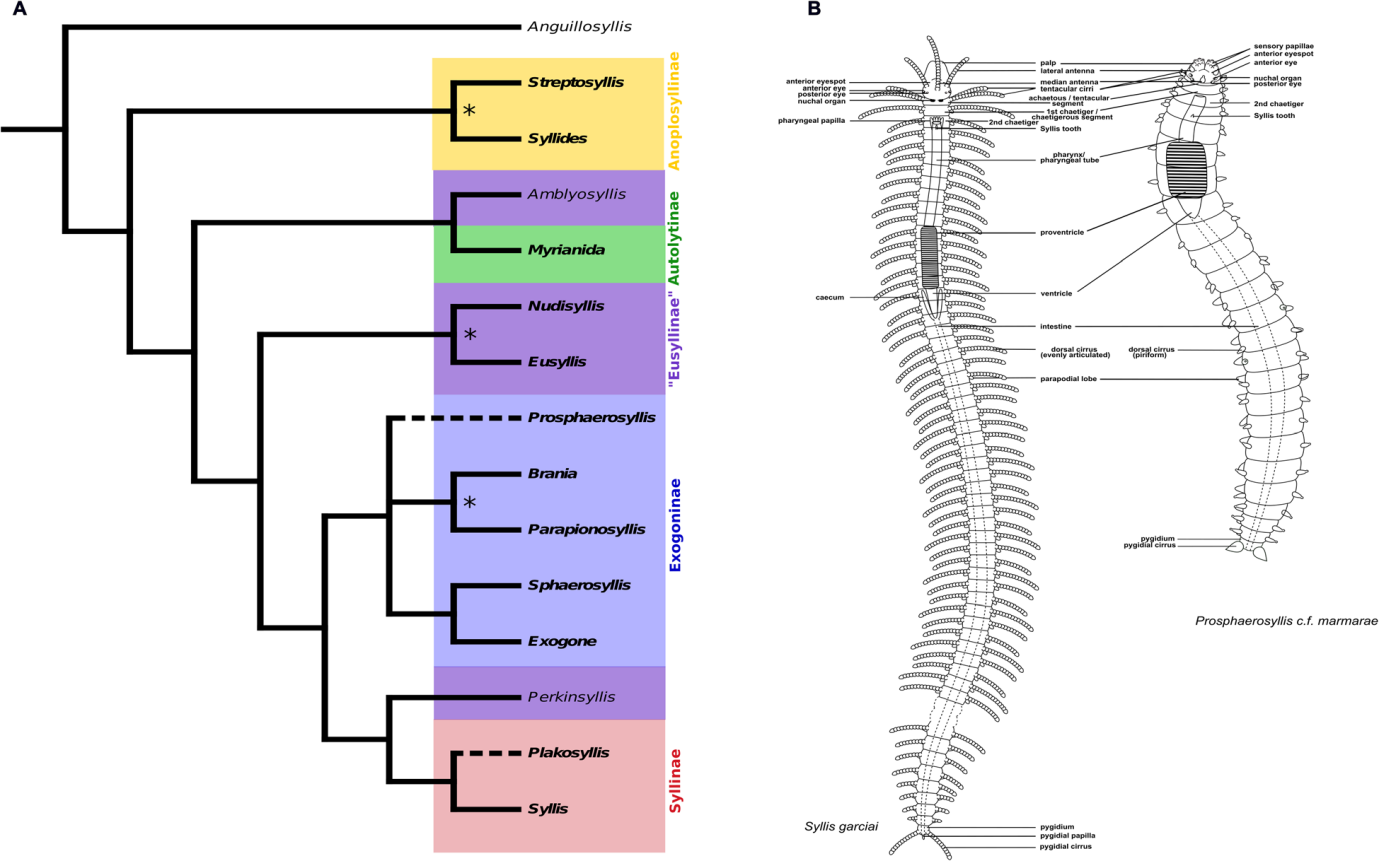
Phylogenetic relationships and general morphology of Syllidae. **a**: Summary of the phylogenetic relationships of Syllidae after [46]. Only genera used in this study (bold) are depicted, except for *Anguillosyllis*, *Amblyosyllis* and *Perkinsyllis*, which indicate some of the unresolved relationships in Syllidae. An asterisk indicates that the relationship between these genera is not resolved or at least one of the genera is polyphyletic. Dashed lines indicate that these genera were not included in the phylogenetic analysis by [46]. Yellow - Anoplosyllinae, purple - paraphyletic Eusyllinae, green - Autolytinae, light blue - Exogoninae, red - Syllinae. **b**: Schemata representing the general morphology of Syllidae exemplified with *Syllis tyrrhena* (Syllinae) and *Prosphaerosyllis marmarae* (Exogoninae). Prostomium bears palps, 3 antennae, 2–6 eyes and nuchal organs; achaetous segment without parapodia but with one or two pairs of tentacular cirri; following segments bear parapodia with dorsal and ventral cirri (Autolytinae lack ventral cirri). Digestive tract consists of an eversible pharyngeal tube, the muscular proventricle, ventricle and intestine. The pygidium bears a pair of pygidial cirri and sometimes a median pygidial papilla

Syllidae inhabit a wide variety of habitats and comprises more than 700 species in 74 genera [44–47]. Currently five subfamilies are recognized, but phylogenetic analyses have shown that only four are monophyletic and a number of genera are currently not assigned to any subfamily [45, 46] (Fig. 1a). So far little is known on how their morphological and ecological diversity affects internal anatomy, nervous system and sensory organs of different species within this large clade. Here, we present an extensive study of the nervous system of species within the family Syllidae, with an overview on 21 species from 12 genera of all subfamilies of Syllidae (Suppl. Fig. S1, Table 1). Three species of Syllinae (*Plakosyllis brevipes*, *Syllis garciai and Syllis* cf. *tyrrhena*) and two species of Exogoninae (*Prosphaerosyllis marmarae* and *Sphaerosyllis taylori*) were investigated in more detail. As staining intensity can differ between specimens, and several scans of different individuals are needed to account for this variation, only these five species, which were readily available and easy to collect, were used to describe differences in the microanatomy of the brain, anterior clusters of somata and segmental innervation.

**Table 1:** Species included in this study, sampling location and habitat

Subfamily	Species	Location	Sampled habitat
Anoplosyllinae	*Syllides longocirratus* *Streptosyllis sp. (juveniles)* *Streptosyllis websteri*	Roscoff (unpubl. data from previous study) Rovinj, Croatia (Adriatic Sea) Sylt, Germany (North Sea)	Sublittoral Sand Sand Eulittoral Sand
Autolytinae	*Myrianida prolifera* *Myrianida sp.*	Helgoland (unpubl. data from previous study) Bergen, Norway (North Sea)	Plankton and between algae and hydrozoans On hydrozoan colonies
“Eusyllinae”	*Eusyllis blomstrandi* *Nudisyllis c.f. pulligera*	Roscoff (unpubl. data from previous study) Roscoff (unpubl. data from previous study)	Between algae Between algae
Exogoninae	*Brania clavata* *Brania pulsilla* *Exogone naidina* *Parapionosyllis labronica* *Parapionosyllis minuta* *Sphaerosyllis hystrix* *Sphaerosyllis tetralix* *Sphaerosyllis taylori* *Prosphaerosyllis marmarae*	Kristineberg (unpubl. data from previous study) Luc sur Mer (unpubl. data from previous study) Sylt, Germany (North Sea) Crete (unpubl. data from previous study) Roscoff (unpubl. data from previous study) Helgoland (unpubl. Data from previous study) Roscoff (unpubl. data from previous study) Beirut, Lebanon (Levantine Sea) Rovinj, Croatia (Adriatic Sea)	Sublittoral sediment Sublittoral sand Eulitoral sediment Sublittoral sediments Sublittoral sand Sublittoral sand Sublittoral sand Sublittoral sand Sublittoral sand
Syllinae	*Syllis krohnii* *Syllis sp.* *Syllis tyrrhena* *Syllis garciai* *Plakosyllis brevipes*	Roscoff (unpubl. data from previous study) Rosocff (unpubl. data from previous study) Rovinj, Croatia (Adriatic Sea) Rovinj, Croatia (Adriatic Sea) Rovinj, Croatia (Adriatic Sea)	Sublittoral sand Sublittoral sand Sublittoral sand Sublittoral sand Sublittoral sand

## Results

### Overview of the nervous system in Syllidae

According to Fauchald and Rouse and Pleijel [48, 49] the anterior end of syllids comprise a well-developed prostomium, a peristomium reduced to the lips and an achaetigerous first segment followed by the second, chaetigerous segment (Fig. 1b). Their terminology is used in this study. The achaetous segment bears one or two pairs of tentacular cirri.

All species have a dorsally positioned brain, which lies within the prostomium and is surrounded by somata. In some genera of Exogoninae (*Prosphaerosyllis, Sphaerosyllis*) it extents into the first segments. The brain is connected by the dorsal (drcc) and ventral root (vrcc) of the circumoesophaegeal connectives to the ventral nerve cord. It is is associated with the following sensory organs: a pair of palps (which may be fused to various extends), a pair of lateral antennae and an unpaired median antenna, usually two pairs of cerebral eyes which may be accompanied by a pair of minute anterior eye spots, a pair of laterofrontal sense organs, and the nuchal organs. In certain species part of the antennae and eyes or laterofrontal sense organs may be absent.

In Syllidae the ventral nerve cord always consists of a pair of main ventral nerves and an unpaired median nerve (suppl Fig. S1). The only exception is *Plakosyllis brevipes*, which has a pair of main ventral nerves, a pair of paramedian ventral nerves and an unpaired median nerve (see section ventral nerve cord). The ventral nerves can fuse to different degrees at the segmental boundaries, but are usually discernible in the region of the ventral ganglia. No clear pattern specific for either one subfamily in its segmental innervation could be discerned. All species have at least three segmental nerves (suppl. Fig. S1). A forth, intersegmental nerve is present in most species, but can be distributed irregularly and was not observed in the Anoplosyllinae, *Eusyllis* (“Eusyllinae”), *Brania pulsilla, Exogone naidina* (staining generally very weak)*, Parapionosyllis labronica, Parapionosyllis minuta* (Exongoninae) (suppl. Fig. S1). The parapodial or second segmental nerve is split into at least three neurite bundles.

### Microanatomy of the brain and innervation of anterior sensory appendages

The following sections of the manuscript deal only with the five species *Plakosyllis brevipes*, *Syllis garciai and Syllis* cf. *tyrrhena* (Syllinae), and *Prosphaerosyllis marmarae* and *Sphaerosyllis taylori* (Exogoninae) unless otherwise mentioned.

**Fig. 2:**
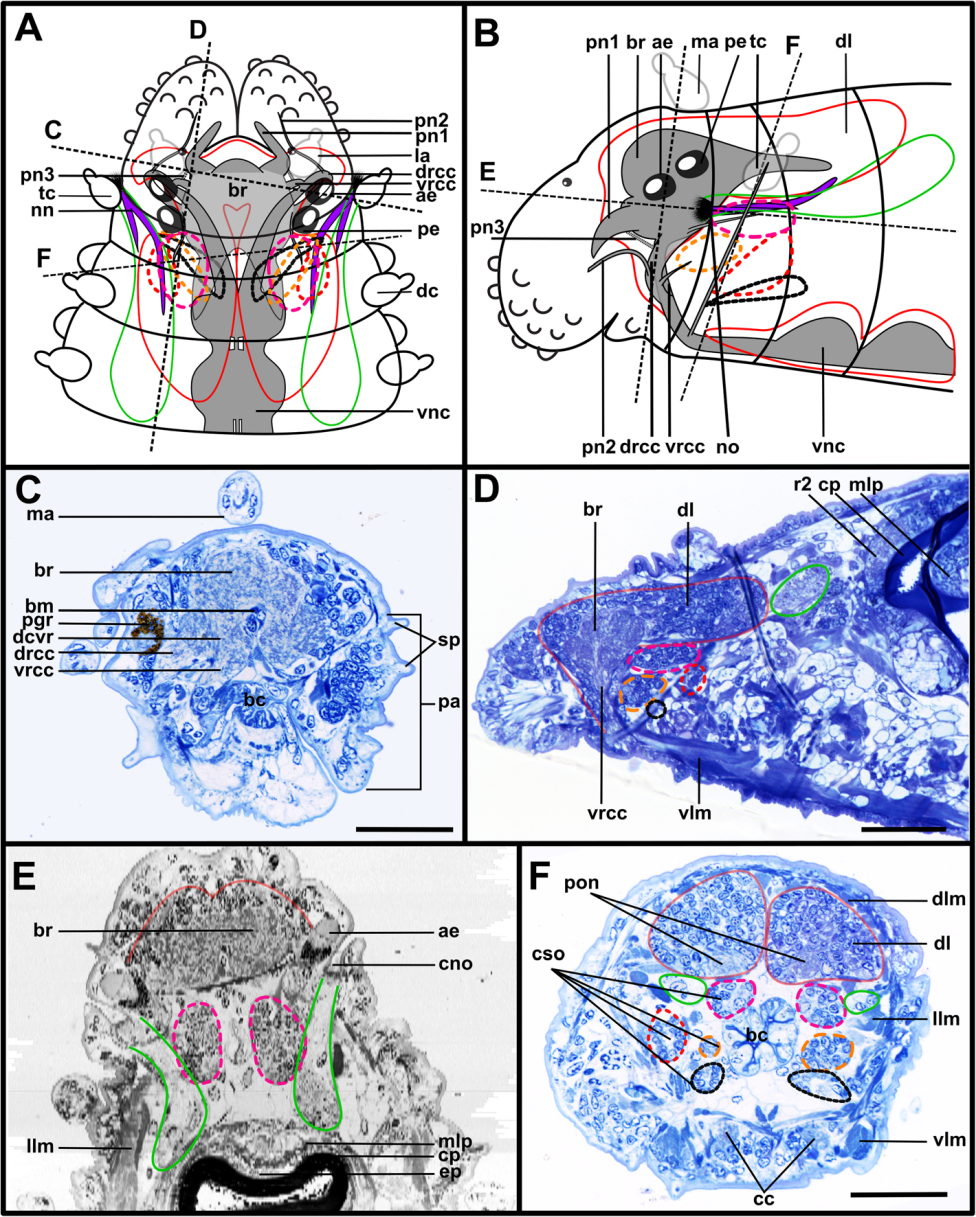
*Prosphaerosyllis marmarae*. Brain and associated clusters of somata. **a, b**: Schemata of prostomium, achaetous segment and first two chaetigers. Dotted lines indicate position of sections C, D, E and F. Neuropil of the brain laterally and dorsally enveloped by somata (red outline). Somata form a pair of posterior lobes and four additional clusters (dashed outline). Support cell nuclei of nuchal organs form a pair of lateral lobes (green outline). Neurite bundles (purple) reach from the nuchal organ into the dorsal lobes, a few neurite bundles reach into the lateral lobes. Posteroventral cluster of somata (dotted pink line = posterior inferior cluster) possibly consisting of somata of the receptor cells of the nuchal organ receiving fibres from the nuchal organ and from the drcc. A cluster of somata (dotted orange line) lies ventrally to the posterior inferior clusterand receives fibres from the drcc and two clusters are associated to the tentacular cirrus (dotted red and black lines). **c**: Slightly oblique cross section of the. **d**: left side of the brain, showing the left dorsal lobe, the brain, the ventral root of the circumoesophageal connective, the nuchal lobes and other clusters of somata. **e**: Dorsal frontal section of the same individual as C and F. The nuchal organs lie laterally. Cilia of support cells penetrate the cuticle and are in contact with the environment. Support cells reach into the first and second chaetigerous segment (nuchal lobes). **f**: Cross section through dorsal lobes and all five clusters of somata. Posterior neurite bundles of the brain reach far into the dorsal dorsal lobes. **Scale bars = 50** μm**. Abbreviations**: ae – anterior eye; bc – buccal cavity; bm – muscle penetrating brain; br – brain; cc – circumoesophageal connective; cno – cilia of support cells of nuchal organ; cp – cuticularised layer of pharynx; cso – clusters of somata; dc – dorsal cirrus; dcvr – dorsal commissure of ventral root of circumoesophageal connective; dl – dorsal lobe of the brain; dlm – dorsal longitudinal muscle; drcc – dorsal root of circumoesophageal connective; ep – epidermal layer of pharynx; la – lateral antenna; llm – lateral longitudinal muscle; ma – median antenna; mlp – muscular layer of pharynx; nn - nuchal nerve; no – nuchal organ; pa – palp; pe – posterior eye; pgr – pigment granules of eye; pn 1–3 – palp nerves 1–3; pon – posterior neurite bundle of the brain; r2 – second stomatogastic ring neurite bundle; sp. – sensory papillae; tc – tentacular cirrus; vlm – ventral longitudinal muscle; vnc – ventral nerve cord; vrcc – ventral root of circumoesophageal connective

**Fig. 3:**
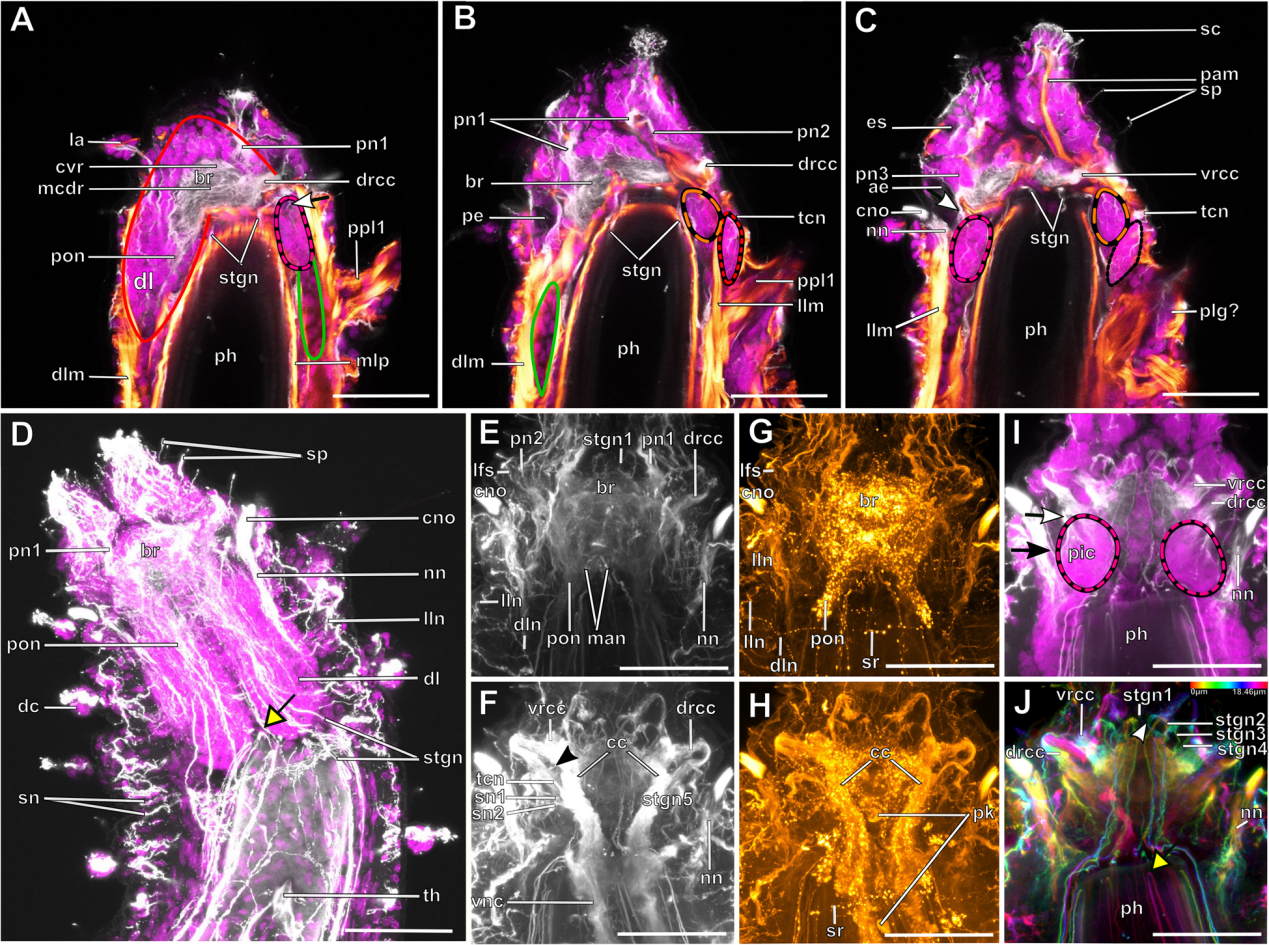
*Prosphaerosyllis marmarae.* Innervation of prostomium and anterior segments **a-c**: Single optical frontal sections of the same individual. Anterior end and first two chaetigers, slightly oblique. α-tubulin-lir (grey), cell nuclei (magenta) and f-actin (orange glow) staining combined. **a**: Dorsal; white arrow marks neurite bundle reaching from drcc into posteroventral (possible nuchal) cluster of somata (pink dotted line). Cell nuclei within nuchal lobes (green line) are hard to recognise. The somata of the brain (red line) form a pair of dorsal lobes. **b**: Median portion of the brain. Fibres from the tentacular cirrus neurite bundle enter the first of the tentacular clusters of somata (red dotted line) **c**: Ventral section of the brain. Arrow indicates neurite bundles reaching into the posterior inferior cluster of somata (pink dotted line), next to the nuchal neurite bundle. Second tentacular cluster of somata (black dotted line) receives fibres from the tentacular cirri neurite bundle. **d**: Dorsal maximum intensity z-projection of anterior end and first three chaetigers, α-tubulin-lir (white) and cell nuclei (magenta). Yellow arrow indicates loop where stomatogastric neurite bundles enter pharyngeal epithelium. **e-j**: Maximum intensity z-projections of different sections of one individual. **e**: Dorsal part of the brain (α-tubulin-lir). **f:** Ventral part of the brain. Black arrowhead indicates the neurite bundle giving rise to ventral, lateral and dorsal longitudinal neurite bundles. **g**: Serotonin-lir of the brain, dorsal. A third stomatogastric ring neurite bundle is visible. **h:** Serotonin-lir of the brain, ventral. **i:** Median planes of α-tubulin-lir and cell nuclei. Posterior inferior clusters of somata (dotted pink lines) receive fibres from drcc (white arrow) and nuchal neurite bundle (black arrow). **j**: Colour-coded z-projection of ventro-median planes of the same individual as in E-I to visualise individual stomatogastric neurite bundles. A strong stomatogastric neurite bundle extends towards the lips (white arrowhead). **Scale bars = 50** μm**. Abbreviations**: ae – anterior eye; br – brain; cc – circumoesophageal connective; cno – cilia of support cells of nuchal organ; cvr – commissure of vrcc; dc – dorsal cirrus; dl – dorsal lobe of the brain; dlm – dorsal longitudinal muscle; dln – dorsal longitudinal neurite bundle; drcc – dorsal root of circumoesophageal connective; es – eyespot; la – lateral antenna; lfs – laterofrontal sense organ; llm – lateral longitudinal muscle; lln – lateral longitudinal neurite bundle; man – neurite bundles innervating median antenna; mlp – muscular layer of pharynx; nn – nuchal neurite bundle; pa – palp; pam – palp muscle; pe – posterior eye; ph – pharynx; pk – serotonin-lir perikarya; plg – parapodial ganglion/cluster of somata; pn1 – main palp neurite bundle; pn2 – palp neurite bundle originating from drcc; pon – posterior neurite bundle of the brain; ppl1 – parapodial lobe of first chaetiger; sc – sensory cells; sn – segmental neurite bundle; sn1 and 2 – segmental neurite bundles of tentacular segment; sp. – sensory papillae; sr – serotonin-lir stomatogastric ring neurite bundle; stgn 1–5 – stomatogastric neurite bundles 1–5; tcn – neurite bundle innervating tentacular cirri; th – tooth; vlm – ventra longitudinal muscle; vnc – ventral nerve cord; vrcc – ventral root of circumoesophageal connective

**Fig. 4:**
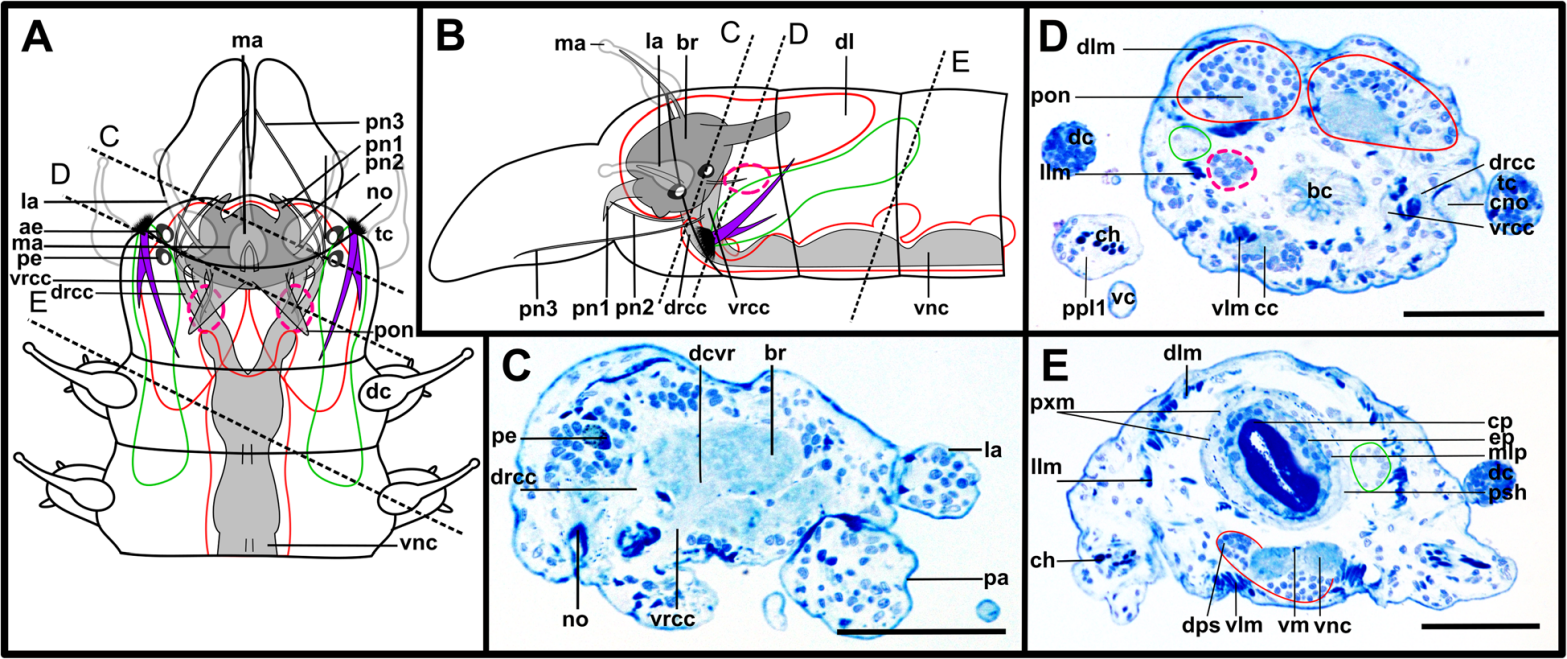
*Sphaerosyllis taylori*. Brain and associated clusters of somata. **a, b**: Schemata of the prostomium, achaetous segment and first two chaetigers. Dotted lines indicate position of sections C, D and E. Segmental neurite bundles, stomatogastric neurite bundles, laterofrontal sense organs etc. omitted for better overview. The neuropil of the brain is enveloped by somata (red). The somata form a pair of posterior lobes and one posterior inferior cluster of somata (pink dotted line) is present. A pair of lateral nuchal lobes (green line) reaches posteriorly from the nuchal organs. Neurite bundles (purple) of the nuchal organ reach both into the nuchal lobes and the dorsal lobes. **c**: Cross section through the brain showing the neuropil and circuloesophageal connectives. **d**: Dorsal lobes, nuchal lobes and posterior inferior cluster of somata. **e:** Second chaetiger and somata of the ganglion of the ventral nerve cord extend dorsally (red), forming dorsoposterior clusters. **Scale bars = 50** μm. **Abbreviations**: ae – anterior eye; bc – buccal cavity; br – brain; cc – circumoesophageal connective; ch – chaeta; cno – cilia of support cells of nuchal organ; cp – cuticularised layer of pharynx; dc – dorsal cirrus; dcvr – dorsal commissure of vrcc; dl – dorsal lobe; dlm – dorsal longitudinal muscle; dps – dorsoposterior cluster of somata of the ventral nerve cord only present in *Sph. taylori*; drcc – dorsal root of circumoesophageal connective; ep – epidermal layer of pharynx; la – lateral antenna; llm – lateral longitudinal muscle; ma – median antenna; mlp – muscular layer of pharynx; no – nuchal organ; pa – palp; pe – posterior eye; pn 1–3 – palp nerves 1–3; pon – posterior neurite bundle of the brain; ppl1 – parapodial lobe of first chaetiger; psh – pharyngeal sheath; pxm – pharynx muscle; tc – tentacular cirrus; vc – ventral cirrus; vlm – ventral longitudinal muscle; vm – ventromedian longitudinal muscle above vnc; vnc – ventral nerve cord; vrcc – ventral root of circumoesophageal connective

**Fig. 5:**
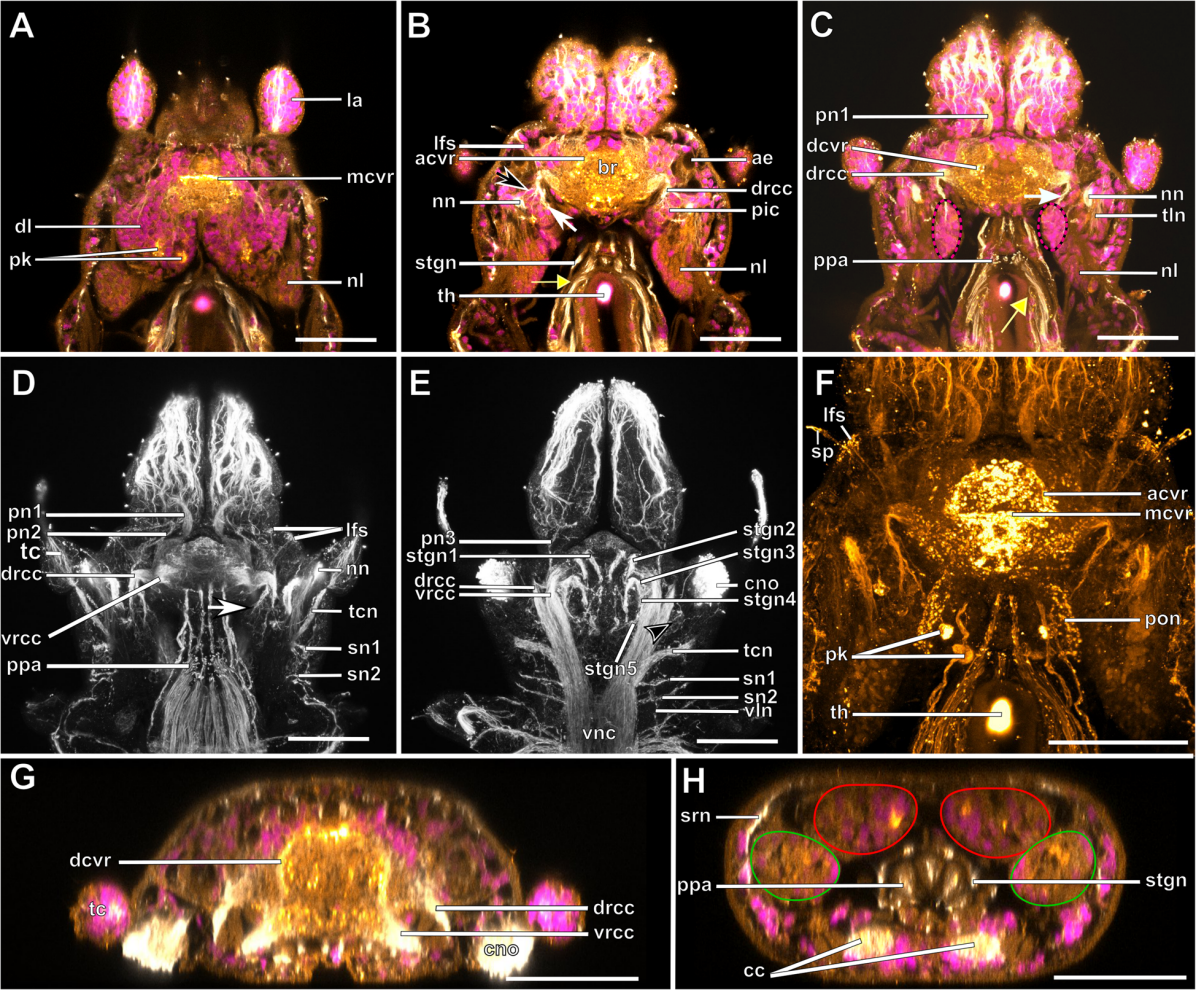
*Sphaerosyllis taylori*. Innervation of prostomium and anterior segments. **a-c**: Single optical frontal sections of α-tubulin-lir (grey), serotonin-lir (and autofluorescence of tissue) (orange) and cell nuclei (magenta) of a single individual. **a**: Dorsal and nuchal lobes. **b**: Neuropil of the brain. Fibres (black arrow) of the nuchal neurite bundle enter the posterior inferior cluster of somata as do fibres (white arrow) of the dorsal root of the circumoesophageal connective. Dorsal fibres of the vrcc form an anterior commissure. The yellow arrow indicates the loop where the stomatogastric neurite bundles enter the pharyngeal epithelium. **c**: Ventral part of the brain showing posterior inferior cluster of somata (pink dotted line). **d**: Maximum intensity z-projection of dorsal part of brain showing where the circumoesophageal connectives enter the brain. **e**: Maximum intensity z-projection of ventral part of brain. The black arrowhead indicates the neurite bundle forming ventral, lateral and dorsal longitudinal neurite bundles. **f**: Maximum intensity z-projection of serotonin-lir of dorsal planes. **g**: Resliced cross section through the brain, combined α-tubulin-lir, serotonin-lir and cell nuclei staining. The dcvr reaches dorsally, forming a median (and an anterior) dorsal commissure. **h**: Resliced cross section showing the position of the dorsal and nuchal lobes, combined α-tubulin-lir, serotonin-lir and cell nuclei staining. **Scale bars = 50** μm. **Abbreviations**: acvr – anterior dorsal commissure of vrcc; ae – anterior eye; br – brain; cc – circumoesophageal connective; cno – cilia of support cells of nuchal organ; dcvr – dorsal commissure of vrcc; dl – dorsal lobe; drcc – dorsal root of circumoesophageal connective; la – lateral antenna; lfs – laterfrontal sense organ; mcvr – median dorsal commissure of vrcc; nl – nuchal lobe; nn – nuchal neurite bundle; pic – posterior inferior cluster of somata; pk – serotonin-lir perikarya; pn 1 – main palp neurite bundle; pn 2 – palp neurite bundle from drcc; pn 3 – palp neurite bundle from vrcc; pon – posterior neurite bundle of the brain; ppa – pharyngeal papilla; sn 1 and 2 – segmental neurite bundles innervating posterior edge of tentacular segment; sp. – sensory papilla; srn – segmental ring neurite bundle; stgn 1–5 – stomatogastric neurite bundles 1–5; tc – tentacular cirrus; tcn – neurite bundle innervating tentacular cirrus; tln – tubulin-lir fibres from nuchal organ entering nuchal lobe; th – tooth; vln – ventral longitudinal neurite bundle

**Fig. 6:**
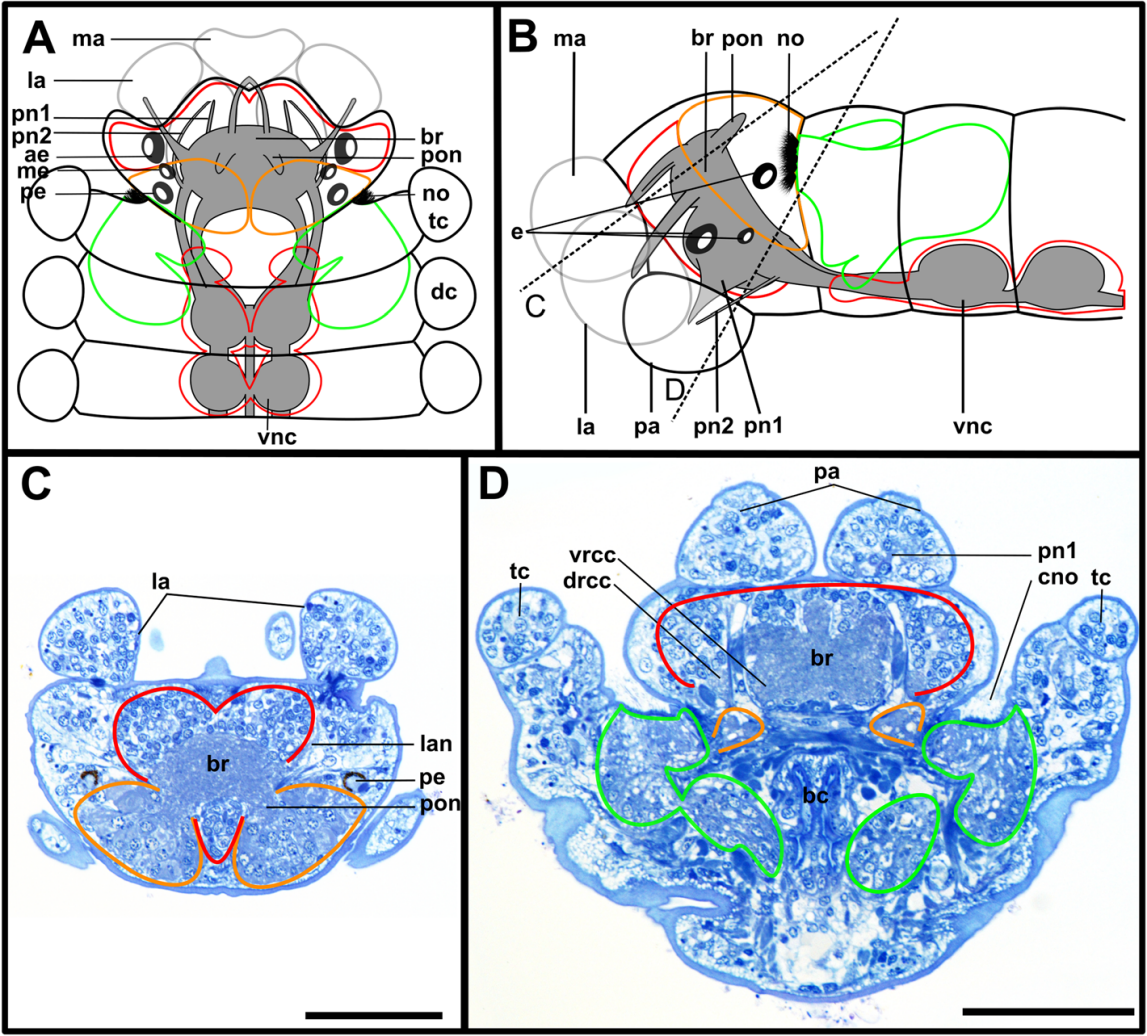
*Plakosyllis brevipes.* Brain and associated clusters of somata. **a and b**: Schemata of the anterior end of *Plakosyllis brevipes*. Dotted lines indicate the position of the sections C, and D. The neuropil of the brain is enveloped by somata (red). Between somata of the brain and nuchal organ lie the somata of the primary sense organs of the nuchal organ (orange). The nuchal lobes (green) reach into the first chaetiger. **c**: Section through the brain. Posterior neurite bundles of the brain reach into clusters of the primary sensory cells of the nuchal organ. **d**: Section through the brain, achaetous segment and first chaetiger, showing the nuchal lobes. **Scale bars = 50** μm. **Abbreviations**: ae – anterior eye; bc – buccal cavity; br – brain; cno – cilia of support cells of nuchal organ; dc – dorsal cirrus; drcc – dorsal root of circumoesophageal connective; e – eye; la – lateral antenna; lan – neurite bundle innervating lateral antenna; ma – median antenna; me – median eye; no – nuchal organ; pa – palp; pe – posterior eye; pn1, 2 – palp neurite bundle 1, 2; pon – posterior neurite bundle; tc – tentacular cirrus; vnc – ventral nerve cord; vrcc – ventral root of circumoesophageal connective

**Fig. 7:**
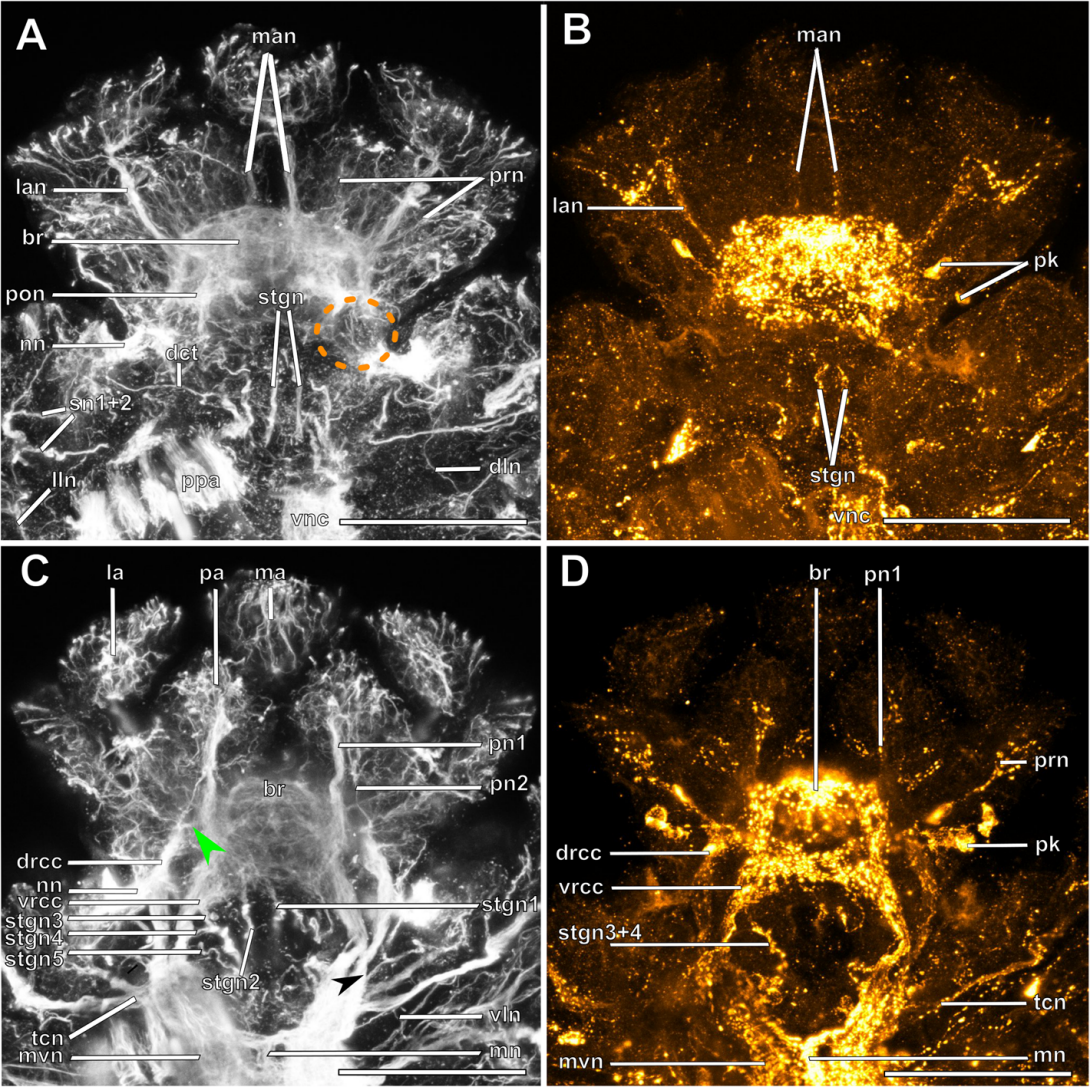
*Plakosyllis brevipes.* Innervation of prostomium and anterior segments. **a**: Maximum intensity z-projection of α-tubulin-lir, dorsal sections of the brain and tentacular segment. The nuchal organ sends fibres towards the brain, which connect to the posterior neurite bundles of the brain (orange dotted circle). **b**: Maximum intensity z-projection of serotonin-lir, dorsal sections of the brain. **c**: Maximum intensity z-projection of α-tubulin-lir, ventral sections of the brain and tentacular segment. The main palp neurite bundle mainly receives bundles from the ventral root of the circumoesophageal connective which are joined by a few fibres from the dorsal root of the circumoesophageal connective (green arrowhead). **d**: Maximum intensity z-projection serotonin-lir of ventral planes of the brain. **Scale bars = 50** μm. **Abbreviations**: br – brain; dct – dorsal commissure of tentacular segment; dln – dorsal longitudinal neurite bundle; drcc – dorsal root of circumoesophageal connective; la – lateral antenna; lan – neurite bundle innervating lateral antenna; lln – lateral longitudinal neurite bundle; ma – median antenna; man – neurite bundle innervating median antenna; mn – median ventral nerve; mvn – main ventral nerve; nn – nuchal neurite bundle; pa – palp; pk – serotonin-lir perikarya; pn1 – main palp neurite bundle; pn2 – palp neurite bundle from drcc; pon – posterior neurite bundle; ppa – pharyngeal papilla; prn – neurite bundles innervating prostomium; sn 1 + 2 – neurite bundles innervating posterior edge of tentacular segment; stgn 1–5 – stomatogastric neurite bundle; tcn – neurite bundle innervating tentacular cirri; vln – ventral longitudinal neurite bundle; vnc – ventral nerve cord; vrcc – ventral root of circumoesophageal connective

**Fig. 8:**
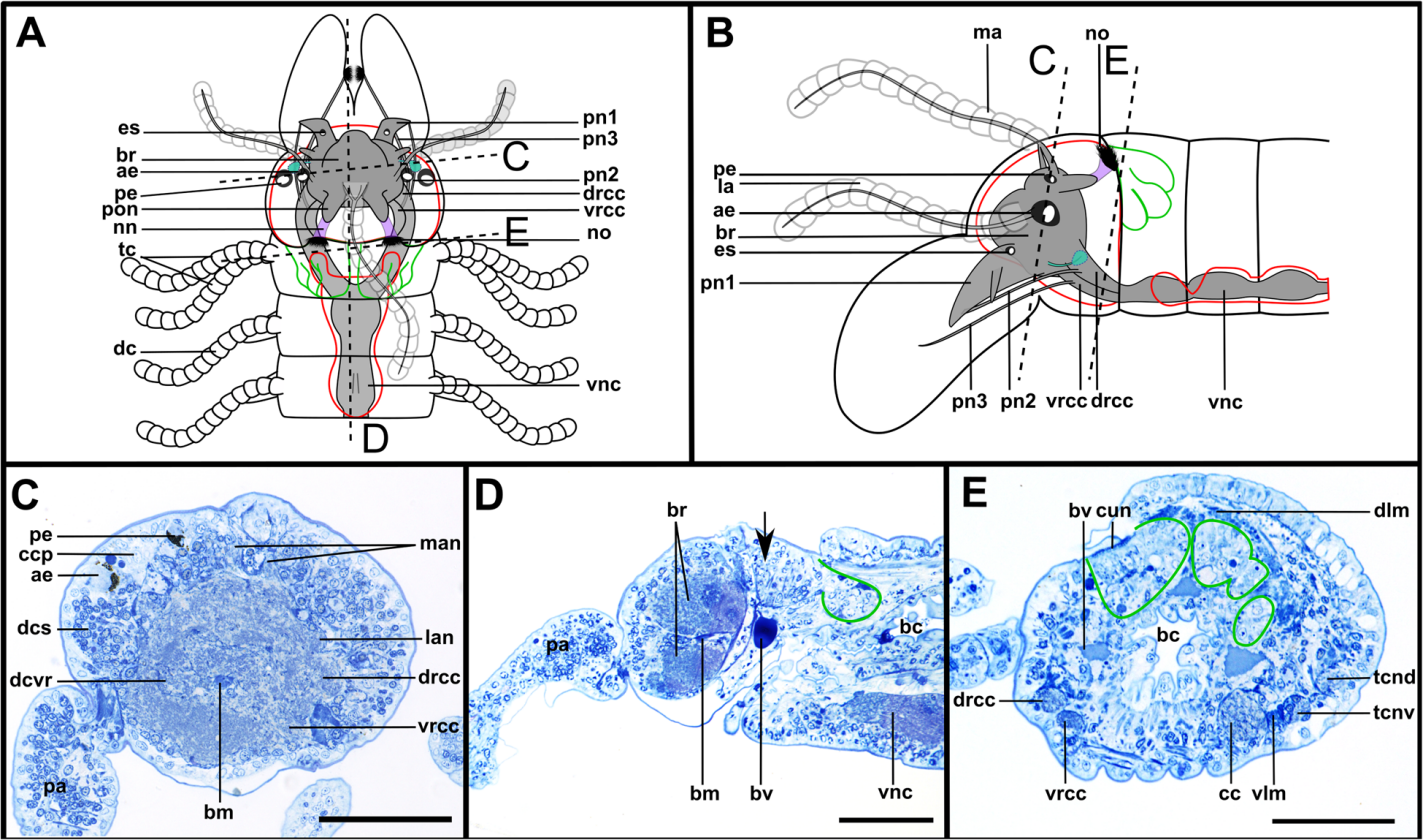
*Syllis tyrrhena.* Brain and associated clusters of somata. **a, b**: Schemata of the anterior end*.* Dotted lines indicate the position of the sections C, D and E. The neuropil of the brain is enveloped by somata (red). The nuchal lobes (green) are restricted to the achaetous segment. A pair of dorsal clusters of somata (turquoise) lie laterofrontally to the brain. **c:** Semi-thin cross section of the brain, slightly shifted. Drcc and vrcc enter the neuropil of the brain **d**: Semi-thin saggital section through the brain and nuchal organ. Somata of primary sensory cells (black arrow) and somata of the brain are hardly distinguishable. **e:** Oblique semi-thin cross section through the nuchal organs. Clusters of somata of support cells reach posteriorly and form a pair of nuchal lobes. **Scale bars = 50 μm**. **Abbreviations**: ae – anterior eye; bc – buccal cavity; bm – muscle penetrating the brain; br – brain; bv – blood vessel; cc – circumoesophageal connective; ccp – cells of crescent shaped ciliary patch; cun – cuticle of nuchal organ; dcs – dorsal cluster of somata; dcvr – dorsal commissure of vrcc; dlm – dorsal longitudinal muscles; drcc – dorsal root of circumoesophageal connective; es – eye spot; la -lateral antenna; lan – neurite bundle innervating lateral antenna; ma – median antenna; man – neurite bundle innervating median antenna; nn – nuchal neurite bundle; no – nuchal organ; pa – palp; pn1–3 – palp nerves 1–3; pe – posterior eyes; pon – posterior neurite bundle of the brain; tcnd – neurite bundle innervating dorsal tentacular cirrus; tcnv – neurite bundle innervating ventral tentacular cirrus; vlm – ventral longitudinal muscle; vnc – ventral nerve cord; vrcc – ventral root of circumoesophageal connective

**Fig. 9:**
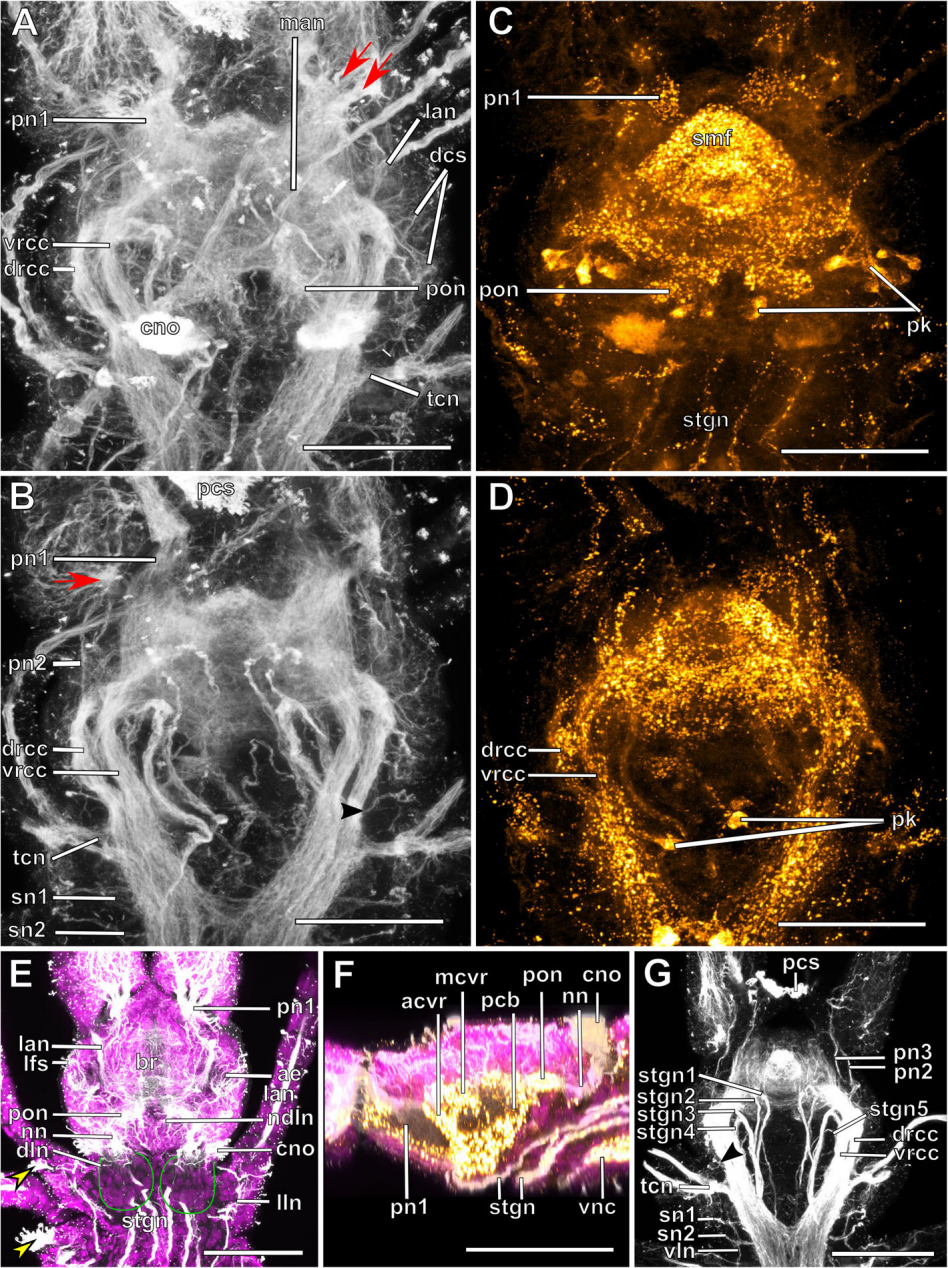
*Syllis tyrrhena.* Innervation of prostomium and anterior segments. **a**: Maximum intensity projection of dorsal sections, α-tubulin-lir. Several fibre bundles (red arrow) separate from the main palp neurite bundle and enter the palps. The neuropil of the brain appears relatively uniform, individual commissures are not visible. **b**: Maximum intensity projection, ventral, α-tubulin-lir. The black arrowhead indicates the neurite bundle forming ventral, dorsal and lateral longitudinal neurite bundles. Red arrows indicate neurite bundles which separate from the main palp neurite bundle. **c**: Maximum intensity z-projections, dorsal, serotonin-lir. Several perikaryal are visible behind the brain. **d**: Maximum intensity z-projections, ventral, serotonin-lir. **e**: Maximum intensity z-projection of α-tubulin-lir (grey) and cell nuclei (magenta), dorsal. Yellow arrowheads indicate dorsolateral ciliary patches on anterior segments. **f**: Maximum intensity z-projection, resliced saggital sections, combined α-tubulin-lir (grey), serotonin-lir (orange) and cell nuclei (magenta). The posterior nerves connect directly to the primary sensory neurites of the nuchal organ **g**: Maximum intensity z-projection of ventral planes of α-tubulin-lir. A black arrowhead indicates the neurite bundle forming dorsal, lateral and ventral longitudinal neurite bundles. **Scale bars = 50 μm**. **Abbreviations**: acvr – anterior dorsal commissure of vrcc; ae – anterior eye; br – brain; cno – cilia of support cells of nuchal organ; dcs – dorsal cluster of somata; dln – dorsal longitudinal neurite bundle; drcc – dorsal root of circumoesophageal connective; lan – neurite bundle innervating lateral antenna; lfs – laterofrontal sense organ; lln – lateral longitudinal neurite bundle; man – neurite bundle innervating median antenna; mcvr – median dorsal commissure of vrcc; ndln – fibres from nuchal organ joining dorsal longitudinal neurite bundle; nn – nuchal neurite bundle; pa – palp; pcb – posterior dorsal commissure of the brain receiving fibres from drcc; pcs – ciliary patches on palps; pk – serotonin-lir perikarya; pn1 – main palp neurite bundle; pn2 – palp neurite bundle from drcc; pn3 – palp neurite bundle from vrcc; pon – posterior neurite bundle; smf – spherical median front of the brain; sn1, sn2 – segmental neurite bundles of tentacular segment; stgn1–5 – stomatogastric neurite bundles 1–5; tcn – neurite bundle innervating tentacular cirrus; vln – ventral longitudinal neurite bundle; vrcc – ventral root of circumoesophageal connective

**Fig. 10:**
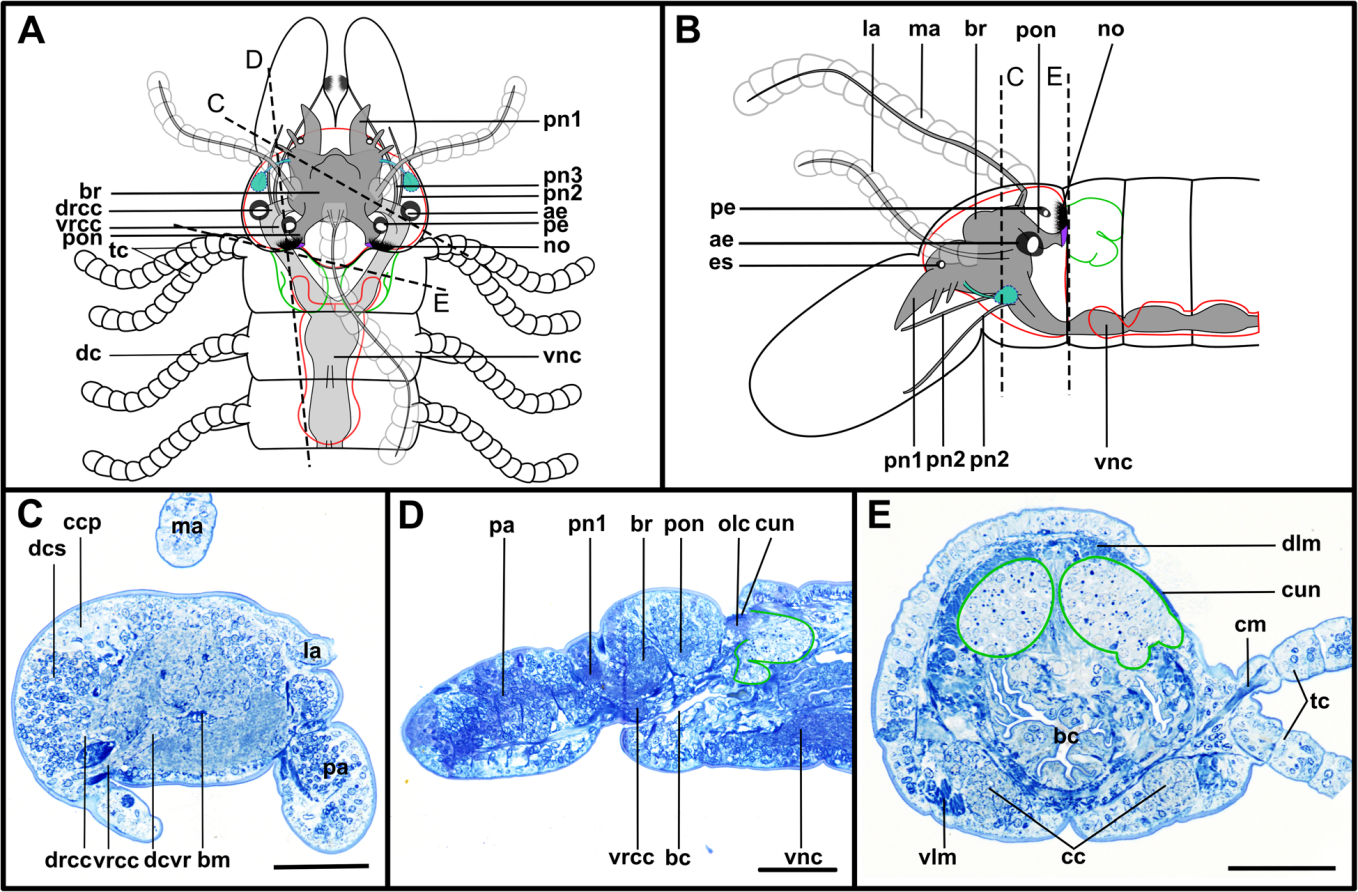
*Syllis garciai*. Brain and associated clusters of somata. **a and b**: Schemata of the anterior end of *S. garciai*g. Dotted lines indicate the position of the sections C, D and E. The neuropil of the brain is enveloped by somata (red). The nuchal lobes (green) are restricted to the achaetous segment. A pair of dorsal clusters of somata (turquoise) lie laterally to the brain. **c**: Semi-thin cross section of the brain, slightly oblique. Drcc and vrcc enter the brain. **d**: Semi-thin saggital section through the nuchal organ. Somata of primary receptor cells and somata of the brain are not distinguishable from each other. The nuchal lobes (green) extent posteriorly. **e**: Semi-thin cross section of the nuchal organ, slightly oblique. The prominent pair of nuchal lobes (green) lies dorsolaterally. **Scale bars = 50 μm. Abbreviations**: ae – anterior eye; bc – buccal cavity; bm – muscle penetrating the brain; br – brain; cc – circumoesophageal connective; ccp – cells of crescent shaped ciliary patch; cm – cirrus muscle; cun – cuticle of nuchal organ; dc – dorsal cirrus; dcs – dorsal cluster of somata; dcvr - dorsal commissure of vrcc; dlm – dorsal longitudinal muscles; drcc – dorsal root of circumoesophageal connective; es – eye spot; la – lateral antenna; ma – median antenna; no – nuchal organ; olc – olfactory chamber; pa – palp; pe – posterior eye; pn1–3 – palp neurote bundle 1–3; pon – posterior neurite bundle; stgn1–5 – stomatogastric neurite bundles 1–5; tc – tentacular cirrus; tcn – neurite bundle innervating tentacular cirrus; vlm – ventral longitudinal muscle; vnc – ventral nerve cord; vrcc – ventral root of circumoesophageal connective

**Fig. 11:**
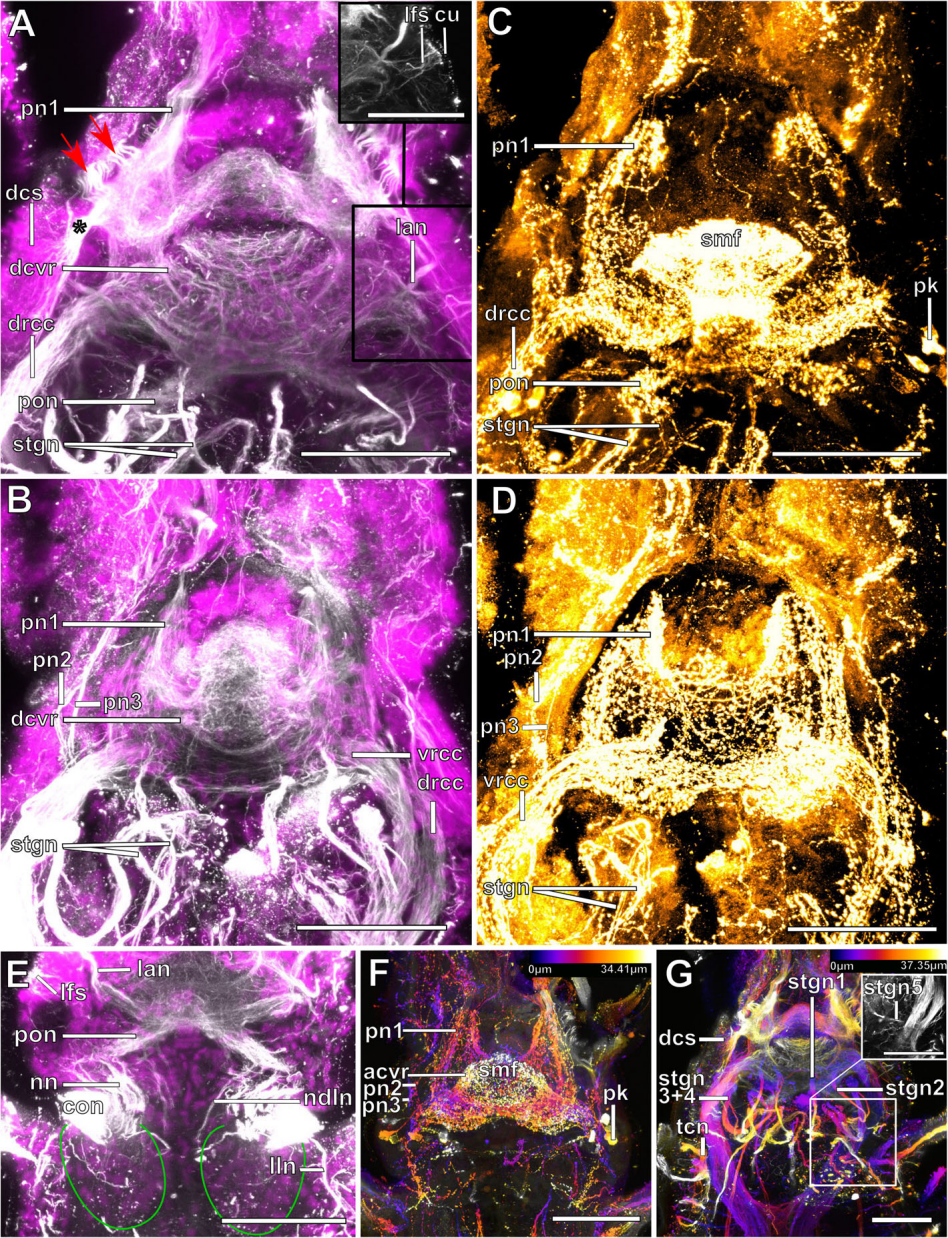
*Syllis garciai.* Innervation of prostomium and anterior segments. **a:** Maximum intensity z-projection of combined α-tubulin-lir (grey) and cell nuclei (magenta). Dorsal planes of the prostomium with inset of laterofrontal sense organ (α-tubulin-lir only). Several bundles (red arrows) separate from the main palp neurite bundle and form a plexus inside the palps. A neurite bundle (asterisk) reaches posteriorly at the transition of the main palp neurite bundle and the brain, into a cluster of dorsolateral somata. **b**: Maximum intensity z-projection of combined α-tubulin-lir and cell nuclei, ventral planes. Innervation of palps. **c**: Maximum intensity z-projection of serotonin-lir, dorsal planes of prostomium. The brain shows a strong serotonin-lir signal but separate commissures are hard to differenciate. **d**: Maximum intensity z-projection of combined serotonin-lir, ventral planes. All of the three neurite bundles innervating the palps are visible. **e**: Maximum intensity z-projection of α-tubulin-lir and cell nuclei. Detail of nuchal organ, dorsal. Fibres from primary sensory cells of the nuchal organ reach towards the posterior neurite bundles of the brain. The nuchal lobes (green line) lie behind the cilia of the nuchal organ. **f**: Colour coded z-projection of serotonin-lir, median planes. Palp nerves and serotonin-lir perykarya are visible. **g**: Colour coded z-projecion of α-tubulin-lir, ventral planes with inset of maximum intensity z-projection of α-tubulin-lir of stomatogastric neurite bundle number 5. Two stomatogastric neurite bundles leave the ventral part of the neuropil. Another two pairs emerge from the vrcc, fusing shortly after emergence, followed by a fifth small neurite bundle (in inset). **Scale bars = 50** μm**, insets = 25 μm. Abbreviations**: acvr – anterior dorsal commissure of vrcc; cu – cuticle; dcs – dorsolateral cluster of somata; dcvr - dorsal commissure of vrcc; drcc – dorsal root of circumoesophageal connective; lan – neurite bundle innervating lateral antenna; lfs – laterofrontal sense organ; lln – lateral longitudinal neurite bundle; ndln – fibres from nuchal organ joining dorsal longitudinal neurite bundle; nn – nuchal neurite bundle; pk – serotonin-lir perikarya; pn1 – main palp neurite bundle; pn2 – palp neurite bundle from drcc; pn3 – palp neurite bundle from vrcc; pon – posterior neurite bundle; smf – spherical median front of the brain; stgn1–5 – stomatogastric neurite bundles 1–5; tcn – neurite bundle innervating tentacular cirrus; vrcc – ventral root of circumoesophageal connective

The expansion of the somata of the brain differs between Syllinae and Exogoninae. A pair of posterior extensions of the neuropil (here termed posterior nerves) is present in all species (Figs. 2f, 3d, 4d, 5f, 6c, 7a, 8a, 9a, e, 10d, 11a., suppl. Figs. S2a, S3a, b). In Syllinae, *Streptosyllis* and Autolytinae they connect directly to the neurite bundles of the primary sensory cells of the nuchal organ or the nuchal eupalettes (Figs. 7a, 9a, e, 11e, suppl. Figs. S2a, S3a, b). The somata of the brain are restricted to the prostomium (Figs. 6a-d, 8a, b, d, 10a, b, d). In Exogoninae these posterior nerves are surrounded by a pair of dorsal lobes of brain somata. These lobes extend into the anterior segments to various degrees. In *Prosphaerosyllis marmarae* the dorsal lobes reach the second chaetiger (Figs. 2a, b, d, f, 3a, d), while in *Sphaerosyllis taylori* the lobes are shorter and only reach into the first chaetiger (Figs. 4a, b, d, 5a). The posterior nerves themselves are only recognizable to the first chaetigerous segment in both species (Figs. 3a, d, e, g, 5f). The connection of posterior nerves to the nuchal nerves is thought to be located within these dorsal lobes, but could not be observed with certainty.

Serotonin-like immunoreactive (lir) perikarya are found in several clusters among the rind of somata of the brain; some are found posterolaterally of the brain, others lie in a posteromedian position behind the brain (Figs. 5f, 7b, d, 9c, 11f). Due to the dense packing and variable staining of the serotonin-lir perikarya, their number could not be determined accurately for each species. It appears to be higher in the *Syllis* species than in *Plakosyllis* and both species of Exogoninae.

**Fig. 12:**
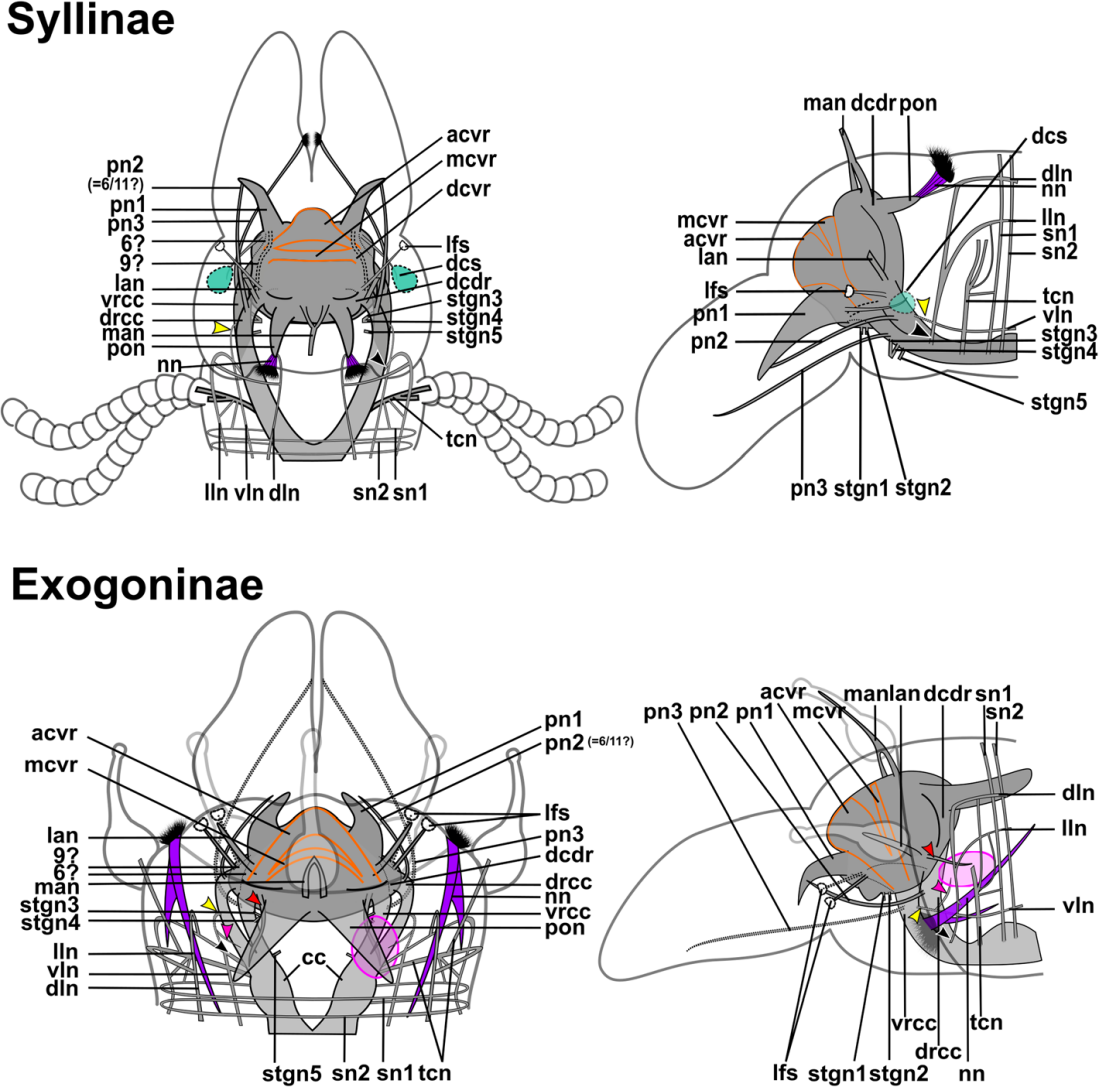
Exogoninae and Syllinae. Schemata of the innervation of the prostomium and achaetous segment. Structures not present in all species are shown in dotted lines, except for the dorsolateral cluster of somata, the laterofrontal sense organs and palp neurite bundle 3, which are not present in *Plakosyllis brevipes* but are shown in the schema in the Syllinae. The ventral root of the circumoesophageal connective forms a median dorsal and an anterior dorsal commissure most clearly visible in serotonin-lir (orange). The dorsal root of the circumoesophageal connective forms a posterior dorsal commissure, which sends fibres to the median antenna, the posterior neurite bundles and probably the lateral antennae. At least one pair of laterofrontal sense organs is located at the laterofrontal margins of the prostomium. The palps are innervated by a strong neurite bundle receiving fibres from the vrcc (possibly homologous to root 6) and possibly the drcc (possibly homologous to root 9), one neurite bundle coming from the drcc and one coming from the vrcc. The neurite bundle coming from the vrcc is missing in *Plakosyllis brevipes* and *Prosphaerosyllis marmarae*. Laterally to the main palp neurite bundle a small neurite bundle leads posteriorly towards a dorsolateral cluster of somata (turquoise) in the Syllinae. In the Exogoninae an inferior posterior cluster of somata (pink circle) receives fibres from the drcc (red arrowhead). The longitudinal neurite bundles originate from a neurite bundle marked by black arrowheads. The ventral longitudinal neurite bundle also receives fibres from a neurite bundle originating from the drcc (yellow arrowhead) which sends fibres to the posterior inferior cluster of somata (pink arrowhead) in *Sph. taylori*. Two segmental neurite bundles forming dorsal commissures are present on the outer margin of the achaetous segment. **Abbreviations**: acvr – anterior dorsal commissure of the vrcc; cc – circumoesophageal connective; dcdr – dorsal commissure of the drcc; dln – dorsa longitudinal neurite bundle; drcc – dorsal root of the circumoesophageal connective; lan – neurite bundle innervating lateral antenna; lfs – laterofrontal sense organ; lln – lateral longitudinal neurite bundle; man – neurite bundle innervating median antenna; mcvr – median dorsal commissure of the vrcc; nn – nuchal neurite bundle; pn1 – main neurite bundle innervating the palps which receives fibres from the vrcc and possibly the drcc; pn2 – neurite bundle innervating palps which originates from drcc; pn3 – neurite bundle innervating palps which originates from vrcc (presence uncertain in *Prosphaerosyllis marmarae*); pon – posterior neurite bundles of the brain; sn1, sn2 – segmental neurite bundles of achaetous segment; tcn – neurite bundle innervating tentacular cirrus/cirri; vln – ventral longitudinal neurite bundle; vrcc – ventral root of circumoesophageal connective; 6 – fibres possibly homologous to palp neurite bundle root 6 from vcvr; 9 – fibres possibly homologous to palp neurite bundle root 9 from drcc

The microanatomy of the brain and the origin of longitudinal nerves is basically the same in all species and is summarised in Fig. 12. The brain has a basiepithelial position and an extracellular matrix separating the somata of the brain from the epidermis of the prostomium could not be observed (Figs. 2c, d, f, 4c, d, 6c, d, 8c, d, 10c, d). Only the ventral parts are separated from the underlying mesodermal tissues.

Due to the density of the neuropil it is difficult to distinguish individual commissures within the brain. The dorsal commissure (dcvr, in [32]) of the vrcc was observed both in Syllinae and Exogoninae (Fig. 12). It is visible in histological sections (Figs. 2c, 4c, 8c, 10c) as well as in serotonin-lir and α-tubulin-lir stainings (Figs. 5c, g, 11a, b). The dcvr extends dorsally comprising the spherical median front of the brain (Figs. 9c, 11c, f), above a muscle bundle penetrating the brain. Sometimes a median and anterior dorsal commissure of the dcvr was observed (Figs 5f, 9f). Other fibres of the vrcc reach to the ventrofrontal part of the brain, sending fibres inside the palps. The drcc also splits. Some fibres join the dcvr, while others form a commissure (dcdr, in [32]) from which the posterior nerves arise. The origins of the fibres comprising the rest of the neuropil could not be traced. The eyes are directly innervated from lateral regions of the brain, but it could not be detected whether they are connected to one of these roots.

Each lateral antenna is innervated by one neurite bundle that originates from the lateral regions of the brain, slightly dorsoposterior to where the drcc enters the neuropil (Figs. 7a, b, 8c, 9a, 11a, suppl. Figs. S2a, S3a, b), probably corresponding to the dorsal commissure of the drcc. The median antenna is innervated by two neurite bundles coming from the posterior region of the brain (Fig. 3e, also corresponding to the dcdr (data not shown), suppl. Figs. S2a, S3a, b).

The innervation of the tentacular cirri (only one pair in *Sphaerosyllis taylori*, two pairs in the other species) is also consistent in all Syllidae. A neurite bundle, which emerges from the circumoesophageal connective after the vrcc and drcc have fused, splits and innervates both cirri (Figs. 5e, 7c, d, 9b, g). In *Prosphaerosyllis marmarae* the neurite bundle of the tentacular cirri is accompanied by two clusters of somata (Figs. 2a, b, 3b, c), which are not present in any of the other species.

The palps are innervated by a strong palp neurite bundle (pn1) coming directly from the neuropil of the brain (Figs. 3a, b, e, 5c, d, 7c, d, 9a, b, e, 11a-d, f). Fibres originate mainly from the vrcc. A few fibres originate from the drcc at a point where the drcc has already entered the neuropil of the brain and join the main palp neurite bundle (Fig. 7c). Several neurite bundles branch off pn1 and enter the palps in the *Syllis* species (3–4 in *Syllis garciai* and 2 in *Syllis tyrrhena*) (Figs. 9a, b, 11a). These are visible in both, serial semi-thin sections, and serotonin-lir and α-tubulin-lir stainings. Inside the palps the neurite bundles ramify further, forming an elaborate plexus.

Two additional neurite bundles, pn2 and pn3, enter the palps. While pn2 branches off the drcc shortly before it enters the brain (Figs. 3b, e, 5d, 7c, 9b, g, 11d), pn3 branches off the vrcc (Figs. 3c, 5e, 9g, 11b, d). Pn2 fuses with pn1 once it enters the palps. Pn3 runs along the ventral part of the palps and reaches to the ciliary patches at the inner margins of the palps in *Syllis tyrrhena* and *Syllis garciai*. It is present in *Sphaerosyllis taylori* as well, which does not possess similar ciliary patches. In *Sph. taylori* it reaches to a network of small neurite bundles more proximal than the ciliary patches in the *Syllis* species. It is not clear if pn3 exists in *Prosphaerosyllis marmarae.* If so, it is very short and connects to pn1 close to its base, which could only be observed in one specimen. In *Plakosyllis brevipes* pn3 could not be found.

In both *Syllis* species a conspicuous neurite bundle leaves the neuropil anteriolaterally to the main palp neurite bundles and runs posteriorly along the lateral margins of the prostomium, where it branches several times (Figs. 9a, 11a). The somata in this region are densely packed (Fig. 11a), but do not appear smaller or globuli-like. Neurite bundles from the drcc reach into this region (Fig. 9a). In *Plakosyllis brevipes* and both Exogoninae, a similar dorsal cluster of somata was not found. No distinct mushroom body-like structures with globuli-like cell clusters were identified in any of the species.

A pair of laterofrontal sense organs was observed in all species except *Plakosyllis brevipes*. In *Sphaerosyllis taylori* two pairs of α-tubulin-lir sense organs lie at the laterofrontal border of the prostomium (Fig. 5d). In *Prosphaerosyllis marmarae* one pair of similar sense organs is present, but their neurite bundles originate directly from the drcc (Figs. 3e, g), while in *Sph. taylori* they emerge from the brain, close to where the drcc enters the neuropil. The same kind of sense organs were found in *Syllis garciai* and *Syllis tyrrhena* where the neurite bundle leaves the neuropil just above the drcc underneath the lateral antenna and reaches the lateral margin of the prostomium just in front of the anterior lense eyes (Figs. 9e, 11a inset). The neurite bundles very likely do not possess cilia penetrating the cuticle since no external cilia are visible in this region (Fig. 11a inset). In *P. brevipes,* these sense organs could not be identified with certainty. Strongly innervated regions in the prostomium of *Myrianida* were observed but it is unclear if these structures are the same as in the other syllids (suppl Figs. S3b). *Streptosyllis* has a ciliary patch innervated by the drcc which could be similar to the laterofrontal sense organ, but its cilia clearly penetrate the cuticle (Suppl. Fig. S2a).

### Nuchal organs and posterior inferior clusters of somata

**Fig. 13:**
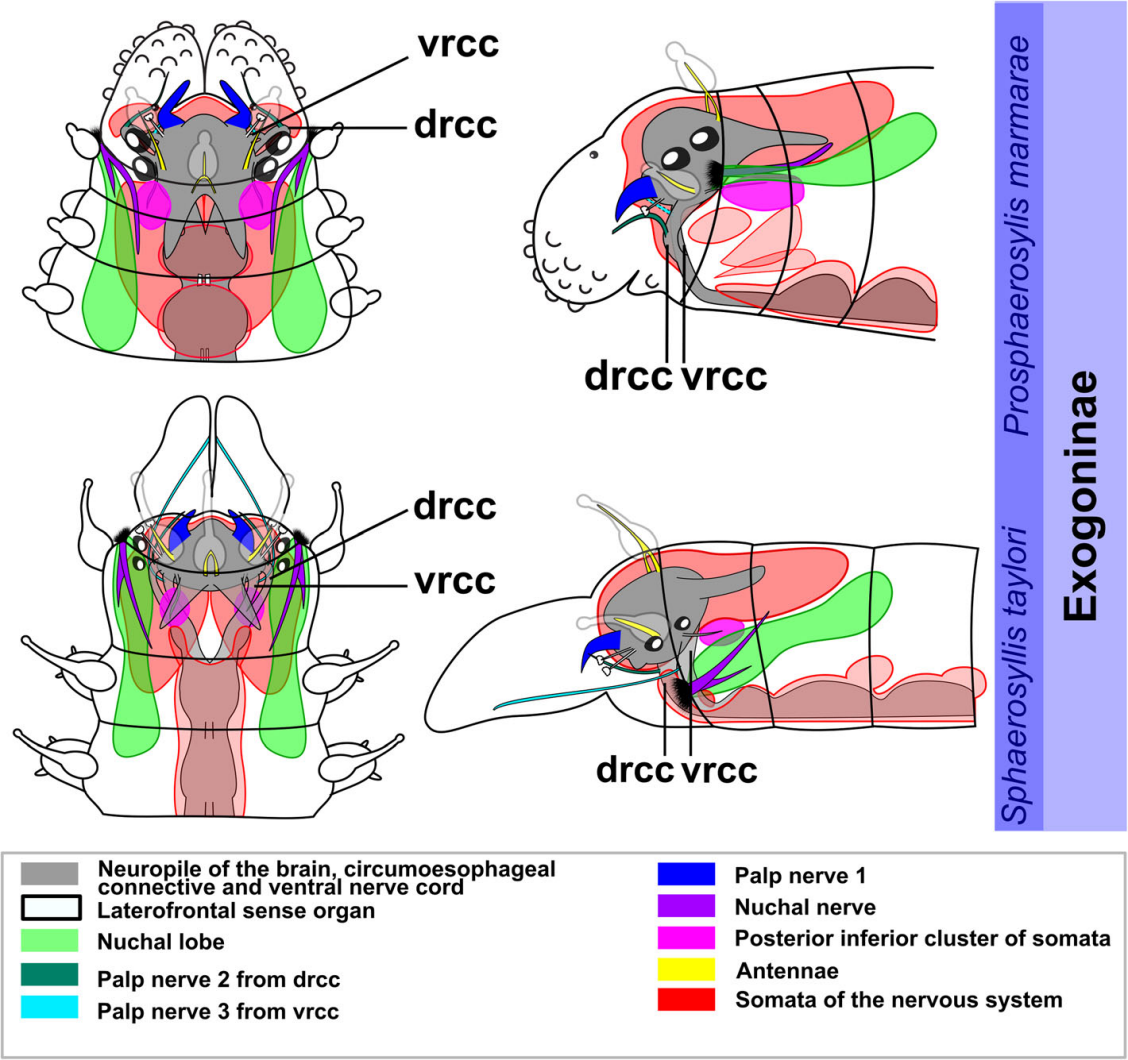
Overview of the anterior nervous system and associated lobes of the Exogoninae. Both Exogoninae have a pair of dorsal lobes consisting of somata of the brain, reaching inside the anterior segments and a pair of nuchal lobes, consisting of support cells of the nuchal organ, which reach the second chaetiger. Four clusters of somata are present in the tentacular segment and first chaetigers of *Prosphaerosyllis marmarae* and one in *Sphaerosyllis taylori*. The palps are innervated by at least a main neurite bundle and a neurite bundle from the dorsal root of the circumoesophageal connective. A third neurite bundle reaches from the ventral root of the circumoesophageal connective inside the palps (uncertain in *Prosphaerosyllis marmarae*). A distinct circumoesophageal ganglion is not present. Somata are clustered on the ventral side of the circumoesophageal connective, fusing with the ganglion of the first chaetiger. **Abbreviations**: drcc – dorsal circumoesophageal connective; vrcc – ventral circumoesophageal connective

**Fig. 14:**
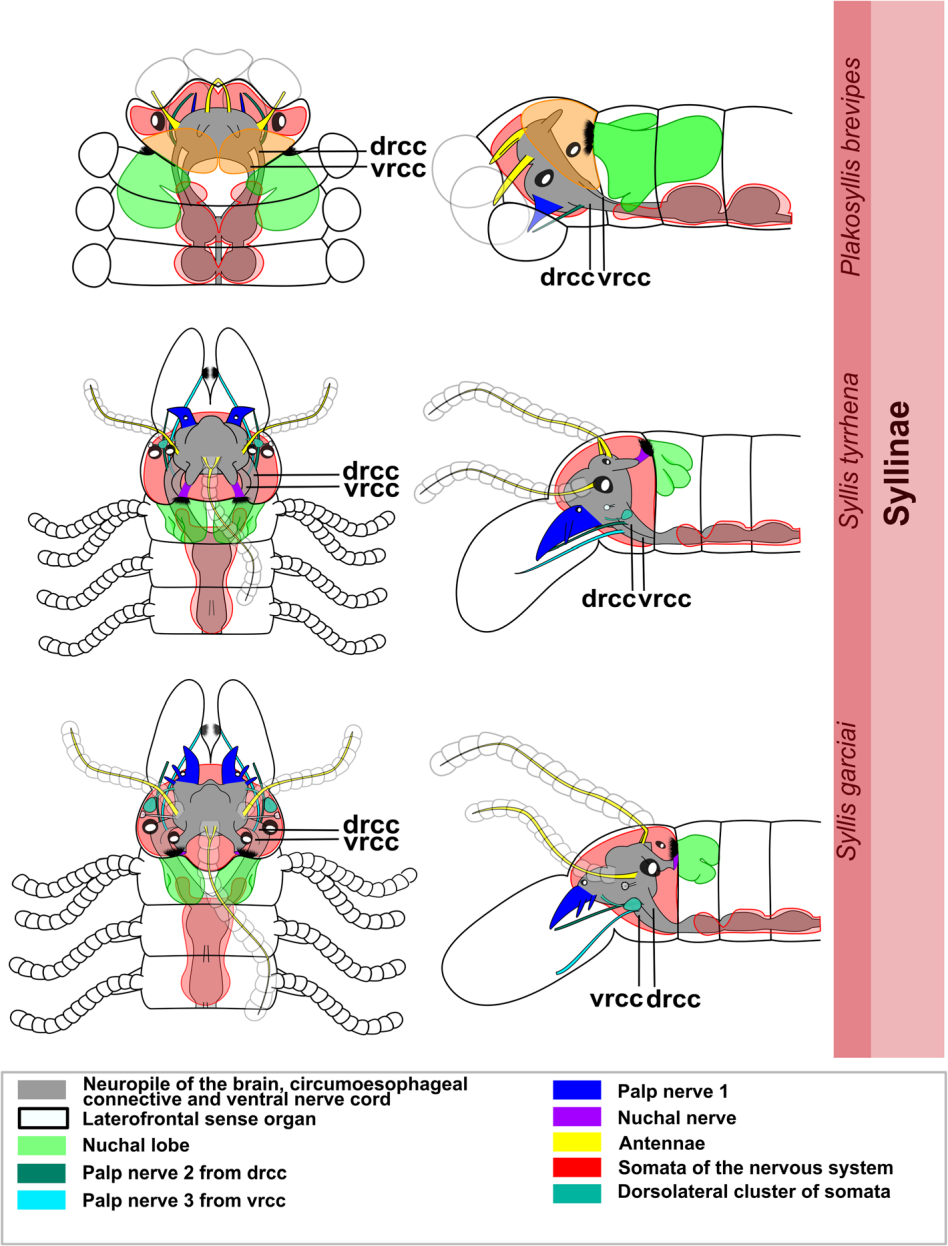
Overview of the anterior nervous system and associated lobes of the Syllinae. All species possess a pair of nuchal lobes which are restricted to the achaetous segment and consist of support cells of the nuchal organ. Dorsolateral clusters of somata were only found in the *Syllis* species. The palps are innervated by at least a main neurite bundle and a neurite bundle from the dorsal root of the circumoesophageal connective. A third neurite bundle reaches from the ventral root of the circumoesophageal connective inside the palps, which is missing in *Plakosyllis brevipes*. A distinct circumoesophageal ganglion is not present. Somata are clustered on the ventral side of the circumoesophageal connective, fusing with the ganglion of the first chaetiger. **Abbreviations**: drcc – dorsal circumoesophageal connective; vrcc – ventral circumoesophageal connective

The distribution of dorsal and nuchal lobes and other clusters of somata is summarized in Figs. 13 and 14. In addition to a pair of dorsal lobes in the Exogoninae, a pair of nuchal lobes is present in all of the five more closely investigated species. It can only be clearly observed in histological sections. The nuchal lobes presumably consist of the nuclei of supportive cells from the nuchal organ and are histologically distinct from the dorsal lobes of the Exogoninae and the somata of sensory cells (Figs. 2d-f, 4d, e, 5h, 6d, 8d, e, 10d, e). In stainings with nuclear markers, the nuclei in the nuchal lobes are difficult to observe as they are set further apart than e.g. the nuclei of the dorsal lobes (Figs. 3a, b, 5a-c, 9e, 11e).

In both *Syllis* species the nuchal organ lies dorsally behind the prostomium in form of a pair of ciliated pits (Figs. 8a, b, d, e, 9a, e, 10a, b, d, e, 11e). Somata of receptor cells and somata of the brain lie between the nuchal organ and the neuropil of the brain. The somata of these cells are not clearly distinguishable from each other in histological semi-thin sections. Several neurite bundles run directly from the nuchal organ to the posterior neurite bundle of the brain (Figs. 9a, e, f, 11e). The nuchal lobes lie behind the nuchal organ in the first achaetous segment (Figs. 8a, b, d, 10a, b, d). Each lobe is constricted by muscle bundles separating it into two to three bulbs. In *Myrianida* (Autolytinae), which has a pair of nuchal eupalettes, the innervation is similar to the Syllinae; the brain is directly connected to the primary sensory cells of the nuchal eupalettes via its posterior neurite bundles (suppl. Fig. S3a). The innervation of the nuchal organs in *Streptosyllis websteri* also resembles the situation in the Syllinae (suppl. Fig. S2a).

In *Plakosyllis brevipes* the nuchal organs lie laterally across the border of the prostomium and first achaetous segment (Figs. 6a, b, d, 7a). The nuchal lobes extend into the first achaetous segment and are separated into two bulbs (Figs. 6a, b, d). Unlike the *Syllis* species, the somata of the primary sensory cells of the nuchal organ and the somata of the brain are clearly separated and distinct in histological sections (Fig. 6c, d). The somata of the sensory cells form a pair of postero-lateral lobes between nuchal organ and brain (Fig. 6a, b, d), from which several neurite bundles visible in α-tubulin-lir stainings connect the nuchal organ to the posterior neurite bundles of the brain (Fig. 7a).

In the Exogoninae the nuchal organ also lies laterally between prostomium and the first achaetous segment (Figs. 2a, b, e, 3c-g, 4a, b, 5e, g). The nuchal lobes in these species extend far posteriorly and reach into the second chaetigerous segment. A distinct neurite bundle extends posteriorly from the nuchal organ forming the nuchal neurite bundle (Figs. 3c-f, j, 5b, c, d). Several fine α-tubulin-lir fibres separate from the nuchal neurite bundle and extend into the nuchal lobes (Fig. 3d, i). Other neurite bundles reach into a pair of posterior inferior clusters of somata that are only present in both Exogoninae, and which also receive a neurite bundle from the drcc (Fig. 3a, c, i, 5b, c). This posterior inferior cluster of somata possibly consists of the somata of the primary sensory cells of the nuchal organ. Only in *Prosphaerosyllis marmarae* another small cluster of somata lies ventrally to the posterior inferior cluster, sending neurite bundles to the drcc (Figs. 2a, b, d, f, 3b, c). Thus there are all in all four clusters of somata (two tentacular clusters, one inferior posterior cluster and a fourth small cluster) in addition to the dorsal lobes and nuchal lobes present in this species (Figs. 2d, f, 3a-c). Several branches of the nuchal neurite bundle reach into the dorsal lobe and seem to connect to the posterior neurite bundles of the brain. In contrast to the Syllinae, a direct connection between the nuchal neurite bundle and the posterior neurite bundles of the brain is not clearly visible in α-tubulin- or serotonin- lir staining in the Exogoninae. In semi-thin sections of *Prosphaerosyllis marmarae* it seems like neurite bundles of both the sensory cells and the posterior neurite bundles of the brain can be observed in the posterior lobes (Fig. 2f). TEM observations would be necessary to confirm this, since the resolution in light microscopy is not high enough to link somata with their neurites in semi-thin sections.

### The stomatogastric nervous system

All Syllidae possess a characteristic pharynx or axial proboscis comprising a pharyngeal tube followed by the so called proventricle, a muscular part of the gut considered as apomorphy of the family [47]. The anterior part of the pharynx is cuticularized and can be everted.

The pharyngeal sheath and the pharynx are innervated by several distinct neurite bundles, all of which are located intraepithelially in the epithelium of the foregut (unpubl. TEM observations). Two neurite bundles form distinct ring-like commissures around the pharyngeal tube (observed in *Syllis garciai*, *Syllis tyrrhena*, *Plakosyllis brevipes*, *Prosphaerosyllis marmarae*, *Sphaerosyllis taylori*, *Streptosyllis websteri* (suppl. Fig. S2b)*, Streptosyllis sp.* (Autolytinae), *Brania clavata, Brania pulsilla, Exogone naidina, Parapionosyllis labronica* and *Sphaerosyllis tetralix* (Exogoninae) (data not shown). The innervation of the pharyngeal tube and pharyngeal sheath is similar among at least the Anoplosyllinae, Exogoninae and Syllinae and thus does not depend on the presence or absence of a pharyngeal tooth. Unfortunately, due to strong autofluorescence of the cuticle of the pharynx and weak staining of the stomatogastric neurite bundles, the innervation of the pharynx could not be reconstructed for the Autolytinae (suppl. Fig. S3a).

**Fig. 15:**
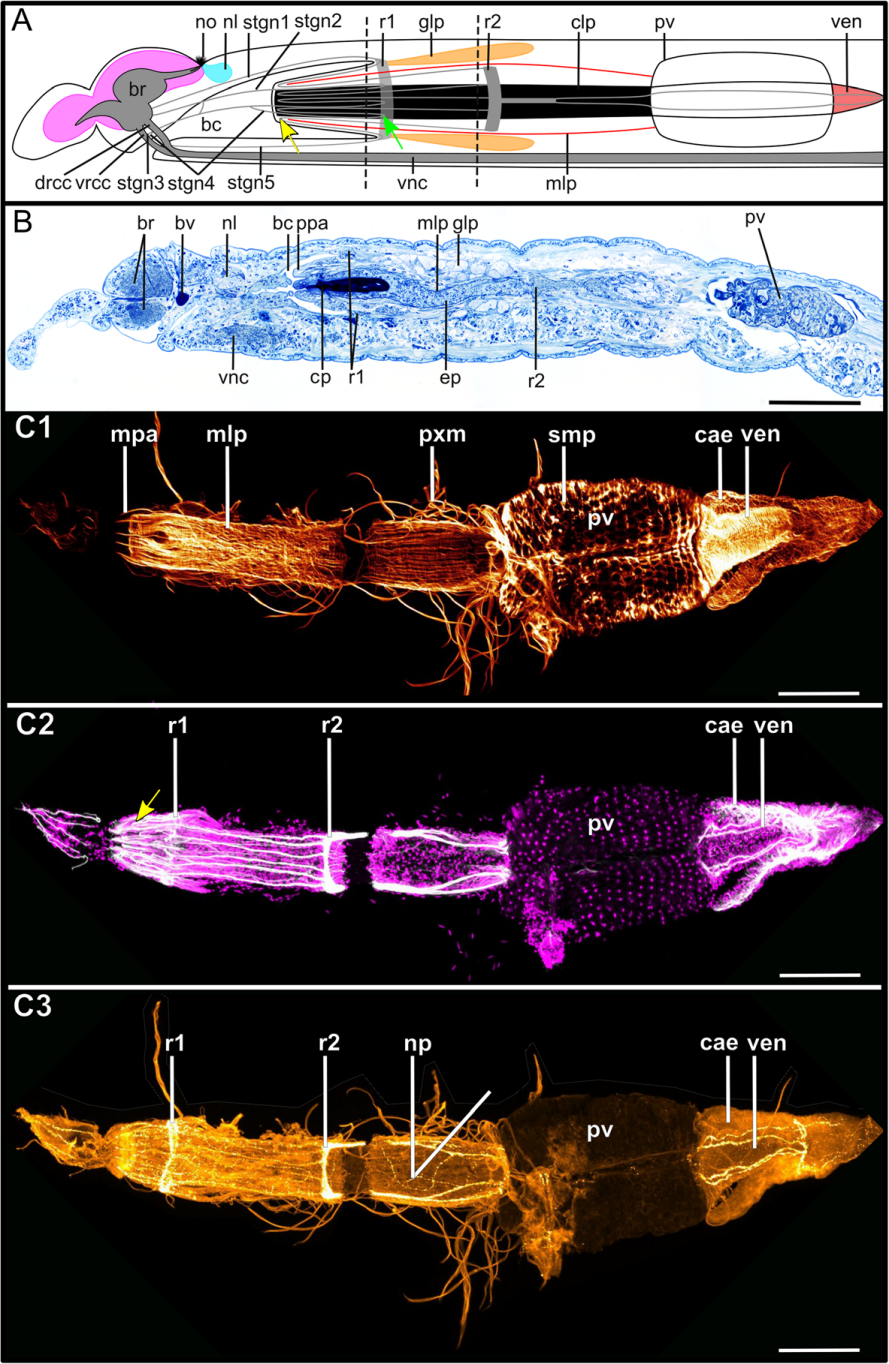
*Syllis tyrrhena*. Innervation of the stomatogastric nervous system. **a**: Schema of the stomatogastric nervous system. Five pairs of stomatogastric neurite bundles are present in the anterior part of the digestive tract. Stgn 1 and 2 originate from the brain, stgn 3 and 4 from the ventral root of the circumoesophageal connective and stgn 5 from a region just after drcc and vrcc have fused. They are interconnected by several links. The stomatogastric neurite bundles lead posteriorly until reaching a first ring neurite bundle (r1) in a region where the epithelium of the buccal cavity and the pharynx fuse. Here the neurite bundles turn (green arrow) towards the beginning of the pharynx. At the anterior end of the pharynx the stomatogastric neurite bundles again turn course posteriorly (yellow arrow), entering the pharyngeal epithelium underneath the muscular layer of the pharynx. The neurite bundles then meet a second pharyngeal ring neurite bundle. From there a pair of thick neurite bundles continues in direction of the proventricle, branching again into two bundles, which then enter proventricle and ventricle. Some species possess pharyngeal glands, which are sac like tubes sitting approximately at the region of the first ring neurite bundle. **b**: Semi-thin saggital section through the anterior digestive system of *S. tyrrhena* showing the different histological layers of the pharyngeal tube and the pharyngeal glands. **C1-C3**: Maximum intensity z-projections of the dissected pharynx, proventricle and ventricle of *S. tyrrhena*. **C1**: F-actin staining. **C2:** α-tubulin-lir (grey) and cell nuclei (magenta). **C3**: Serotonin-lir. The individual neurite bundles are ambiguous in serotonin-lir, the intensity of the signal differing between stainings. The serotonin-lir nervous plexus of the pharynx and both stomatogastric ring neurite bundles are clearly visible as is the innervation of the ventricle. **Scale bars = 100** μm. **Abbreviations**: br – brain; bc – buccal cavity; bv – blood vessel; cae – caecum;cp – cuticularised layer of the pharynx; drcc – dorsal root of circumoesophageal connective; ep – epidermal layer of pharynx; glp – pharyngeal glands; mlp – muscular layer of pharynx; mpa – muscles of pharyngeal papillae; pxm – pharynx muscles; nl – nuchal lobe; np – nervous plexus; no – nuchal organ; ppa – pharyngeal papilla; psh – pharyngeal sheath; pv – proventricle; pxm – pharynx muscles; r1 – first stomatogastric ring neurite bundle; r2 – second stomatogastric ring neurite bundle; smp – striated muscles of proventricle; stgn1–5 – stomatogastric neurite bundles 1–5; ven – ventricle; vnc – ventral nerve cord; vrcc – ventral root of circumoesophageal connective

Initially five pairs of stomatogastric neurite bundles (stgn) (Fig. 15) emerge from the brain and run posteriorly following the epithelium of the pharyngeal sheath until they join into the first ring neurite bundle (Fig. 15a). Stgn 1 and 2 originate from the ventral part of the neuropil of the brain, while the other three emerge from the vrcc, the 5th one shortly before vrcc and drcc fuse (Figs. 3j, 5e, 7c, 9f, g, 11g, 12, 15). Stgn 3 and 4 fuse and separate again posteriorly. The first ring neurite bundle lies where the epithelium of the pharyngeal sheath passes into the epithelium surrounding the pharyngeal tube (Fig. 15a, b).

**Fig. 16:**
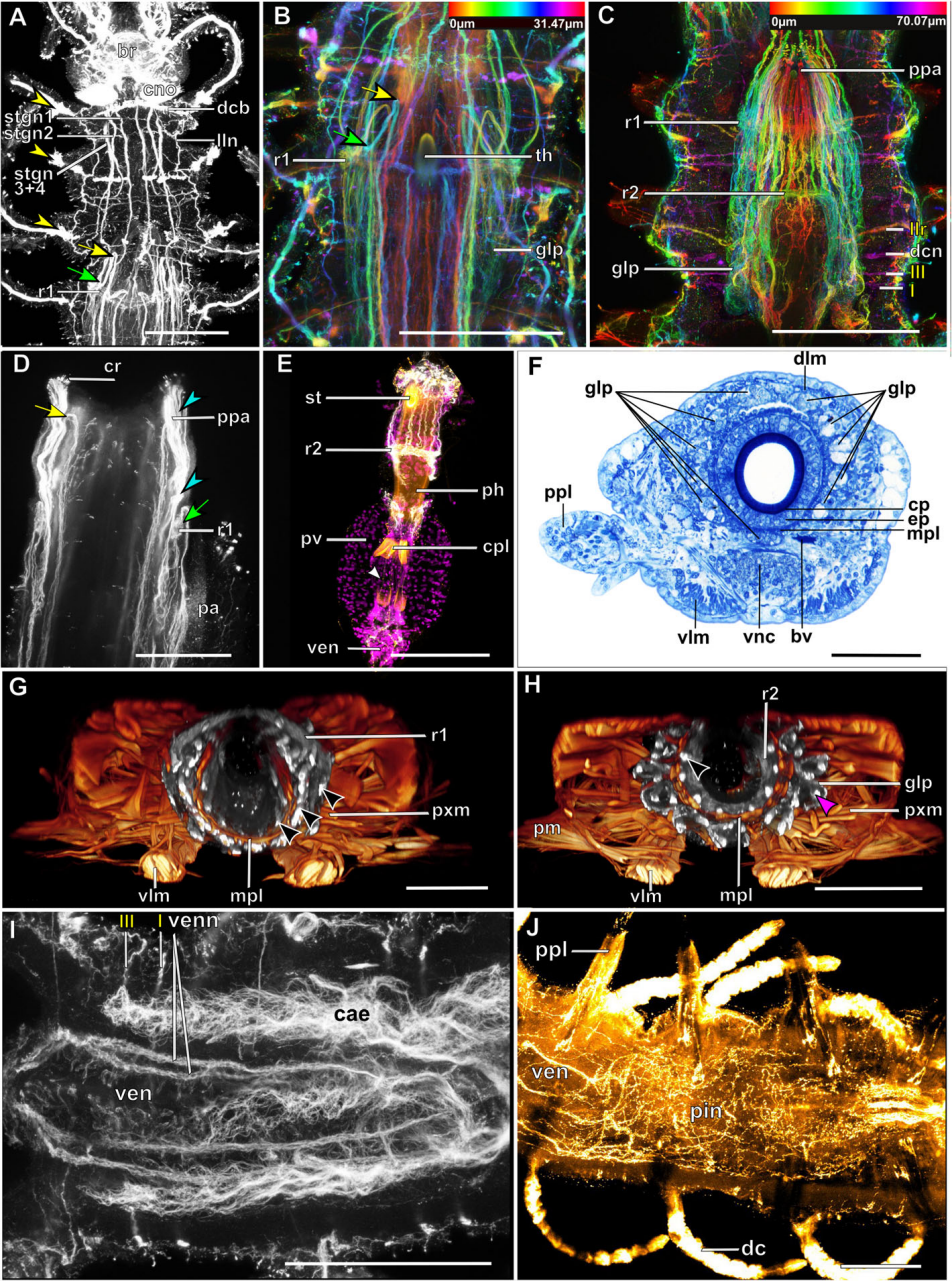
Details of the stomatogastric nervous system in Syllidae. **a**: *Syllis tyrrhena*. Maximum intensity z-projection of α-tubulin-lir, dorsal planes, anterior segments. Green arrow: stomatogastric neurite bundles turning towards the anterior end of the pharynx. Yellow arrow: stomatogastric neurite bundles turning posteriorly underneath the muscular layer of the parynx. Yellow arrowheads: ciliary patches on the first three chaetigers. **b**: *S. tyrrhena.* Colour coded z-projection of α-tubulin-lir. Detail of neurite bundles entering pharynx. **c:***Sphaerosyllis taylori*. Colour coded z-projection of α-tubulin-lir. Detail of the pharynx and pharyngeal gland. The pharyngeal glands show a strong α-tubulin-lir signal in this species. **d**: *Syllis garciai*. Detail of everted pharynx. The pharyngeal papillae are covered in ciliary receptor cells, innervated by numerous neurite bundles (turquoise arrowhead) branching off of the stomatogastric neurite bundles. **e**: *Plakosyllis brevipes.* Maximum intensity z-projection of α-tubulin-lir (grey), serotonin-lir (orange) and cell nuclei (magenta) of the dissected pharynx. Autofluorescence shows four plates inside the proventricle. White arrowhead: neurite bundles traversing the proventricle. **f:***S. tyrrhena*. Semi-thin cross section through the pharyngeal glands and layers of pharyngeal sheath. **g, h:***Sph. taylori.* Volume rendering of f-actin (glow) and α-tubulin-lir of the pharynx*.* The pharyngeal innervation and pharyngeal glands were segmented, other neurites are omitted. **g**: Cross section shortly before the first segmental ring neurite bundle. Black arrowheads: three layers of neurite bundles are visible. **h**: Cross section shortly before second pharyngeal ring neurite bundle. Black arrowhead: one layer of stomatogastric neurite bundles is visible. Pink arrowhead: signal inside the pharyngeal glands. **i**: *S. tyrrhena.* Maximum intensity z-projection of α-tubulin-lir. Detail of the ventricle and its four thick neurite bundles. **j**: *S. tyrrhena.* Maximum-intensity z-projection of serotonin-lir. Intestine just behind the ventricle. **Scale bars: A-C = 100** μm**, D = 50 μm, E = 100 μm, F-H = 50 μm, I, G = 100 μm**. **Abbreviations**: br – brain; bv – blood vessel; cno – cilia of support cells of nuchal organ; cp – cuticularised layer of pharynx; cpl – cuticularised plates inside proventricle of *P. brevipes*; cr – ciliary receptor; dcb – dorsal ciliary band; dlm – dorsal longitudinal muscles; ep – epidermal layer of pharyx; glp – pharyngeal glands; lln – lateral longitudinal neurite bundle; mlp – muscular layer of pharynx; ppa – pharyngeal papilla; ppl – parapodial lobe; ph – pharynx; pin – nervous plexus of the intestine; pm – parapodial muscle; pxm – muscles of the pharynx; r1 – stomatogastric ring neurite bundle 1; r2 – stomatogastric ring neurite bundle 2; stgn1–5 – stomatogastric neurite bundles 1–5; th – tooth; venn – neurite bundles innervating ventricle; ven – ventricle; vlm – ventral longitudinal muscles; vnc – ventral nerve cord. **Segmental neurite bundles in yellow**: I – segmental neurite bundle I; IIr – ring commissure of segmental neurite bundle II; III – segmental neurite bundle III

The stomatogastric neurite bundles then project anteriorly in the outer epithelium of the pharyngeal tube and form a hair-pin like loop at the most anterior tip of the pharynx (Figs. 15a, c2, 16a-d, g). Some of the fibers proceed anteriorly into the pharyngeal papillae. These are part of primary receptor cells present in the papillae [27]. Their neurite bundles are connected to the forward projecting neurite bundles between the anteriormost hair-pin like loop and the first ring commissure (Fig. 16d).

From their anteriormost position the stomatogastric neurite bundles again project posteriorly underneath the muscular layer of the pharynx and reach a second pharyngeal ring commissure (Figs. 15a, c2, 16c, e, h). From here only two latero-median neurite bundles continue posteriorly, each of which splits again into two neurite bundles. Thus, two pairs of neurite bundles enter the proventricle and the ventricle (Figs. 15a, c2, c3, 16i). These neurite bundles could be best traced in dissected proventricles of *P. brevipes* (Fig. 16e) and *Syllis tyrrhena*. In addition to the five pairs of stomatogastric neurite bundles a diffuse nervous plexus linking the stomatogastric neurite bundles to each other is also present in the inner epithelium of the pahrynx. *Prosphaerosyllis marmarae* is the only species in which a third ring neurite bundle is visible in serotonin-lir (Fig. 3g, h). It lies underneath the muscular layer of the pharynx at its anterior end.

**Fig. 17:**
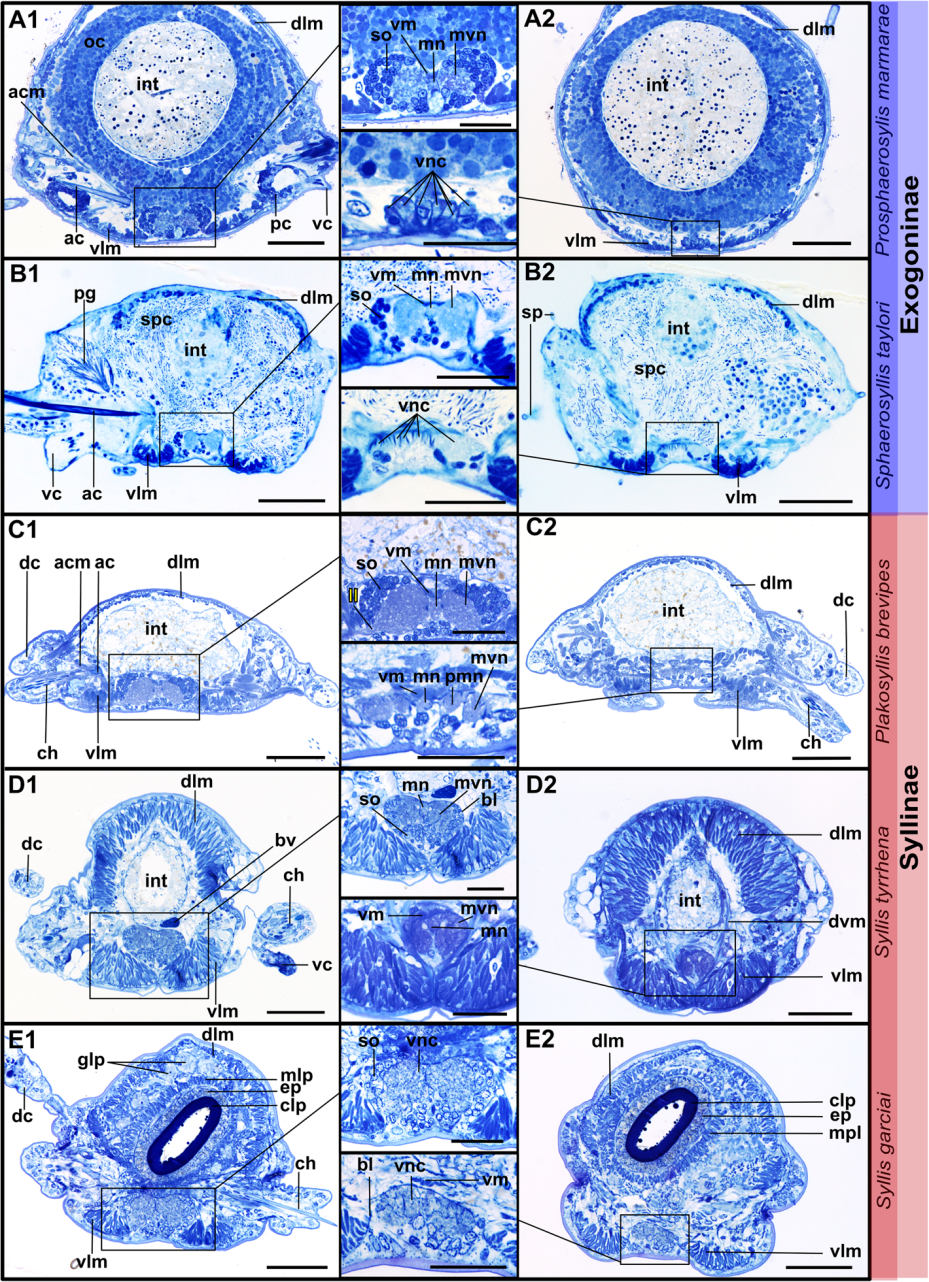
Histology of the ventral nerve cord. Semi-thin cross sections, toluidine blue staining. On the left are sections through the ganglion, on the right sections through the connectives, in the middle details of the ventral nerve cord. **a1, a2:***Prosphaerosyllis marmarae*. The ventral nerve cord is separated into three ventral nerves in the region of the ganglion, a median nerve and two main nerves. The connectives are separated into several bundles (8 or 9), but a consistent number could not be identified. The bundles lie adjacent to each other. **b1, b2**: *Sphaerosyllis taylori*. Inside the ganglion the ventral nerve cord is separated into three bundles, too. The connectives are separated into several bundles, similar to *Pr. Marmarae,* but impossible to count. **c1, c2**: *Plakosyllis brevipes*. Three nerves can be recognised inside the ganglion and five connectives are present between ganglia, which are hardly touching each other. Segmental neurite bundle II branches off the ganglion. **d1, d2***: Syllis tyrrhena*. Three nerves can be recognised inside the ganglion, but may fuse in some regions. Separate neurite bundles are difficult to distinguish in the connective, which seem to be fused. **e1, e2**: *Syllis garciai*. No cross sections of regions behind the proventricle were produced of *S. garciai*. In anterior sections separate nerves are neither in the ganglion nor in the connectives clearly distinguishable. **Scale bars: Overview = 50** μm**, insets = 25 μm**. **Abbreviations:** ac – acicle; acm – acicular muscle; bl – extracellular matrix; bv – blood vessel; ch – chaetae; cp – cuticularised layer of pharynx; dc – dorsal cirrus; dlm – dorsal longitudinal muscle; dvm – dorsoventral muscles; ep – epidermal layer of pharynx; glp – paryngeal glands; int – intestine; mn – median nerve; mlp – muscular layer of pharynx; mvn – main ventral nerve; oc – oocyte; pg – parapodial gland; pmn – paramedian ventral nerve; so – somata; sp. – sensory papilla; spc – spermatocyte; vc – ventral cirrus; vlm – ventral longitudinal muscle; vm – median longitudinal muscle above ventral nerve cord. **Segmental neurite bundles in yellow**: II – segmental neurite bundle II

Eleven sac-like tubes extend posteriorly from the position of the first ring neurite bundle (Figs. 15a, b, 16b, d, f, h, 17e1) which are only missing in *Prosphaerosyllis marmarae*. The tubes have been described as pharyngeal glands [50–52], which open on the pharyngeal papillae at the beginning of the pharyngeal tube [51]. The glands show a diffuse α-tubulin-lir signal (Fig. 16b, c, h). It is unclear whether they are innervated or if the signal belongs to cilia in the gland cells.

Only in *Prosphaerosyllis marmarae* stgn1 emerges from the front of the brain instead of the ventroposterior part. From its origin in the neuropil it first extends to the anterior edge of the prostomium and then turns posteriorly (Fig. 3j, e). In *Pr. marmarae* stomatogastric neurite bundle 2,3 and fuse, then separate again into two bundles, with one bundle reaching backward, while the other one reaches forward into the buccal lips (Fig. 3j). It then turns posteriorly towards the pharynx and separates again into two bundles, with five pairs of neurite bundles reaching the first ring neurite bundle. In *Syllis garciai* stgn 3 and 4 also reach forward into the buccal lips, before turning posteriorly (Fig. 11b, g). The intestine itself shows a diffuse serotonin-lir signal (16 J).

### Ventral nerve cord

The ventral nerve cord lies in a basiepithelial position and is encompassed by the extracellular matrix (Fig. 17d1 inset, e2 inset). In both *Syllis* species the ventral nerve cord is pushed inside the body cavity by the strongly developed ventral longitudinal muscles (Fig. 17d1, d2). The ventral nerve cord remains basiepithelially, surrounded by the extracellular matrix even if it lies dorsally of the ventral longitudinal muscle bundles as in *e.g. Syllis tyrrhena*. The ventral nerve cord in *Plakosyllis brevipes*, *Prosphaerosyllis marmarae* and *Sphaerosyllis taylori *remains in its ventral position and segmental nerves branch off laterally, while in *Syllis* species the segmental neurite bundles branch of laterally from the ventral nerve cord, turn immediately ventrally and run through the gap between ventral longitudinal muscle bundles (Figs. 17a1-c2, 18, 19a2-a5, b2, c2, 20d, 21, 22a1, a2, b1, b2). They then turn laterally and proceed into the periphery and parapodia.

**Fig. 18:**
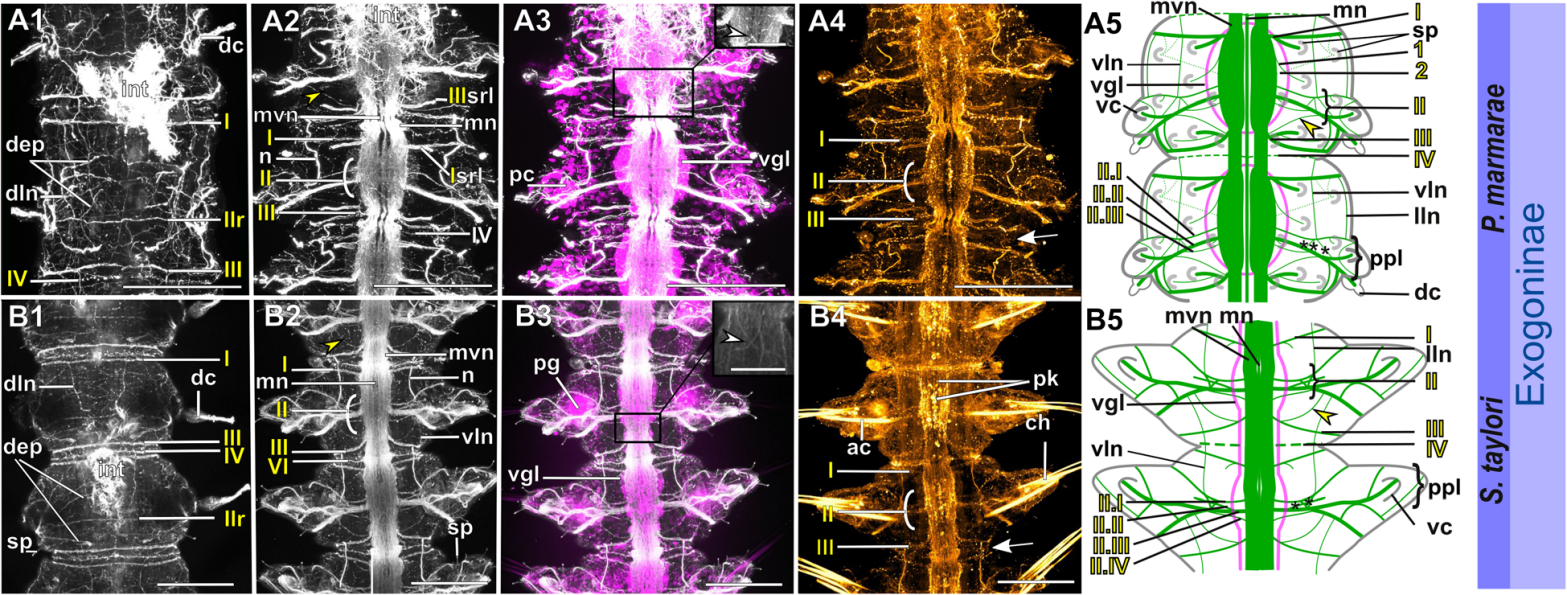
Exogoninae. Segmental neurite bundles. **a***Prosphaerosyllis marmarae,***b***Sphaerosyllis taylori*. A1-A4, B1-B4: Maximum intensity z-projections. **a1, b1**: Dorsal planes of α-tubulin-lir (grey). Diffuse nervous plexus of epidermis, strongest above parapodia. Four segmental ring neurite bundles form dorsal commissures. The dorsal longitudinal neurite bundle connects IIr to III, IV, I and IIr, proceeding from dorsolateral to mediolateral. **a2, b2**: Ventral, α-tubulin-lir. Four segmental neurite bundles reach laterally, then turn upwards to form a dorsal commissure each. Segmental neurite bundle II (parapodial innervation), consists of several separate neurite bundles. Yellow arrowhead: Segmental neurite bundle II and III are connected by a fine neurite bundle. **a3, b3**: α-tubulin-lir and cell nuclei (magenta). The ventral ganglia in *Pr. marmarae* are thick and well-defined. The ganglia of *Sph. taylori* are hardly differentiated. In the posterior part of the ganglion two commissures cross (white arrowhead in insets). **a4, b4**: Serotonin-lir of ventral planes. White arrows: the fourth segmental neurite bundle does not exhibit a serotonin-lir signal. Except for segmental neurite bundle IV all segmental neurite bundles and interconnections can be observed in the serotonin-lir of *Pr. marmarae*. The signal of segmental neurite bundles is very weak in *Sph. taylori*. **a5, b5:** Schematic drawing of the innervation of two segments. Green – neurite bundles, magenta – somata of the ventral nerve cord. **a5**: Several ventral papilla are innervated by segmental ring neurite bundles I and III or by additional segmental neurite bundles [1, 2]. The parapodial innervation consists of three separate bundles II.I-II.III leaving the ventral nerve cord. These are connected to each other (*). Segmental neurite bundles are linked by a ventral and lateral longitudinal neurite bundle. **b5**: Short neurite bundles innervate ventral papillae. The parapodial innervation II consists of four separate neurite bundles II. I – II.IV. **Scale bars: Overview = 100** μm**, insets = 25 μm**. **Abbreviations:** dc – dorsal cirrus; dep – dorsal epidermal plexus; dln – dorsal longitudinal neurite bundle; int – intestine; mn – median ventral nerve; mvn – main ventral nerve; n - nephridium; pc – parapodial cluster of somata; pg – parapodial gland of *Sph. taylori*; pk – serotonin-lir perikarya; ppl – parapodial lobe; sp. – sensory papilla; vc – ventral cirrus; vgl – ganglion of ventral nerve cord; vln – ventral longitudinal neurite bundle; 1 – first fine segmental neurite bundle; 2 – second fine segmental neurite bundle. **Segmental neurite bundles in yellow**: Isrl – small root of segmental ring neurite bundle I; IIIsrl – small root of segmental ring neurite bundle III; I-IV – segmental neurite bundles forming ring commissures, all neurite bundles leaving the vnc to form the parapodial innervation are denoted by II; IIr - dorsal commissure of root 2 of II

**Fig. 19:**
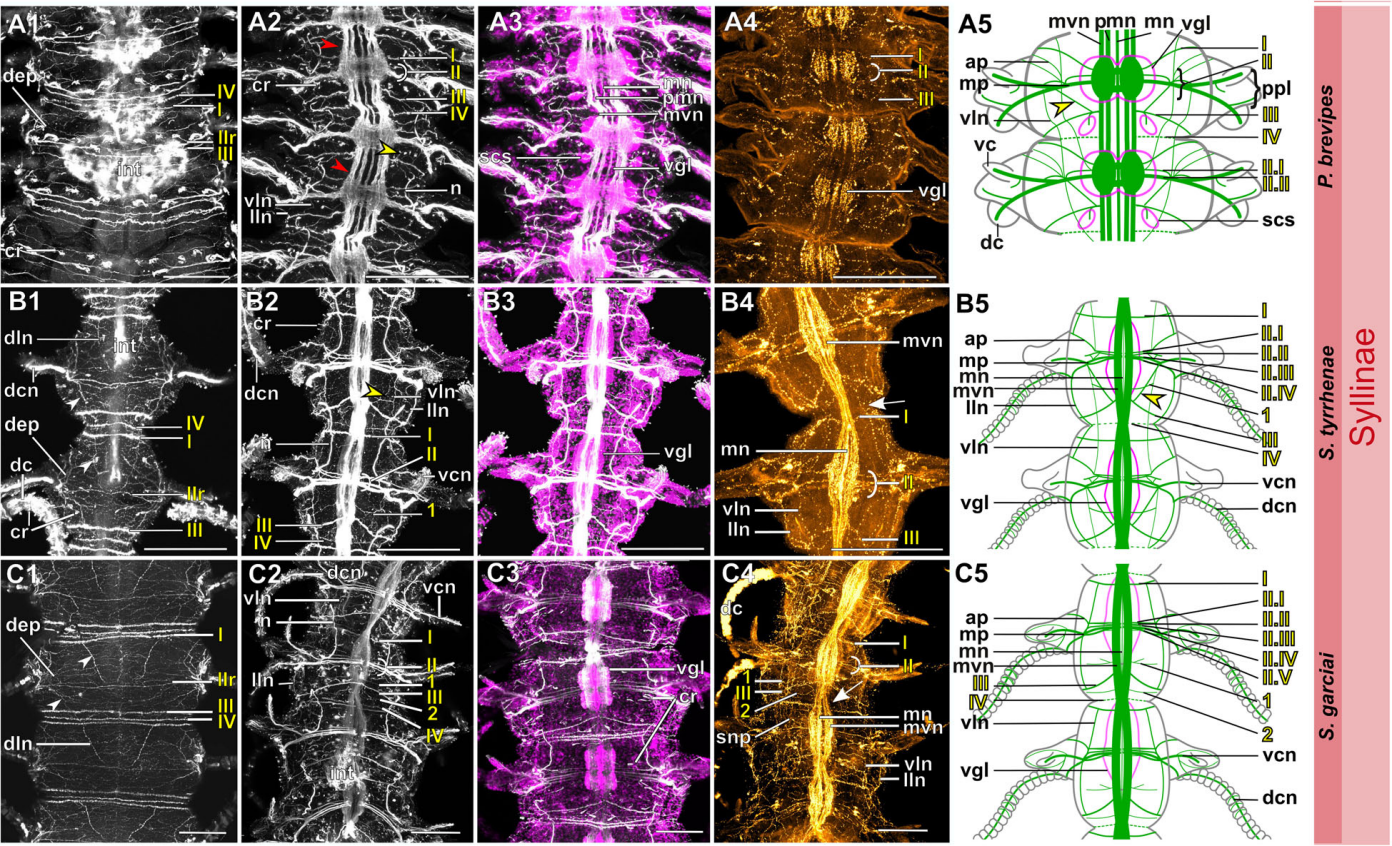
Syllinae. Segmental innervation. **a***Plakosyllis brevipes*, **b***Syllis tyrrhena*, **c***Syllis garciai*. A1-A4, B1-B4, C1-C4: Maximum intensity z-projections. **a1, b1, c1**: α-tubulin-lir of dorsal planes. Diffuse nervous plexus of the epidermis. Four segmental ring neurite bundles form dorsal commissures. The dorsal longitudinal neurite bundle connects IIr to III, IV, I and IIr, proceeding from dorsolateral to mediolateral. **a2, b2, c2**: Ventral planes of α-tubulin-lir. *P. brevipes:* five connectives are present and three ventral nerves within the ganglion. *Syllis* species: only the two main nerves are visible. Yellow arrowhead: a fine neurite bundle connects to II and III. Adjoining segments are connected by a ventral and a lateral longitudinal neurite bundle. *S. tyrrhena* possesses one and *S. garciai* two additional fine segmental neurite bundles. The intersegmental neurite bundle IV is missing between some segments of *P. brevipes* (red arrowheads). **a3, b3, c3**: α-tubulin and cell nuclei (magenta). The ventral ganglia are situated slightly closer to the anterior edge of the segment. A cluster of somata accompanies segmental neurite bundle III in *P. brevipes*. **a4, b4, c4**: Serotonin-lir of ventral planes. White arrows: no Segmental neurite bundle IV. An elaborate serotonin-lir epidermal nervous plexus is visible*.***a5, b5, c5**: Schematic drawing of the innervation of two segments. Green – neurite bundles, margenta – somata of the ventral nerve cord. **a5**: *Plakosyllis brevipes*. No additional segmental neurite bundles are present. The ventral longitudinal neurite bundle traverses over three segments. Segmental neurite bundle III sends fibres into a ventral cluster of somata. The parapodial innervation II consists of two neurite bundles. **b5**: *Syllis tyrrhena*. One additional segmental neurite bundle is present. The parapodial innervation II consists of four separate neurite bundles II.I.-II.IV. **c5**: *Syllis garciai*. Two additional segmental neurite bundles innervating the nervous plexus are present. **Scale bars = 100** μm**. Abbreviations:** ap – anterior neurite bundle entering the parapodium; cr – ciliary receptor; dc – dorsal cirrus; dcn – neurite bundle innervating dorsal cirrus; dep – dorsal epidermal plexus; dln – dorsal longitudinal neurite bundle; int – intestine; lln – lateral longitudinal neurite bundle; pmn – paramedian ventral nerve; ppl – parapodial lobe; mn – median ventral nerve; mp – main parapodial neurite bundle entering parapodium; mvn – main ventral nerve; n - nephridium; scs – segmental cluster of somata on segmental neurite bundle III; snp – serotonin-lir epidermal nervous plexus; vc – ventral cirrus; vcn – neurite bundle innervating ventral cirrus; vgl – ganglion of ventral nerve cord; vln – ventral longitudinal neurite bundle. **Segmental neurite bundles in yellow**: 1 – first fine segmental neurite bundle; 2 – second fine segmental neurite bundle; I-IV – segmental neurite bundles forming ring commissures; II.I-II.V – neurite bundles comprising parapodial innervation; II.IIr – dorsal commissure of root 2 of II

**Fig. 20:**
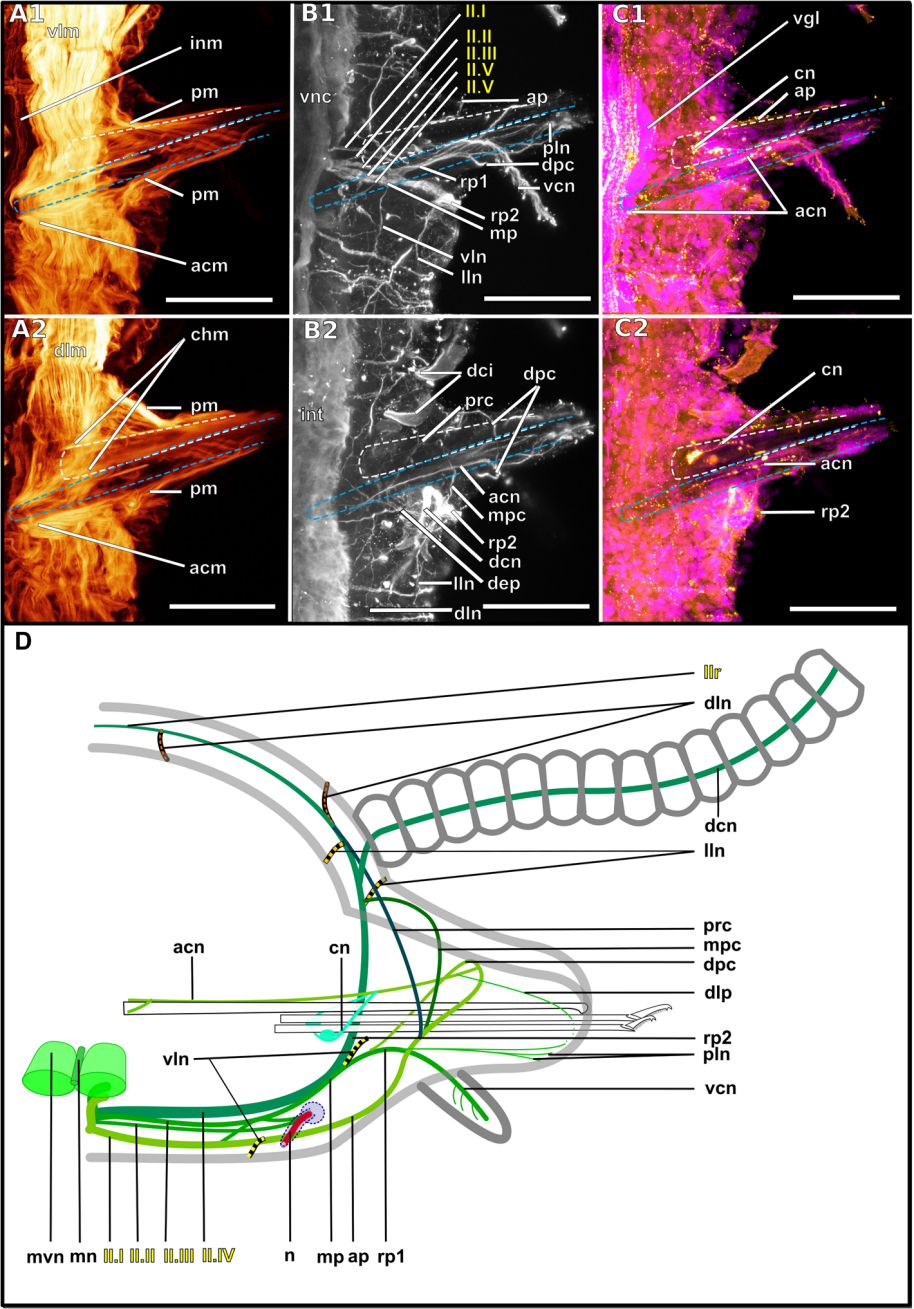
Parapodial innervation on the example of *Syllis garciai* and *Syllis tyrrhena*. **a1-c2**: Maximum intensity projections of *S. garciai*. A1, B1, C1 are projections of ventral, A2, B2, C2 are projections of dorsal planes. Blue dotted lines indicate the position of the acicle, white dotted lines the position of the chaetae. **a1, a2**: F-actin staining showing the parapodial muscles, acicular muscles and chaetal muscles. **b1, b2:** α-tubulin-lir showing the individual neurite bundles forming the parapodial innervation. **c1, c2**: Serotonin-lir. Mainly the innervation of the acicular muscle and of the bristles is stained. **d**: Schema of the innervation of the parapodium in *S. tyrrhena*, applicable for all investigated species. View from anterior. The parapodial innervation consists of several fibre bundles. The most anterior one and the most posterior one are connected to the ventral longitudinal neurite bundle. These neurite bundles then merge to two separate bundles, the anterior parapodial neurite bundle and the thicker main parapodial neurite bundle. The main bundle splits into two roots (rp1, rp2). Fibres of rp1 reach dorsally along the parapodial plane, form a distal dorsal connective with the anterior neurite bundle on the dorsal side of the parapodium and innervate the ventral cirrus and the parapodial lobe. A neurite bundle turns back from the parapodial lobe towards the distal parapodial connective, joining the neurite bundle innervating the acicular muscles. Serotonin-lir fibres connect to the acn and form a loop around the chaeta. Fibres of root 2 reach dorsally, form a median parapodial connective to the anterior parapodial neurite bundle, innervate the dorsal cirrus, form a proximal parapodial connective to the anterior parapodial neurite bundle and form the dorsal ring commissure IIr. From this root the nervous plexus above the parapodium is formed, too. **Scale bars = 50** μm**. Abbreviations:** acm – acicular muscles; acn – neurite bundle accompanying acicle; ap – anterior neurite bundle entering the parapodium; chm – chaetal muscles; cn – neurite bundle accompanying chaetae; dci – inclusions of dorsal cirrus in *S. garciai*; dcn – neurite bundle innervating dorsal cirrus; dep – dorsal epidermal plexus; dlm – dorsal longitudinal muscle; dln – dorsal longitudinal neurite bundle; dlp – distal innervation of the parapodial lobe; dpc – distal dorsal parapodial connective; inm – muscles of the intestine; int – intestine; lln – lateral longitudinal neurite bundle; mn – median ventral nerve; mp – main paraodial neurite bundle entering parapodium; mpc – median dorsal parapodial connective; mvn – main ventral nerve; n – nephridium; pln – neurite bundles innervating parapodial lobe; pm – parapodial muscle; prc – proximal dorsal parapodial connective; rp1 – root 1 of main parapodial neurite bundle; rp2 – root 2 of main parapodial neurite bundle; vcn – neurite bundle innervating ventral cirrus; vgl – ganglion of ventral neurite bundle cord; vlm – ventral longitudinal muscle; vln – ventral longitudinal neurite bundle; vnc – ventral nerve cord. **Segmental neurite bundles in yellow**: I-IV – segmental neurite bundles forming ring commissures; II.I-II.V – neurite bundles comprising parapodial innervation; IIr - dorsal commissure of root 2 of II

**Fig. 21:**
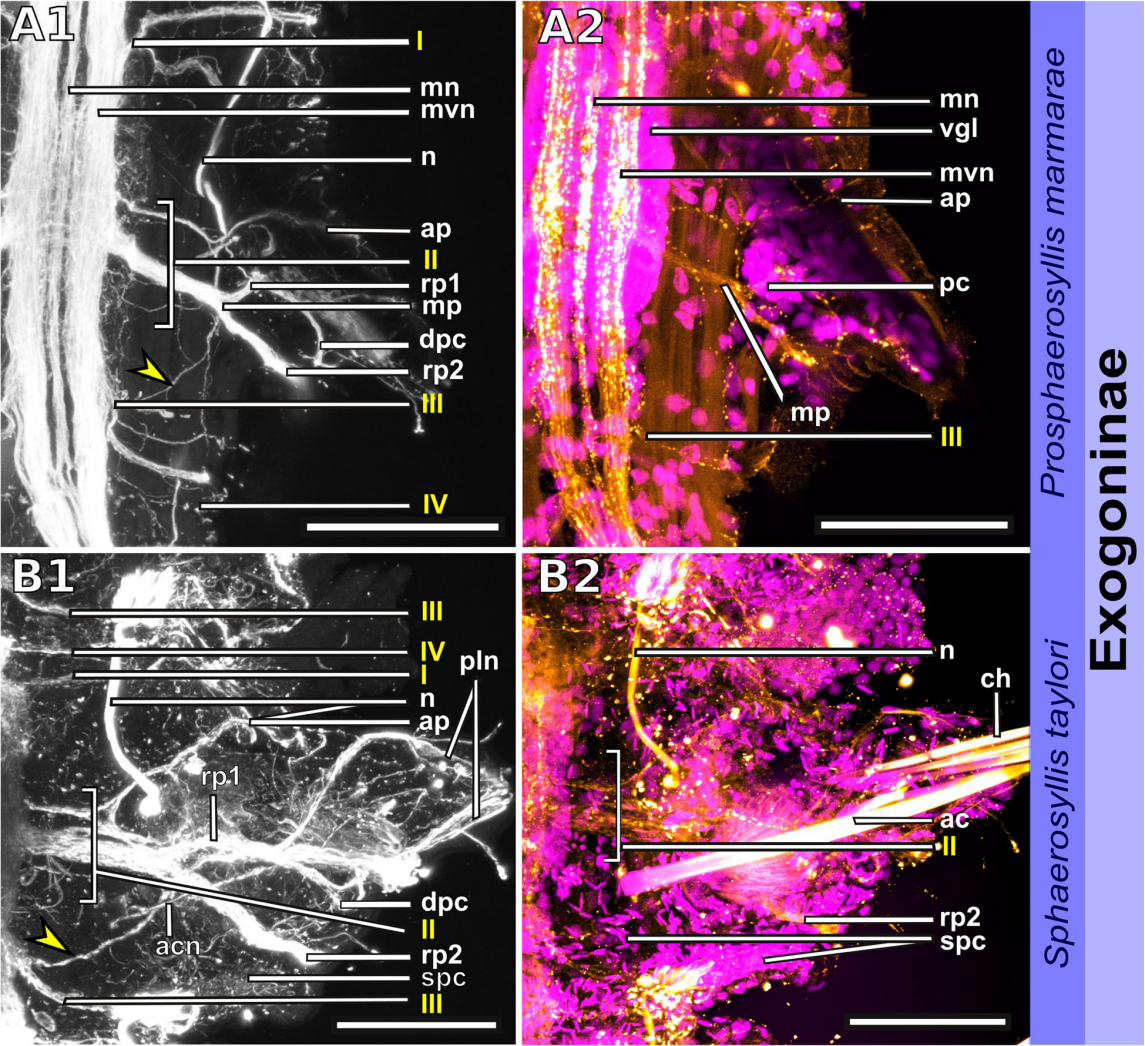
Exogoninae. Parapodial innervation. Maximum intensity z-projection of ventral sections. **a1:** α-tubulin-lir (grey) of a parapodium of *Prosphaerosyllis marmarae.* Three neurite bundles leave the ventral nerve cord to innervate the parapodium. The main parapodial neurite bundle is connected to the third segmental ring neurite bundle (yellow arrowhead). **a2**: Serotonin-lir (orange) and cell nuclei (magenta) of a parapodium of *Pr. marmarae*. Serotonin-lir of the main parapodial neurite bundle is less conspicuous and not all of the fine neurite bundles are visible, such as the most posterior fine neurite bundle of the parapodial innervation. A cluster of somata is present inside the parapodia. **b1**: α-tubulin-lir (grey) of a parapodium of *Sphaerosyllis taylori*. Four neurite bundles form the parapodial innervation. The main parapodial neurite bundle is connected to the third segmental ring neurite bundle (yellow arrowhead). **b2**: Serotonin-lir (orange) and cell nuclei (magenta) of a parapodium of *Sph. taylori*. The staining of the parapodial neurite bundles is weaker than in α-tubulin-lir, the most posterior fine neurite bundle is not stained. The nephridium is accompanied by a serotonin-lir signal in sexually active individuals. The chaetae have a strong autofluorescence at 568 nm in this species. **Scale bars = 50** μm**. Abbreviations:** ac – acicle; acn – neurite bundle accompanying acicle; ap – anterior neurite bundle entering the parapodium; ch – chaetae; dpc – neurite bundle forming distal dorsal parapodial connective; mp – main parapodial neurite bundle; mpc – median dorsal parapodial connective; mn – median ventral nerve; mvn – main ventral nerve; n - nephridium; pc – parapodial cluster of somata; pln – neurite bundles innervating parapodial lobe; rp1 – root 1 of main parapodial neurite bundle; rp2 – root 2 of main parapodial neurite bundle; spc – tubuli of spermatocytes; vgl – ganglion of ventral nerve cord. **Segmental neurite bundles in yellow**: I-IV – segmental neurite bundles forming ring commissures, II refers to all neurite bundles comprising the parapodial innervation

**Fig. 22:**
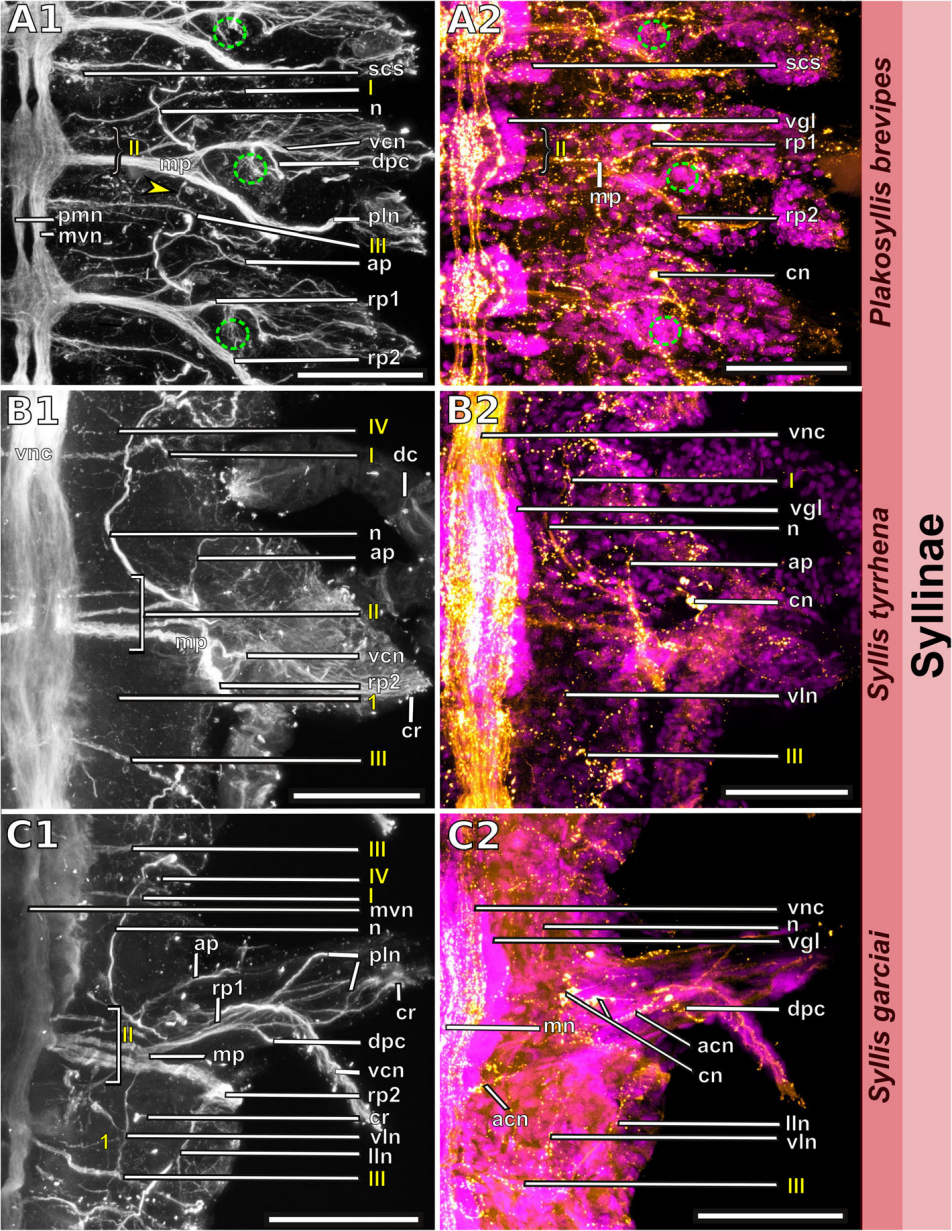
Syllinae. Parapodial innervation. Maximum intensity z-projection of ventral sections. **a1**: α-tubulin-lir (grey) of a parapodium of *Plakosyllis brevipes.* Three neurite bundles leave the ventral nerve cord and innervate the parapodium. A cluster of somata (green dotted line) is innervated by a small neurite bundle coming from root 1 of the main parapodial neurite bundle. The main parapodial neurite bundle and the third segmental ring neurite bundle are connected (yellow arrowhead). **a2**: Serotonin-lir (orange) and cell nuclei (magenta) of the same parapodium of *P. brevipes* as in A1. The serotonin-lir signal is not as defined as the α-tubulin-lir but nearly all neurite bundles of the parapodial innervation are stained. Only the signal of the innervation of the ventral cirrus appears much weaker. **b1**: α-tubulin-lir (grey) of a parapodium of *Syllis tyrrhena*. Four neurite bundles form the parapodial innervation. **b2**: Serotonin-lir (orange) and cell nuclei (magenta) of the same parapodium of *S. tyrrhena* as in B1. The signal of the parapodial neurite bundles is restricted to the most anterior and the main neurite bundle. (C1 and C2 are the same as in B1 and B2 in Fig. 19, but are shown here again for a better overview.) **c1**: α-tubulin-lir (grey) of a parapodium of *Syllis garciai*. Five neurite bundles form the parapodial innervation. **c2**: Serotonin-lir (orange) and cell nuclei (magenta) of the same parapodium of *S. garciai* as in C1. The parapodial innervation is hardly stained. Staining results of serotonin-lir differed greatly among individuals. **Scale bars = 50 μm. Abbreviations:** acn – neurite bundle accompanying the acicle; ap – anterior neurite bundle entering the parapodium; cn – neurite bundle accompanying chaetae; cr – ciliary receptor; dc – dorsal cirrus; dpc – neurite bundles forming distal dorsal parapodial connective; lln – lateral longitudinal neurite bundle; mn – median ventral nerve; mp – main parapodial neurite bundle entering parapodium; mvn – main ventral nerve; n - nephridium; pln – neurite bundles innervating parapodial lobe; pmn – paramedian ventral neurite bundle; rp1- root 1 of main parapodial neurite bundle; rp2 – root 2 of main parapodial neurite bundle; scs – segmental cluster of somata on segmental neurite bundle III; vcn – neurite bundle innervating ventral cirrus; vgl – ganglion of ventral nerve cord; vln – ventral longitudinal neurite bundle; vnc – ventral nerve cord. **Segmental neurite bundles in yellow**: 1 – first fine segmental neurite bundle; I-IV – segmental neurite bundles forming ring commissures, II refers to all neurite bundles comprising the parapodial innervation

Excepting *Plakosyllis brevipes,* all species have a ventral nerve cord comprised of three ventral nerves, a pair of main nerves and an unpaired median nerve (Figs. 17a1-e2, 18a2-a5, b2-b5, 19a2-a5, b2-b5, c2-c5, suppl. Fig. S2d, S3c, other data not shown). The ventral nerves fuse to certain degrees, but three separate ones are at least observable in some parts of the ventral nerve cord. The median nerve in the *Syllis* species is often only visible in the ganglion, while it is hardly distinguishable in the connectives (Figs. 17d1-e2, 19b2-b5, c2-c5). In α-tubulin-lir the median nerve can be obscured by the strong signal of the lateral nerves, but in serotonin-lir all three ventral nerves are distinguishable (Fig. 19b4, c4). In *Syllis garciai* the three connectives appear more or less fused in semi-thin sections of the anterior end (Fig. 17e1, e2).

In *Prosphaerosyllis marmarae* the median nerve is visible both in α-tubulin-lir and serotonin-lir, and also in semi-thin sections (Figs. 17a1, a2). It only fuses with the main nerves in parts of the ganglion. In *Sphaerosyllis taylori* on the other hand it is only distinguishable in a very small part of the ganglion in α-tubulin-lir and serotonin-lir (Fig. 18b2-b5). However, in this species the three ventral nerves are distinct in semi-thin sections of the ganglion (Fig. 17b1, b2). Posteriorly in the ganglion of the Exogoninae two small neurite bundles form diagonally crossing commissures of both main ventral nerves (Fig. 18a3 inset, b3 inset). These crossing commissures were not observed in the other species, but may be obscured by the strong signal of the ventral nerve cord.

Three connectives are visible in immunocytochemical stainings of *Prosphaerosyllis marmarae*, but in some regions fine gaps can be observed between them (Fig. 18a2). In anterior segments only three ventral nerves are present. In posterior segments the connectives of the main nerves are separated into various distinct bundles in both Exogoninae, clearly visible in histological semi-thin sections past the ganglion (Fig. 17a2, b2). The bundles lie adjacent to each other. They are only separated for a length of 10-20 μm and it is difficult to clearly distinguish them. The bundles fuse again before entering the next ganglion.

In contrast to the other four species the ventral nerve cord possesses five connectives in *Plakosyllis brevipes* (Figs. 17c2 inset, 19a2-a5) which partly fuse to three inside the ganglia (Figs. 17c1, 19a2-a5). These five connectives appear from the second chaetigerous segment onward, in the first segment only three connectives are present.

While all species have a high number of serotonin-lir perikarya in the ventral nerve cord (e.g. > 20 in *Sphaerosyllis taylori*, again an exact number is difficult to establish as some perikarya lie very close together) the serotonin-lir of perikarya is very low in *Prosphaerosyllis marmarae*. Even in intense stainings, not more than 3–4 serotonin-lir perikarya associated to the ventral nerve cord were found per segment.

In the *Syllis* species and the Exogoninae no distinct commissures of the ventral nerve cord were observed. Yet, the nerves partly fuse within the ganglia. Commissures within the ganglion were observed in *Plakosyllis brevipes*, but it is not possible to resolve if there are 3, 4 or 5 commissures (Fig. 19a2).

In all species the segmental ganglia are slightly shifted anteriorly with regard to the centre of the segment (Figs. 18, 19), but never traverse into the previous segment. Only in the first two segments, the tentacular segment and the first chaetiger, the ganglia of both segments are fused and, therefore, extend across the segmental boundary (Figs. 13, 14). In *Sphaerosyllis taylori* the ganglia are hardly separated from each other. The somata of the ganglia almost touch each other only leaving rather short and indistinct connectives, giving the ventral nerve cord an almost medullary appearance (Fig. 18b3, b5). In the other species there are noticeably fewer or more condensed somata accompanying the connectives.

In *Shaerosyllis taylori* additional clusters of somata form dorsoposterior extensions of the ganglia (Figs. 4b, e, 13). In the third segment the dorsoposterior ganglions are hardly visibly anymore and in the fourth segment they are absent. These extra anterior ventral ganglia are not found in any of the other species and may be a result of a small body size.

### Segmental neurite bundles and ring neurite bundles of the achaetous segment

In the achaetous segment three neurite bundles emerge from the central nervous system. The first is the strongest and enters the tentacular cirri followed by two smaller ones situated at the posterior edge of the achaetous segment (Figs. 3f, 5e, 9b, g). The neurite bundle innervating the tentacular cirri and the following one come off from the circumoesophageal connective, the last one originates from the region where the circumoesophageal connectives fuse to form the ventral nerve cord. It is not clear if the second ring neurite bundle lies within the first chaetigerous segment forming the first segmental neurite bundle of chaetiger 1 or if it lies in front of the segment boundary and thus belongs to the achaetous segment. Dissepiments are very likely reduced in the anterior segments enabling movement of the pharyngeal complex and complicate a clear distinction between segments.

**Fig. 23:**
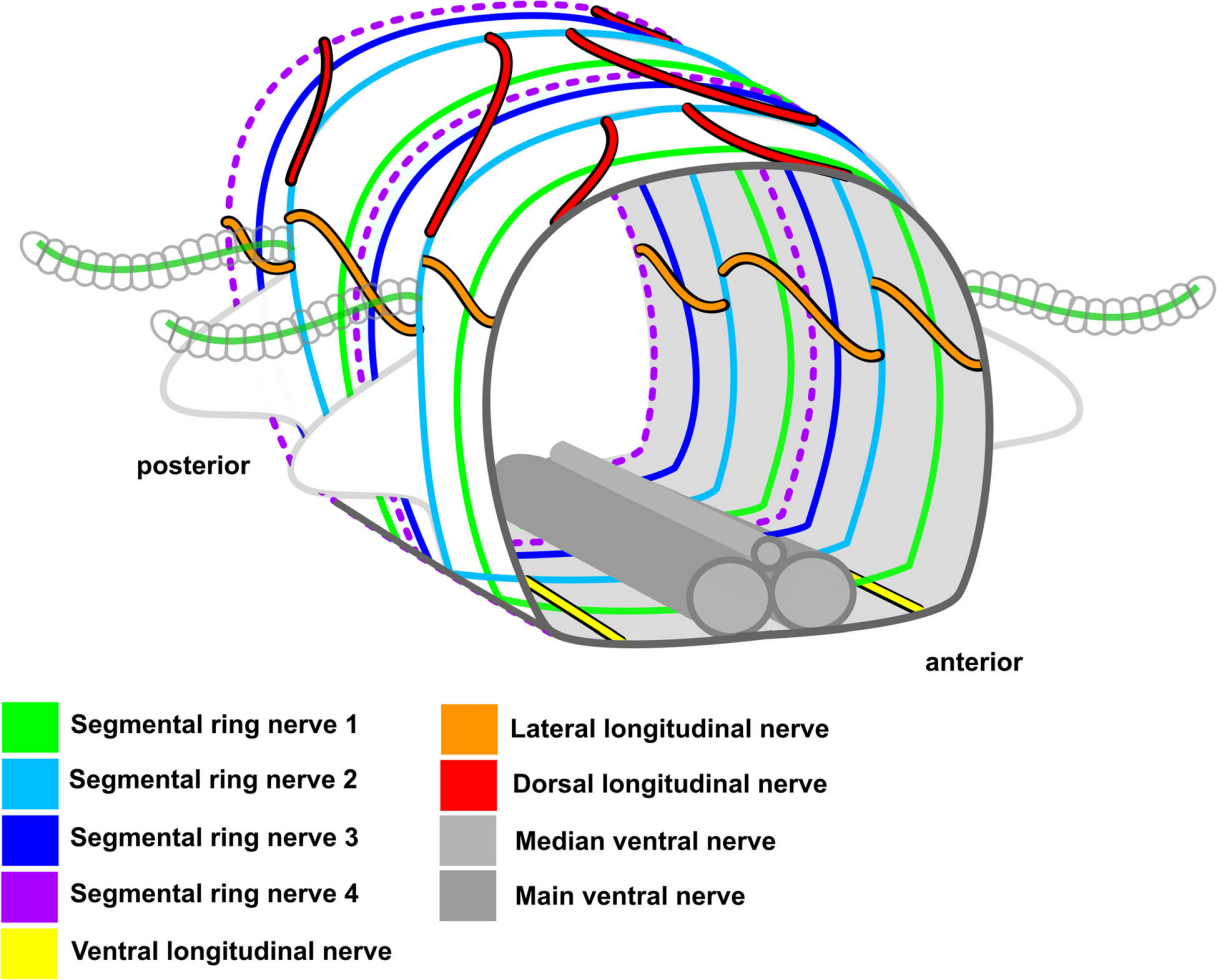
Schema of the longitudinal innervation of a segment in Syllidae. Four segmental ring neurite bundles are present per segment, of which the fourth one lies at segment boundaries and may be missing between some segments. The dorsal longitudinal neurite bundle connects segmental neurite bundle II of one segment to III, IV of the same and I and II of the next segment. It starts above the neurite bundle innervating the ventral cirrus on segmental neurite bundle II and ends in a dorsomedian position on the next segmental neurite bundle. The lateral longitudinal neurite bundle emerges underneath the neurite bundle innervating the tentacular cirrus and ends above it, connecting II to III, IV, I and II. The ventral longitudinal neurite bundle follows a straight course, interrupted by the neurite bundles forming the parapodial innervation (II), except for *P. brevipes*. The longitudinal neurite bundles follow a straight course in anterior segments approximately until reaching the proventricle. They then follow the course of the schema

The segmental innervation is highly variable among species. At least three segmental ring neurite bundles are always present. A fourth intersegmental ring neurite bundle can be missing in irregular patterns in anterior segments of *Syllis garciai* and *Syllis tyrrhena* and throughout the whole body of *Plakosyllis brevipes* and *Myrianida* and was not observed in the Anoplosyllinae (Figs. 18, 19, 23, suppl. Figs. S2d, S3c). It is only visible in α -tubulin-lir stainings. In *Plakosyllis brevipes* and *Prosphaerosyllis marmarae* the first segmental (ring) neurite bundle emerges within the ganglion, in the *Syllis* species it emerges shortly anterior of the ganglion (Figs. 18, 19). The dorsal commissure of the second ring neurite bundle is formed by a dorsal branch of root 2 of the strongest parapodial neurite bundle. It is difficult to distinguish between dorsal plexus and segmental neurite bundles; neurite bundles of the nervous plexus above the parapodium emerging from the parapodial neurite bundle may sometimes form an additional dorsal commissure (Fig. 18a1, b1).

### Parapodial innervation and transition from uni- to bilobed parapodia

The parapodial innervation is similar in all five species and *Streptosyllis websteri* (suppl. Fig. S2e). The only notable difference among species is the number of neurite bundles that comprise the parapodial innervation (Figs. 18a5, b5, 19a5, b5, c5, 20b1, d, 21, 22). The number ranges from five in *Syllis garciai*, four in *Syllis tyrrhena* and *Sphaerosyllis taylori*, three in *Prosphaerosyllis marmarae* to two in *Plakosyllis brevipes* (Figs. 21, 22). In some specimens of *Streptosyllis websteri* it seems that the parapodial neurite bundles consisted of two, while in others it consisted of three bundles, probably depending on the intensity of the staining (suppl. Fig. S2d, e). All of these neurite bundles are connected to each other. The fine bundles reaching from the ventral nerve cord to the base of the parapodium are termed II.I-II.V. The labels are supposed to illustrate the number of neurite bundles comprising the parapodial innervation. It should be noted that these bundles are only clearly distinguishable in α-tubulin-lir stainings. In serotonin-lir stainings the anteriormost bundle is clearly stained, while only a weak signal can be observed from the other bundles, until they reach the parapodial base (Figs. 21, 22). Once the neurite bundles reach the parapodial base two neurite bundles continue, the main parapodial neurite bundle and the anterior neurite bundle (Fig. 20d). The main neurite bundle splits into two roots (Figs 20b1, d, 21a1, b1, 22a1, b1, c1). Root 1 innervates the ventral cirrus and root 2 innervates the dorsal cirrus, which innervates at least a pair of ciliated cells per annulus (Fig. 19 b2, c2, 20d). Both roots also form several distinct bundles innervating the parapodial musculature.

Root 1 forms a dorsal arch and connects to the anterior parapodial neurite bundle forming the distal parapodial connective (Fig. 20d). From this connective a long neurite bundle is sent along the acicula, which bifurcates before innervating the acicular musculature. It sends neurite bundles to the chaetae, forming a loop around them and re-connects to the acicular neurite bundle (Figs. 20b2, c1, c2, d, 22c2). This bundle turns backwards before reaching the base of the chaeta. The innervation of the acicular muscles can be observed in α-tubulin-lir, but is especially strong in serotonin-lir (Fig. 20b1, b2, b1, c2).

Other neurite bundles of root 1 reach the most distal plane of the parapodium on its ventral side. One of them turns back along the dorsal plane of the parapodium (Fig. 20d) (a direct connection could not be observed in all species) and joins the neurite bundle innervating the acicular muscles. Root 2 runs dorsally on the posterior side of the parapodium (Fig. 20d). It divides into three neurite bundles; the first forms the median parapodial connective to the anterior parapodial neurite bundle. The second innervates the dorsal cirrus. The third forms the most proximal dorsal parapodial connective to the anterior parapodial neurite bundle and a dorsal commissure to root 2 of the parapodium on the other side of the segment (segmental ring neurite bundle) (Fig. 20b2, d). Additional fine neurite bundles connect to the nervous plexus of the body wall and may form a second commissure. Slight variations could be found in the parapodial innervation between species. In *Pr. marmarae* parapodial papillae are innervated by additional neurite bundles, and a cluster of cell nuclei at the beginning of root 2 (Fig. 21a2) may be associated with a parapodial gland in this species (Fig. 17a1). *Sphaerosyllis taylori* has conspicuous glands filled with fibrous material situated dorsally to the acicle inside the parapodium (enclosed by the acicular muscles, not shown) (Fig. 17b1, 18b3), which are species specific and used as a character for identification and innervated from the proximal parapodial connective.

**Fig. 24:**
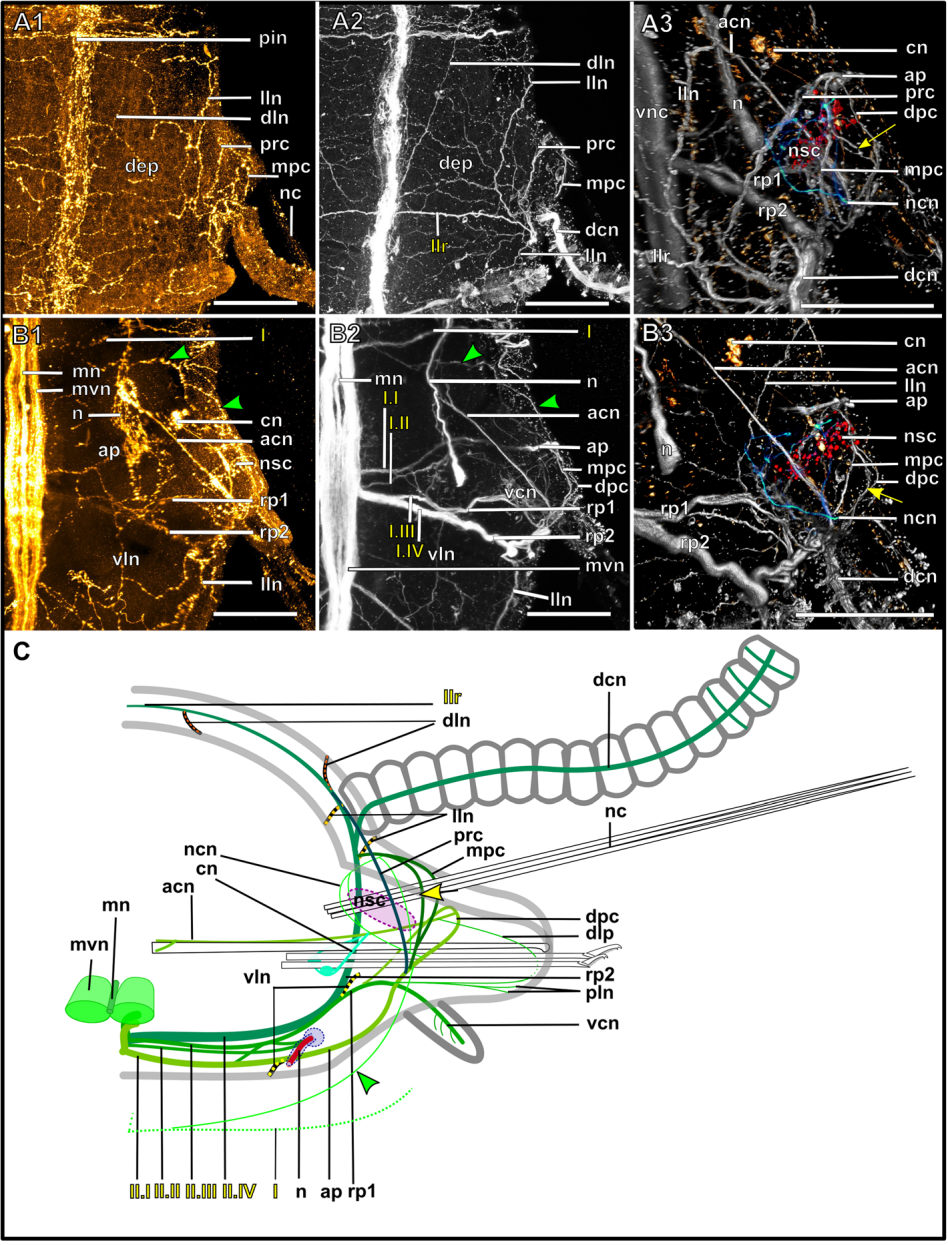
S*yllis tyrrhena.* Parapodial innervation of stolons. **a1, a2**: Maximum-intensity z-projections of dorsal sections. **a1**: Serotonin-lir. The dorsal side of the segment is innervated by an epidermal plexus emerging from root 2 of the parapodial innervation. **a2**: α-tubulin-lir. Similar to serotonin-lir staining, some of the neurite bundles forming the epidermal less are less prominent. **a3**: Volume rendering of the parapodium, dorsolateral view. α-tubulin-lir (grey), serotonin-lir (orange). α-tubulin-lir neurite bundles innervating the notopodial swimming chaeta are segmented in green, serotonin-lir signal accompanying the notopodial chaeta are segmented in red. All four dorsal connectives of the parapodium are visible. Yellow arrow: fourth dorsal commissure present only in parapodia of stolons of *S. tyrrhena*. **b1, b2**: Maximum-intensity z-projections of ventral sections. **b1**: Serotonin-lir. The nephridium is accompanied by a strong serotonin-lir signal. A neurite bundle (green arrowheads) connects the anterior parapodial neurite bundle to segmental neurite bundle I and is only found in the stolons. **b2**: α-tubulin-lir showing the course of the second segmental nerve towards the parapodium. **b3**: Volume rendering of the parapodium, ventral view. α-tubulin-lir (grey), serotonin-lir (orange). α-tubulin-lir neurite bundles innervating the notopodial swimming chaeta are segmented in green, serotonin-lir signal accompanying the notopodial chaeta are segmented in red. **c**: Schema of the parapodium of the stolon. The innervation is essentially the same as in the stock animals but for a few additions; an additional dorsal connective (yellow arrow) forms from the median parapodial connective, enveloping the notopodial chaetae. The chaetae are innervated by fine α-tubulin-lir fibres and a strong serotonin-lir signal surrounds the notopodial chaeta. A neurite bundle connects the anterior parapodial neurite bundle to segmental ring neurite bundle I. **Scale bars = 50 μm. Abbreviations:** ap – anterior neurite bundle entering the parapodium; acn – neurite bundle inaccompanying acicle; cn – neurite bundle accompanying chaetae; dcn – neurite bundle innervating dorsal cirrus; dep – dorsal epidermal plexus; dln – dorsal longitudinal neurite bundle; dlp – distal innervation of the parapodial lobe; dpc – distal dorsal parapodial connective; lln – lateral longitudinal neurite bundle; mn – median ventral nerve; mpc – median dorsal parapodial connective; mvn – main ventral nerve; n – nephridium; nc – notochaetae; ncn – α-tubulin-lir neurite bundle innervating notochaetae; nsc – serotonin-lir signal accompanying notochaetae; pin – nervous plexus innervating intestine; pln – neurite bundles innervating parapodial lobe; prc – proximal dorsal parapodial connective; rp1 – root 1 of main parapodial neurite bundle; rp2 – root 2 of main parapodial neurite bundle; vcn – neurite bundle innervating ventral cirrus; vgl – ganglion of ventral nerve cord; vln – ventral longitudinal neurite bundle. **Segmental neurite bundles in yellow**: I – first segmental ring neurite bundle; II.I-II.V – neurite bundles comprising parapodial innervation; llr - dorsal commissure of root 2 of II

Stolons of *Syllis tyrrhena* develop natatory chaetae, which are long, capillary notopodial chaetae (Fig. 24f). A distinct notopodial lobe is not formed. The median dorsal connective of the parapodium splits into two neurite bundles (Fig. 24a3, b3, c). Between these neurite bundles the natatory chaetae extend from the parapodium (Fig. 24c). From the anterior split of the median connective, a diffuse serotonin-lir signal surrounds the chaetae (Fig. 24a3, b1, b3, c). In addition to the diffuse signal, a very fine α-tubulin-lir neurite bundle forms a loop from the anterior region to the posterior region of the median connective (Fig. 24a3, b3, c). It also splits and forms two loops at its end.

A connection is formed between the anterior neurite bundle of the parapodial innervation and segmental neurite bundle I (Fig. 24b1, b2, c). The connection is not present in the stock animals. The only other notable difference in the segmental nervous system of the stolons is that the nephridium is accompanied by a diffuse serotonin-lir signal and serotonin-lir circle surrounds the nephridial porus (Fig. 24b1). The α-tubulin-lir of the nephridium is more diffuse than in the stock animal and neurite bundle II.II is longer than in the stock animals. It fuses with root 1 of the main parapodial neurite bundle and seems to innervate the serotonin-lir circle around the nephridial pore, but only shows α-tubulin-lir signal (Fig. 24b2).

### Peripheral innervation

With the exception of *Syllis garciai,* all of the more closely investigated species show a neurite bundle that connects the parapodial neurite bundle (II) to the following segmental ring neurite bundle (III) (Figs. 18a2, a5, b2, b5, 19a2, a5, b2, b5). *Syllis garciai* has an elaborate nervous plexus that connects several of the segmental neurite bundles (Fig. 19c2, c4). In addition to the four segmental ring neurite bundles, two additional segmental neurite bundles innervate the nervous plexus of the body wall and ventral ciliary receptors in *S. garciai* (Figs. 19c2, c5, 22c1). The first one leaves the ventral nerve cord between the parapodial neurite bundle (segmental ring neurite bundle II) and segmental ring neurite bundle III. A similar segmental neurite bundle can be found in *Syllis tyrrhena*, where it joins the ventral longitudinal neurite bundle (Figs. 19b2, b5, 22b1). The second additional segmental neurite bundle of *S. garciai* emerges between segmental ring neurite bundle III and IV. No segmental neurite bundle is present in *S. tyrrhena* in this region.

*Plakosyllis brevipes* does not possess any additional segmental neurite bundles. From segmental neurite bundle three a small neurite bundle leads to a distinct segmental cluster of somata not present in any of the other species (Figs. 19a3, a5, 22a1, a2).

Additional segmental neurite bundles can also be found in *Prosphaerosyllis marmarae,* which innervate ventral sensory papillae. In *Pr. marmarae,* both the first and third segmental ring neurite bundle branch after leaving the ventral nerve cord. One branch forms the dorsal commissure, while the other branch innervates a ventral papilla. Following the first segmental ring neurite bundle a small neurite bundle leaves the ventral nerve cord and innervates another ventral papilla. A little more posteriorly, a second small neurite bundle leaves the ventral nerve cord. It connects to the first segmental ring neurite bundle and also innervates a lateroventral sensory papilla. From the parapodial neurite bundle II.II another small neurite bundle reaches a ventral papilla (Fig. 18a2, a5, 21a1).

In *Sphaerosyllis taylori* no additional segmental neurite bundles are present (Figs. 18b2, b5, 21b1). The ventral papillae are innervated by the segmental ring neurite bundles; the first segmental ring neurite bundle forms a small branch which innervates the papillae, similar to *Prosphaerosyllis marmarae*, but much finer. Two pairs of ventral papillae are directly innervated by the parapodial neurite bundles. Two more pairs of papillae lie at the posterior edge of the segment. The median ones lie underneath the ventral nerve cord and are directly innervated by small fibre bundles from the ventral nerve cord. The more lateral ones are innervated by segmental ring nerve II. Between segmental nerve ring III and IV a small neurite bundle runs to the lateral margins of the segment, but it does not seem to arise from the ventral nerve cord.

### Longitudinal neurite bundles

All of the five species studied in more detail (also observed in *Eurysillis blomstrandi* and *Streptosyllis websteri*) have three pairs of longitudinal neurite bundles in addition to the ventral nerve cord; a pair of dorsal, a pair of lateral and a pair of ventrolateral longitudinal neurite bundles (Fig. 23). In anterior segments these neurite bundles are continuous and distinct (e.g. Fig. 16a). Approximately from the segment of the proventricle they become discontinuous, only connecting the segmental ring neurite bundles of adjacent segments (Figs. 18a1, b1, 19a1, b1, c1, 23). All longitudinal neurite bundles have the same origin; a fine neurite bundle leaves the circumoesophageal connective shortly after dorsal and ventral root fuse and anteriorly to the neurite bundle that innervates the tentacular cirri (Figs. 3f, 5e, 7c, 9b, g, 12). 

The neurite bundle that forms the longitudinal neurite bundles reaches towards the dorsal plane of the animal and bifurcates. It sends one neurite bundle to the dorsal side of the animal, which forms the dorsal longitudinal neurite bundle and receives fibres from the nuchal neurite bundle. A second neurite bundle reaches dorsolaterally and forms the lateral longitudinal neurite bundle. It receives fibres from the neurite bundles innervating the tentacular cirri (Fig. 12).

A small neurite bundle, which originates at the drcc, joins the neurite bundle forming the longitudinal neurite bundles in all species except *Prosphaerosyllis marmarae* and *Syllis tyrrhena.* Its fibres form at least part of the ventral longitudinal neurite bundle. It also sends fibres to the posterior inferior cluster of somata in *Sphaerosyllis taylori* (Fig. 12).

Both *Syllis* species have a dorsal ciliary band on the first achaetous segment and a discontinuous ciliary band on the first chaetigerous segment, which are both innervated by the dorsal longitudinal neurite bundles (Figs. 9e, 16a). In *Syllis tyrrhena* ciliary patches lying just above the dorsal cirri in the first three segments and the achaetous segment are innervated by the lateral longitudinal neurite bundles (Figs. 9e, 16a). *Plakosyllis brevipes* possesses patchy bands of cilia on all segments, but they are innervated by the second ring neurite bundle only. The innervation of dorsal ciliary bands in *Myrianida* is similar. The Exogoninae do not have any cilia on the segments.

In more posterior segments, the dorsal longitudinal neurite bundle branches off the dorsal commissure of segmental ring neurite bundle 2 close to the neurite bundle innervating the dorsal cirrus. It then connects the dorsal commissures of the following segmental neurite bundles, until it reaches the dorsal commissure of segmental ring neurite bundle 2 of the following segment in a dorsomedian position (Fig. 23). Its course is not strictly longitudinal, but starts from a lateral region of the body and ends in a dorsomedian region.

In anterior segments the lateral longitudinal neurite bundle lies dorsolaterally and forms a continuous lateral neurite bundle that connects all segmental ring neurite bundles within and across segments. In more posterior segments, the lateral longitudinal neurite bundle lies more laterally. It branches off the neurite bundle that also forms the second ring commissure and the innervation of the dorsal cirrus, connecting it to the following segmental ring neurite bundles. It reaches the second segmental ring neurite bundle of the adjoining segment in a slightly more dorsal position (Figs. 20b1, b2, d, 23). In *Syllis tyrrhena* the neurite bundle splits into very fine neurite bundles, which connects it to the nervous plexus of the body wall, ring neurite bundle 2 and the parapodial neurite bundle of root 1 of the main parapodial neurite bundle forming the median parapodial connective. In the other species only a connection to root 1 of the main parapodial neurite bundle could be observed.

The ventral longitudinal neurite bundle runs ventrolaterally, parallel to the ventral nerve cord. In more posterior segments it branches off root 1 of the main parapodial neurite bundle, shortly after root 1 and 2 separate. It then reaches to the following segmental ring neurite bundles and ends at the anterior neurite bundle of the parapodial innervation of the adjoining segment (Figs. 18a5, b5, 19b5, c5).

In *Plakosyllis brevipes* the ventral longitudinal neurite bundle connects three adjacent segments. It leaves root 1 of the main parapodial neurite bundle, passes through the next segment crossing the parapodial innervation again and then connects to the ganglion of the third following segment. Thus, two ventral longitudinal neurite bundles that run more or less parallel to each other are present in every segment (Fig. 19a5).

### Pygidial innervation

**Fig. 25:**
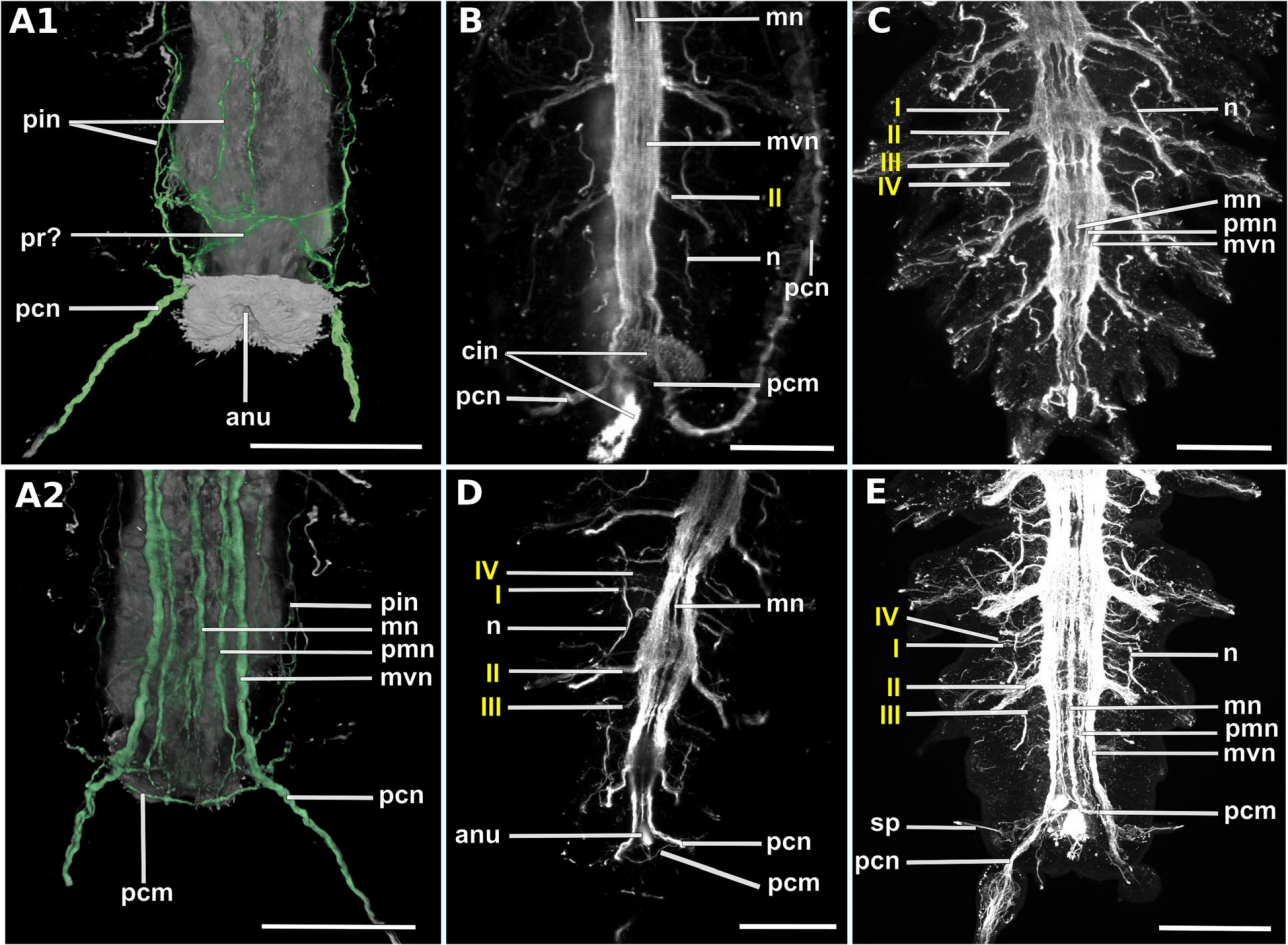
Pygidial innervation in Syllidae. **a1, a2**: *Syllis garciai***b**: *Syllis tyrrhena*. **c**: *Plakosyllis brevip*es. **d**: *Prosphaerosyllis marmarae*. **e**: *Sphaerosyllis taylori*. **a1**: Volume rendering of α-tubulin-lir, dorsal view. Innervation of the pygidium is segmented in green. The gut is innervated by a more differentiated nervous plexus than in anterior segments. **a2:** Ventral view. The ventral nerve cord drifts apart, separating into median, paramedian and main ventral nerves. The ventral nerves are connected by a pygidial commissure. From the main nerves a neurite bundle grows dorsally and innervates the pygidial cirri and possibly forms the pygidial ring commissure. **B**: *Syllis tyrrhena*. **c**: *Plakosyllis brevip*es. **d**: *Prosphaerosyllis marmarae*. **e**: *Sphaerosyllis taylori*. **b-e:** Maximum intensity z-projections of α-tubulin-lir, ventral sections. The innervation follows a similar pattern in all species. The ventral nerves only separate in *S. garciai* and *Sph. taylori*. **Scale bars = 50 μm**. **Abbreviations**: anu – anus; cin – cilia of intestine; mn – median ventral nerve; mvn – main ventral nerve; n – nephridium; pcm – pygidial commissure; pcn – neurite bundle innervating pygidial cirrus; pin – nervous plexus innervating intestine; pmn – paramedian ventral nerve; prn – pygidial ring neurite bundle; sp. – sensory papilla. **Segmental neurite bundles in yellow**: I-IV – segmental neurite bundles forming ring commissures

Once the nerves of the ventral nerve cord reach the pygidium they separate and spread out. In *Syllis garciai* and *Sphaerosyllis taylori* α-tubulin-lir and serotonin-lir stainings show that the main connectives separate into median and paramedian nerves (Fig. 25a2). At the end of the pygidium just underneath the anus, a commissure connects all nerves of the ventral nerve cord (Fig. 25, suppl. Fig. S2f). The main nerves of the ventral nerve cord reach dorsally and innervate the anal cirri (Fig. 25), which are present in most species. The *Syllis* species have an additional anal papilla. The source of its innervation was not observed. In *Syllis garciai* two very fine dorsal commissures, which connect to the neurite bundles coming from the main nerves and to the nervous plexus of the gut, are present (Fig. 25a1). The more posterior one may be the pygidial ring neurite bundle, but could also be part of the nervous plexus of the intestine. The neurites innervating the gut are better differentiated in the posteriormost segments (Fig. 25a1) than in anterior segments.

## Discussion

### Anterior innervation

The general appearance of the brain, its dorsal position and the innervation of anterior appendages is similar in all investigated species of the family Syllidae (suppl. Fig. S1). However, several small differences were found between species and are discussed in more detail. The innervation pattern of the antennae and tentacular cirri observed in this study is consistent with previous descriptions [32] (suppl. Fig. S4a), which depicted the antennal neurite bundles to be branches of the dcdr. This is very likely part of the ground pattern in all syllids. Clusters of somata (especially present in *Prosphaerosyllis marmarae*) were not described for Syllidae, but for example in Phyllodocidae, termed tentacular ganglia [53]. These clusters of somata probably consist of the somata of primary sensory cells of the ventral and dorsal tentacular cirri, and are dislocated and arranged in clusters due to limited space, like the dorsal lobes of the brain as discussed below. The resolution of the applied techniques is not high enough to link individual somata to their cell processes in the tentacular or dorsal cirri.

In total up to 12 palp nerve roots have been described across several species of annelids [25]. It is noteworthy to mention that no species exists possessing all of them. However, these palp nerve roots were used as an important character to homologise the anterior appendages across annelids. The number of palp nerves varies between families and sometimes within families, but so far few studies have compared the innervation of the palps within a family. In the sedentary Serpulidae and Sabellidae 8 palp nerve roots were found, in the errant families there are usually fewer [25]. Nerve root 6 and 9 are present in Syllidae, Nereididae (suppl. Fig. S4b), Hesionidae and all studied Aphroditiformia [25]. In other Phyllodocida such as Glyceriformia root 6 can be missing. In Syllidae palp nerve root 6 originates from the ventral commissure of the ventral root of the circumoesophageal connective (vcvr, [32]) and root 9 from the drcc, shortly before it splits into the dorsal and ventral commissure [25, 32] (suppl. Fig. S4a). The homologisation of these nerves from CLSM images is difficult, as their course is hard to observe if they originate within the neuropil and the brain commissures [32] are not always found [2]. However other authors were at least able to describe them and additional neurite bundles from reconstructions of serial TEM sections (*e.g.* [54]).

From the results of the present study it is clear that Syllidae have more than two nerve roots innervating the palps; a main bundle (pn1), a bundle originating from the drcc (pn2) and at least in some species one originating from the vrcc (pn3) (Fig. 12, suppl. Fig. S1). The neurite bundles forming the main palp neurite bundle (pn1) are likely homologous to nerve root 6. On the other hand, it is unclear which neurite bundles correspond to nerve root 9: Either the neurite bundles branching off the drcc after it has entered the neuropil of the brain, or pn2, which leaves the drcc before it enters the neuropil (see Fig. 12). Additional neurite bundles branching off the drcc that innervate the palps have been described in Nereididae and may be homologous to the fine neurite bundle (pn2) branching of the drcc in Syllidae, if it does not correspond to nerve root 9. Palp nerve root 11 branching off the drcc can be found both in Nereididae (suppl. Fig. S4b) and in Glyceridae within the Phyllodocida [25], but a homologisation with any nerve root found in the present investigation remains speculative and difficult when comparing data obtained by different methods.

Further neurite bundles branching off the vrcc (or its commissures) were so far only described in some Aphroditiformia within the Phyllodocida [25]. Even though they may be in a similar position, they were not described to innervate ciliary structures [55], thus a homologisation of pn3 in the Syllidae to any of the nerve roots of the vrcc in Aphroditiformia is impossible at present.

In *Autolytus*, *Syllis* and *Eurysyllis* a dorsal ganglion dorsally of the drcc has been described [32] (suppl. Fig. S4a). This dorsal cluster of somata [27, 33] is a common feature in several families including Hesionidae, Nereididae and other Phyllodocida such as Sigalionidae, Aphroditidae, Acoetidae, Polynoidae, Eunicidae, Glyceridae and Goniadidae [25, 27]. It is involved in the innervation of the palps in families possessing palp nerve 11 and is usually associated with the drcc [25]. It is assumed that the dorsolateral cluster of somata found in *Syllis tyrrhena* and *Syllis garciai* is homologous to the “dorsal ganglion” described in other species. This could also support the homology of pn2 in the Syllidae to palp nerve root 11 described in other families.

While no mushroom bodies were found in this study, an anterior and a lateroposterior mass of globuli cells were described in the syllid *Odontosyllis* cf. *fulgurans* (Eusyllinae) with immunocytochemical methods [10]. These clusters were interpreted as an indication for mushroom bodies, but are lacking a distinct neuropil, which should be present in true mushroom bodies [10]. Ganglia consisting of globuli cells were also described in the palps in the general reconstruction of the anterior nervous system of Syllidae from species of the genera *Syllis* (Syllinae), *Autolytus* (Autolytinae), *Eusyllis* (Eusyllinae) from paraffin sections [32]. Intrafamily variation regarding the presence and stage of clusters of globuli-like cells were found in Nephtyidae [56] and their presence could be similarly variable in Syllidae.

Prostomial sense organs at the laterofrontal margins of the brain are known from α-tubulin-lir stainings of *Neanthes arenaceodentata* (Nereididae) [11] (suppl. Fig. S4b). It was interpreted to be the anterior cephalic sense organ described in *Alitta (Nereis) virens* [57] and *Hediste (Nereis) diversicolor* [58], termed Langdons organ [11]. The Langdons organs have also been described in other annelids [57], but the species are not mentioned by the author. However, a closer comparison on a cellular level to the Langdons organ in *A. virens* would be necessary to confirm the homology of these structures in Syllidae and Nereididae.

The arrangement of serotonin-lir somata posterior to the brain was described and used by other authors [2] to compare different errant and sedentary annelid species and deduct a ground pattern for the Annelida. As shown by our investigations, at least in Syllidae, the number and staining affinities of such somata seems to be relatively variable and are thus not feasible for comparisons between species.

### Dorsal and lateral brain lobes and other clusters of somata

A striking difference between the morphology of the anterior nervous system in Exogoninae and other Syllidae is the presence of a pair of conspicuous dorsal lobes in the Exogoninae (see Figs. 13, 14 and suppl. Fig, S1). Posterior lobes of somata have been observed before in other Exogoninae; in *Parapionosyllis longicirrata* and in 9 species of *Sphaerosyllis.* They were termed dorsal and lateral lobes of the brain [59], while in *Sphaerosyllis brevifrons* highly pigmented lateral nuchal glands (lobes) were described [60]. In a detailed study on *Pionosyllis manca* [61] (Eusyllinae) (erroneously named as *Parapionosyllis manca* (Exogoninae) [62]), it was concluded that the dorsal and lateral lobes consist of supportive cells of the nuchal organ [62]. Fibres of the nuchal neurite bundle originating from the brain (equivalent to the posterior neurite bundle of the brain in this study) reach into the dorsal and lateral lobes of *P. manca*.

These lobes were considered to represent possible taxonomic characters of *Parapionosyllis* and *Sphaerosyllis* [62]. Since the dorsal lobes in the Exogoninae *Prosphaerosyllis marmarae* and *Sphaerosyllis taylori* consist of somata similar to the remaining somata of the brain, the dorsal lobes are real lobes of the brain. Moreover, posterior neurite bundles of the brain reach far into these somata and they are not separated from the remaining somata of the brain. Therefore, they are a possible apomorphy for the Exogoninae [62]. In *Exogone* no lobes of the brain were described [59], but histological sections or investigations via immunocytochemistry and CLSM are necessary to confirm this. Unfortunately it was not possible to obtain a proper nuclear staining for *Exogone*.

Dorsal lobes that consist of support cells of the nuchal organ, as found in *Pionosyllis manca,* probably correspond to the nuchal lobes present in all five in detail investigated species of Syllidae, and are not homologous to the dorsal lobes of the brain of the Exogoninae. The nuchal lobes also contain nervous fibres (at least α-tubulin-lir fibres) (Fig. 3h) as was observed in the dorsal and lateral lobes in *P. manca* [62].

Dorsal lobes of the brain are found in small-sized species of other families. *Fauveliopsis adriatica*, a sedentary annelid of Cirratuliformia [63], possesses two pairs of posterior lobes consisting of somata, which were linked to the ability to retract the prostomium in these animals [64, 65]. *Sphaerosyllis* and *Prosphaerosylli*s can also retract their prostomium, which may explain similarities in the morphology of the anterior nervous system, even though Syllidae and Fauveliopsidae are not closely related. Similar lobes were observed in other Fauveliopsidae [60, 66, 67]. In *F. adriatica* the nuchal organ is embedded in the lobes of the brain and somata of dorsal and lateral lobes cannot be distinguished [64, 65]. Four lobes extend posteriorly in the head segment of *Astomus taenioides* (as *Parnterodrilus taeniodes*), with the lateral ones fusing with the drcc [68]. Posterior lobes with varying sizes can also be found in several species of Nephtyidae, but these were described as prostomial mucus-glands [56, 69].

Posterior inferior clusters of somata, as present in both Exogoninae, possibly consist of somata from the sensory cells, like the so called nuchal ganglia of other annelid species [64, 70]. Such clusters of cells [27, 33] have been described in many annelids, e.g. in *Syllis*, *Eurysllis* and *Autolytus* (Syllidae) [32]. A connection of the nuchal neurite bundle and the drcc is common in other families [27], but could only be found in the Exogoninae.

### Stomatogastric nervous system

In annelids with a muscular axial protrusible pharynx ring-shaped neurite bundles are common in the pharynx [27, 39] and references therein). Syllid species with an s-shaped pharynx in resting position were described to possess two ring neurite bundles, whereas species with a straight pharynx have only one [50]. However, all observed species have (at least) two stomatogastric ring neurite bundles. Apart from this, the present results are very similar to early descriptions of *Autolytus longeferiens* (valid as *Epigamia alexandri* [71]) [50], where five pairs of stomatogastric nerves reach the first and second ring nerve, after which two nerves continue, which again split into two. The second pharyngeal ring nerve was described to lie at the position where sheath and pharyngeal tube join [50]. From there nerves reach towards the anterior end of the pharynx to innervate the papillae, while the first pharyngeal ring nerve sits inside the pharyngeal sheath [50]. In contrast, the present study shows that the first pharyngeal ring neurite bundle lies at the junction of sheath and pharyngeal tube and the second one is restricted to the epithelium of the pharyngeal tube.

Four pairs of stomatogastric nerves were described in Syllidae with a straight pharynx [50]. Thus there are either differences among genera in the pharyngeal innervation, or the exact innervation pattern could not be observed with the available methods [50]. Unfortunately, it was not possible to observe the innervation of the anterior pharynx in *Myrianida* due to weak staining of the stomatogastric nerves and strong autofluorescence of the cuticularized parts, which might have shed light on these differences. In other studies on Syllidae, only three pairs of stomatogastric nerves were observed [32].

Few detailed studies are available on the stomatogastric nervous system in annelids. A muscular axial protrusible muscular pharynx is typical for Phyllodocida [72], but the innervation patterns have not been compared so far. In Nereididae five pairs of stomatogastric nerves were found [11, 73] (suppl. Fig. S4b), similar to the results in Syllidae of the present study. Unfortunately the course of the stomatogastric nerves in Nereididae was not further described, however from CLSM-images of *Neanthes arenaeodentata* it seems like stomatogastric nerves 2 and 3 fuse [11], while in Syllidae 3 and 4 fuse, which points to some variation among families even if the number of stomatogastric nerves is the same. Nevertheless there are also several interconnections between 2, 3 and 4 in Syllidae. Further studies on the pharyngeal innervation of Phyllodocida could resolve if initially five stomatogastric neurite bundles are part of the ground pattern in Phyllodocida, and if these neurite bundles continue in a similar manner in all families.

The course of the stomatogastric neurite bundles in *Prospharosyllis marmarae*, first reaching towards the anterior edge of the prostomium and then turning posteriorly, is similar to descriptions of the hesionid *Microphthalmus* [2] where they are additionally connected by a commissure. This may have functional reasons e.g. a specific sensory function of the prostomial edge and is probably an analogy among these species.

### Ventral nerve cord

Three connectives are most common in Syllidae and have been observed in several genera [27, 50, 74, 75]. Moreover, a trineuralian ventral nerve cord has been reported for many annelid species across a variety of families [2, 5, 25, 27, 39, 76–78]. It is present in *Scoloplos armiger* (Orbinniidae), the dinophilids *Dinophilus gyrociliatus, Dinophilus gardineri, Trilbodrils axi, Trilobodrilus heideri,* and *Trilobodrilus hermaphroditus*, *Ctendrilus serratus* (Ctenodrilidae), *Ophelia rathkei* (Opheliidae), *Capitella capitata* (Capitellidae), *Platynereis dumerilii* (Nereididae), the dorvilleids *Ophryotrocha gracilis, Dorvillea bermudensis* and *Parapodrilus psammophilus*, *Histriobedella homari* (Histriobdellidae), *Trochonerilla mobilis* (Nerillidae) (the nerillids *Nerilla antennata*, *Mesonerilla intermedia* and *Nerillidium mediterraneum* have only 1 longitudinal nerve), *Potamodrilus fluviatilis* (Potamodrilidae), *Aeolosoma bengalense* (Aeolosomatidae), as well as in *Myzostoma cirriferum* (Myzostomida) and *Lobatocerebrum riegeri* (Lobatocerebridae). In addition a median nerve is present in *Hesionides arenaria* (Purschke et al. unpubl.obs.) whereas such a nerve could not be demonstrated in the closely related species *Microphthalmus listensis, M. sczelkowii, M. similis* (2, Purschke et al. unpubl. Obs.). However, as mentioned by several authors (*e.g.* [26, 27]) visibility of the median nerve also depends on the method applied or the developmental stage(s) investigated. For instance, Müller [2] described a short median nerve present in the anteriormost part of the ventral cord in *Magelona* sp. whereas this nerve was not mentioned by Beckers et al. [79]. There seems to be no clear phylogenetic distribution of the trineuralian nerve cord as it is found both in different families of Pleistoannelida with variations within families and possibly in the early branching Magelonidae. In *Eurysillis tuberculata*, a genus very similar in morphology to *Plakosyllis* except for its dorsal tubercles [44], five ventral connectives were observed in TEM sections [74]. A recent study analysed the ventral nerve cord of different annelid families and found that a ladder-like construction of the ventral nerve cord is found in a variety of taxa of annelids [29]. The number of connectives may thus be associated with specific morphological and ecological properties of a given species raher than with its phylogenetic position.

### Segmental nerves

Syllidae can have three or four segmental ring nerves and several additional segmental neurite bundles. In a study on the nervous system of Naididae (Clitellata) the number of segmental ring neurite bundles was compared over various families of annelids using ancestral character estimation analysis [20]. It was concluded that four segmental neurite bundles are probably the most ancestral state for annelids, with various events of reduction and newly formed segmental neurite bundles [20]. However the authors did not differentiate between ring neurite bundles and other segmental neurite bundles (e.g. in Nereididae [11, 37]). Syllidae were characterised with three segmental neurite bundles [20], while it has been shown that they can possess between four and up to seven when all segmental neurite bundles are included [27]. Thus it remains difficult to compare segmental neurite bundles among species, especially when different authors report divergent numbers of segmental neurite bundles. In addition, it seems very likely that the fourth segmental ring neurite bundle in syllids is not present in all species within the family. Thus, interspecific variation makes the reconstruction of a plesiomorphic state of segmental neurite bundles for syllids difficult. This is not exceptional, considering different numbers of segmental nerves have beendescribed within certain species of Nereididae [37, 80] (suppl. Fig. S1). Moreover, different authors may count segmental neurite bundles in a different fashion, *e.g.* in the present study the neurite bundles innervating the parapodia were not differentiated into separate segmental neurite bundles, while others [27] counted the anterior and main neurite bundles of Syllidae as separate segmental neurite bundles. Fine segmental neurite bundles not forming ring commissures can arise to innervate various sensory structures, which are species or genus specific (e.g. ventral papillae in the Exogoninae). Comparing results obtained by different methods may likewise cause problems; it has been shown in many other annelids that the FMRF-lir, serotonin-lir and α-tubulin-lir parts of the nervous system, e.g. the segmental neurite bundles, are not totally in accord with each other. Moreover, the existence of fine dorsal commissures may have been overlooked in earlier studies not employing immunocytochemistry, *e.g.* [37] could not confirm whether the segmental neurite bundles I, II, IV meet in the dorsal midline of nereidids.

### Parapodial innervation

Few descriptions are so far available for the complete innervation of annelid parapodia. The parapodial innervation in Syllidae comprises three to five neurite bundles, while in other annelids *e.g. *nereidids, the parapodial innervation usually consists of only one neurite bundle leaving the ventral nerve cord [2, 11, 37, 80, 81]. It is possible that immunocytochemical stainings do not reveal all elements of the nervous system, and neurite bundles appear as separate, even though they comprise one single neurite bundle. In semi-thin sections separate parapodial neurite bundles could not be distinguished. Likewise, not all of the remaining segmental neurite bundles could be found either on this level and the lack of resolution prevents the differentiation between small neurite bundles. Another possibility is that some of the neurite bundles represent motorneurons while others are neurites of receptor cells and therefore separated into distinct bundles. But as all parapodial neurite bundles are interconnected, they very likely consist both of efferent and affarant fibres, with the majority being afferent [81–84]. So far only species with mixed segmental neurite bundles consisting of moto- and sensory neurons were reported [85]. The reason for several distinct neurite bundles to form the parapodial innervation in Syllidae remains speculative. As the pattern differs among species it may be related to different functions and movability of the parapodia.

While the fine neurite bundles of the parapodial innervation are difficult to homologise at present, at least the main neurite bundle, which splits into two roots, can be homologised [2, 25]. An anterior bundle is present in *Microphthalmus listensis*, *Microphthalmus sczelkowii* (Hesionidae), *Ophelia rathkei* (Opheliidae), *Ophyrotrocha gracilis* (Dorvilleidae) [2], but its course was not described in detail. Dorsal parapodial connectives are present in *Glycera*, too [2]. Further studies in more errant families could facilitate the homologisation of the parapodial innervation and, thus, shed light on possible interfamily variation and on the evolution of parapodia in annelids.

Variation of the parapodial innervation has been reported within nereidids: some species have an anterior neurite bundle that branches off from the nerve innervating the ventral cirrus and forms a dorsal arch along the parapod [37], which is missing in other species [11, 80]. The most posterior neurite bundle innervating the ventral side of the parapodium is also not found in all Nereididae [11, 37, 80]. Such intrafamily variation could not be observed in the five detailed analysed species of Syllidae.

Neurite bundles innervating the chaetal sac were described from serotonin-lir signal in *Sabellaria* [7, 86] and a neurite bundle associated with the acicular chaetal sac was shown in TEM images of *Microphthalmus carolinensis* [86], but it is not mentioned where the neurite bundle ends. Tubulin-lir neurite bundles envelope the chaetae in Syllidae. A serotonin-lir signal reaches close to the chaetal muscle fibres. These signals are likely both bristle receptors (tubulin-lir signal, signal is relatively weak) and motoric fibres (serotonin-lir) as described for *Alitta* (*Nereis/Neanthes) virens*, *Hediste* (*Nereis) diversicolor* [81], *Harmothoe* [84] and *Pisione remota*, *Glycera alba* and *Ophryotrocha gracilis* [2], respectively. The acicular neurite bundle terminates at the acicular muscle and very likely is motoric. The bristle innervation is very conspicuous in serotonin-lir, but very faint in α-tubulin-lir which is probably why they were not found in the α-tubulin-lir study of *Neanthes arenaceodentata* [11]. The parapodial innervation in Syllidae is generally very similar to the situation in Nereididae, both when comparing descriptions of *Alitta (Nereis/Neanthes) virens* and *Neanthes arenaceodentata* where the nerves split into four [11, 80] or five [37] neurite bundles.

### Peripheral interconnections of the segmental innervation

The interconnection between the parapodial neurite bundle and segmental ring neurite bundle III in *Syllis garciai* is similar to the interconnection of segmental neurite bundle II (parapodial neurite bundle) and IV in Nereididae [11, 37], but a homologisation is precarious at present. Segmental neurite bundle IV in Nereididae is similar to segmental ring neurite bundle III in Syllidae, but it is not clear if it forms a dorsal commissure in Nereididae [37]. The segmental neurite bundle between segmental ring neurite bundle II and III is present in both *Syllis* species. In *Syllis tyrrhena* it joins the ventral longitudinal neurite bundle and is very similar to segmental neurite bundle III of *Hediste (Nereis) diversicolor, Alitta (Nereis/Neanthes) virens*, *Platynereis dumerilii* and *Neanthes arenaceodentata* [11, 37], but again homologisation is uncertain. It is not present in any other investigated syllid species and, while it may be homologous to neurite bundle III in the Nereididae, it may as well be an analogous structure. The peripheral innervation is highly variable between species and may be species specific.

### Longitudinal neurite bundles

Peripheral longitudinal neurite bundles are common in many annelid taxa [25]. At least in the five species of Syllidae studied in detail here and *Streptosyllis websteri*, the number and origin of longitudinal segmental neurite bundles is consistent across species. However, the longitudinal neurite bundles can easily be overlooked due to their discontinuous course and small size. To evaluate whether these neurite bundles are homologous to longitudinal neurite bundles in other annelid taxa, it would be helpful to compare their origin in the anterior segments.

While dorsal, lateral, ventrolateral and more additional longitudinal neurite bundles were found in many species, their origin could not be traced in most of them [2]. In *Polygordius appendiculatus* the dorsolateral longitudinal neurite bundles originate in part directly from the brain, from nervous fibres around the nuchal organs, and from fibres deriving from the nuchal neurite bundle. The lateral neurite bundles either originate from the first segmental ring neurite bundle or from the brain [14]. In *Scoloplos armiger* the situation is similar; a pair of dorsolateral neurite bundles originates directly from the brain together with the innermost nuchal nerve and the paired lateral neurite bundle branches off from the circumoesophageal connective just behind the junction of its dorsal and ventral roots [87]. A pair of small neurite bundles that probably form the dorsolateral longitudinal neurite bundles originates directly from the brain and passes the nuchal neurite bundles in *Glycera alba*, but a direct connection to the nuchal neurite bundles could not be traced [2]. The lateral longitudinal neurite bundles were described to originate from the brain just lateral to the dorsal root of the circumoesopageal connective in Amphinomidae, Euprosynidae, Nereididae and some other “polychaetes” [39]. The origin of lateral neurite bundles inLumbriculidae (Clitellata) is unclear [39]. In *Enchytraeus* only its origin from the segmental nerves was described [1].

In the *Syllis* species the longitudinal neurite bundles do not originate from the brain but from a neurite bundle branching off the circumoesophageal connective. The dorsal longitudinal neurite bundle also receives fibres from the nuchal organ (efferent fibres of the nuchal organ, see [27]) and innervates the dorsal ciliary organs, as in *Polygordius appendiculatus*. In other “polychaetes” it was suggested that the nuchal neurite bundle innervates the so called dorsal ciliary organs [64, 88, 89]. Dorsal ciliary bands or patches of cilia are found in many annelid families [27]. They can be chemosensory and in this case are serially repeated nuchal organs [88, 89], create water currents [70] or are involved in reproduction [90]. The function of these ciliary organs in Syllidae is at present unknown, but they are common in many species and are a character of taxonomic value [91]. Fibres originating from the nuchal commissure form the nuchal neurite bundle, and the neurite bundle innervates the dorsal organs in many annelids [25]. Such a commissure could not be found in Syllidae, but may be difficult to locate in immunocytochemical stainings [2].

As the origin of the dorsal and lateral longitudinal neurite bundles in Syllidae differ from descriptions in other species, a homology is uncertain. However, at least in some cases, the dorsal longitudinal neurite bundles are clearly associated with the nuchal organ [27], so either the dorsal longitudinal neurite bundles are convergent structures, or their origin from the circumoesophageal connectives has been overlooked in many species. There is little information available on other Phyllodocida except *Nereis* [37, 81] and *Neanthes arenaceodentata* [11]*.* The description of the three-chaetigerous stages of *N. arenaceodentata* [11] resembles the situation in the Syllidae: the dorsolateral longitudinal neurite bundle joins the dorsal root of the circumoesophageal connective and connects it to the peripheral segmental neurite bundles.

The available data are not detailed enough to compare the origins of longitudinal neurite bundles in Phyllodocida and Aphroditiformia, but with additional information on more taxa, a homology assessment at the suborder level seems feasible.

### Pygidium

A true pygidial ring neurite bundle, as described in *Platynereis dumerilii* [18] was not observed. A pygidial ring neurite bundle was also described in *Typosyllis antoni* [75], but a recent comparison of a large number of Phyllodocida found such a closed ring neurite bundle only in Nereididae [92]. In previous descriptions of Syllidae, neurite bundles reach dorsally but do not connect, similar to the observations made in this study. The anal papilla of *Typosyllis antoni* and *Syllis fasciata* receive their innervation from the pygidial commissure [92], which is probably also the case for other Syllidae, but was not observed in this study.

## Conclusions

The results of this study show a high variability in nervous system architecture among species of different subfamilies of the Syllidae. In the anterior nervous system, the occurrence of clusters of somata in the brain, the presence, morphology and number of laterofrontal sense organs, the numer of palp nerves (possibly not always possible to observe), the presence of dorsal lobes, the morphology of the nuchal lobes and the position of the somata of primary sensory cells of the nuchal organ (only *Plakosyllis*) is variable between species (suppl. Fig. S1). In the segmental innervation, differences in the numbers of connectives in the ventral nerve cord and the number of segmental neurite bundles were found.

The general structure of the brain with its posterior neurite bundles, the trineuralian ventral nerve cord, the stomatogastric nervous system consisting of initially five neurite bundles and two stomatogastric ring neurite bundles, a pair of ventral, lateral and dorsal longitudinal neurite bundles in anterior segments, at least three segmental ring neurite bundles and the innervation of the parapodia are possible conserved structures present in all Syllidae.

The presence of dorsal-posterior lobes in both Exogoninae very likely correlates with miniaturization and morphological adaptations to interstitial habitats, such as a reduced body size and a retractable prostomium in combination with retainment of a a well-developed sensory system. Unfortunately, the histological structure of these lobes could only be observed in the five more thoroughly investigated species (Exogoninae and Syllinae) (see suppl. Fig. S1).

The application of immunocytochemistry and CLSM imaging allows visualising even very fine neurite bundles, which are impossible to observe in histological sections [32]. Thus, previously undescribed neurite bundles innervating the palps were found in Syllidae. The same is true for the innervation of the stomatogastric nervous system, which is a first step to homologising the innervation of the anterior digestive system of Phyllodocida and Aphroditiformia.

The significance of five connectives in the ventral nerve cord of *Plakosyllis brevipes* remains unresolved, but the nervous system architecture correlates with the dorsoventrally flattened appearance of the species.

The presence of three segmental ring neurite bundles is constant in all syllid subfamilies, but a fourth segmental ring neurite bundle was found in several species and the number of additional fine segmental neurite bundles differs among species. As noted previously, segmental neurite bundles may be highly variable among species of a given family [27], and usually correlate with sensory structures such as papillae or epidermal ciliary receptors, which are often species-specific. Nervous system architecture within a genus is fairly similar, as shown for the genus *Syllis,* while there may be great differences among genera.

Another step is taken to permit the homologisation of other nervous structures such as the parapodial innervation, the poorly known innervation of the digestive tract and the segmental ring neurite bundles. The in-depth descriptions of the parapodial innervation extends our knowledge beyond the Nereididae [11, 37, 80, 81] and brings us closer to homologising the fine innervation of parapodia in errant annelids. At least a comparison of the parapodial innervation between Nereididae and Syllidae is feasible and further work on more species will complement the results. The present work further adds to the work on Nereididae by detailed descriptions of the chaetal innervation. The comparison of the innervation of the parapodium in stolon and stock gives insight into the modifications the uniramous parapodium of syllids has undergone compared to a biramous parapodium.

Currently, too little data is available to make assumptions on the implications of nervous system architecture for phylogenetic analysis in Phyllodocida and Aphroditiformia. However, together with the available work on Nereididae, this study gives a first insight into variations and similarities in nervous system architecture of some phyllodocid taxa, such as similarities in parapodial innervation and number of stomatogastric neurite bundles. The nervous system within families seems to mirror the high variability observed between nervous system morphologies between families reported by [29]. This study provides the first detailed describtion of nervous system variation within one family of errant annelids and future studies on more families will allow reconstructing a ground pattern for higher taxa within Annelida or rather the correlation of nervous system architecture with different ecological lifestyles.

## Methods

### Aims and design

Specimens of different species from four subfamilies of Syllidae were collected to reconstruct the nervous system using immunocytochemistry, confocal laser scanning microscopy (CLSM) and histological semi-thin sections. Additionally, maximum intensity z-projections of CLSM scans of the species *Syllides longicirra*, (Anoplosyllinae), *Myrianida prolifera* (Autolytinae), *Eusyllis blomstrandi*, *Nudisyllis* c.f. *pulligera* (“Eusyllinae”), *Brania clavata*, *Brania pulsilla*, *Parapionosyllis labronica*, *Parapionosyllis minuta*, *Sphaerosyllis hystrix*, *Sphaerosyllis tetralix* (Exogoninae). *Syllis krohnii* and *Syllis* sp*.* (Syllinae) were provided from unpublished data of former studies (Kuper, Wieland, Köhler, Purschke, Osnabrück, Germany) (see Table 1), sighted and included in the results where applicable. Staining protocols applied for these species were similar to the ones described below.

### Species collection, fixation and species identification

Specimens of the species *Syllis garciai, Syllis tyrrhena, Plakosyllis brevipes*, *Prosphaerosyllis marmarae* and *Streptosyllis sp*. (juvenile) were collected from the Adriatic Sea in Croatia near Rovinj. Specimens were collected from two locations: Veštar at 2-3 m depth and Punta Croce at 6-8 m depth. Specimens of *Sphaerosyllis taylori* are from the Levantine Sea and were collected in Lebanon at a public beach in Byblos at 2-3 m depth. *Streptosyllis websteri* and *Exogone naidina* were collected in List, Sylt, Germany. *Myrianida* sp. was collected at the marine biological station Espegrend of the Department of Biology at the University of Bergen from hydrozoan colonies. Other species collected for earlier studies were sampled from sediments, but several are known to be found in other habitats too and not all of them belong to the interstitial meiofauna. Sampling location and species used in the study are summarized in Table 1.

Sand was collected in buckets from the sea bottom by snorkelling. After resting over night or longer a few spoons of sand were immersed in seawater isotonic MgCl_2_ in a flask, stirred and left to rest for about 10 min. Subsequently the flask was shaken to detach animals from sand grains, and MgCl_2_ solution was sieved through nets with a mesh size of 250 μm or 125 μm. The sieves were washed out with seawater to collect the animals. Animals were identified to family level under a stereomicroscope, again anesthetised in MgCl_2_ solution and fixed in 4% paraformaldehyde in phosphate buffer (PB) for 1–2 h at room temperature or overnight in the fridge. Best results were obtained with specimens fixed for no more than 1.5 h. Afterwards specimens were washed 3–4 times in phosphate buffer and stored in PB with 0.1% NaN_3_. Animals were identified to species level with a light-microscope before staining. Selected specimens were photographed with a Nikon Eclipse E800 light-microscope (Nikon, Chiyoda, Tokyo, Kapan) and a Nikon DsFi2-U3 microscope camera. Several books and papers were used for species identification [44, 59, 93–96].

### Immunocytochemistry and confocal laser scanning microscopy

Specimens of the species *Syllis garciai* and some specimens of the other species were treated for 30–60 s in an ultrasonic bath to permeabilise the tissue prior to staining. Very good staining results could be obtained for *S. garciai* after freezing some of the specimens in PB with 0.1% NaN_3_. Unspecific binding sites were then blocked by incubation of specimens overnight in 6% normal goat serum (NGS) (Invitrogen, Massachusetts, USA) in PBS with 2–10% Triton X-100 (PBT). Specimens were then incubated at room temperature or at 35° in primary antibodies directed against serotonin (5-HT, raised in rabbit) (Immunostar, Hudson, WI, USA and Sigma-Aldrich, Vienna, Austria) and acetylated α-tubulin (raised in mouse) (Sigma-Aldrich) in a concentration of 1:100–1:2000 in PBT overnight. Specimens were washed 5–6 times in phosphate buffer for 30–60 min and incubated over night or for up to two nights with secondary antibodies Alexa Fluor 568 (goat anti rabbit) (Invitrogen and Dianova Gmbh, Hamburg, Germany) and Alex Fluor 633 (goat anti mouse) (Invitrogen and Jackson Immuno-Research, Soham, UK) at a concentration of 1:200–1:400 in PBT. Additionally, DAPI (4′,6-diamidino-2-phenylindole) (Invitrogen, Carlsbad, CA, USA) and Alexa Fluor 488 phalloidin (Molecular Probes, Eugene, OR, USA) with a dilution of about 1:120 were added. Best results were obtained with a dilution of 1:800 or 1:1000 for primary antibodies against α-tubulin. Antibodies against serotonin were used in dilutions of 1:400, 1:800 or 1:1000 without major differences in the results, but less background staining with higher dilutions. Secondary antibodies were diluted 1:300.

Concentrations of 4% or 10% Triton-X in PBS both lead to good staining results. An increased incubation temperature (35 °C) of primary and secondary antibodies significantly improved serotonin labelling. Generally, results differed among specimens and species even when using the same methods. Some specimens were incubated with one of the antibodies directed against serotonin or α-tubulin and DAPI only, with and without phalloidin. Generally, no improvement in results could be observed when omitting one of the antibodies or phalloidin labelling. A couple of specimens were incubated with primary antibodies directed against serotonin of a different company (Sigma-Aldrich, Vienna, Austria) and results were similar. A few specimens were stained with FMRF antibodies (Immunostar), but the antibodies did not penetrate the tissue well. Again results could be improved when using elevated incubation temperatures, but were still not ideal and are therefore not included in the results.

Samples were scanned using a Leica TCS SP5 II confocal laser scanning microscope (Leica Microsystems, Wetzlar, Germany).

### Histology

Specimens were either fixed in 4% PFA for 2 h or in 2% glutaraldehyde for several days. Most specimens were postfixed in osmium tetroxide (OsO_4_). Fixed specimens were washed three times in PBS and dehydrated in acidic 2, 2-dimethoxypropane (DMP). Before infiltration in agar low-viscosity resin (LVR, Agar Scientific, Stansted, UK), specimens were washed 3x in 100% acetone. Resin blocks were polymerised at 60°. Ribbons of 0.5-1 μm serial semi-thin-sections were cut with a Histo Jumbo diamond-knife (Diatome, Biel, Switzerland) on a UC6 Ultramicrotome (Leica Microsystems, Wetzlar, Germany) following the protocol of [97]. Serial sections were dyed in toluidine blue and embedded in LVR. Serial sections were photographed using a Nikon Eclipse E800 light-microscope and a Nikon DsFi2-U3 microscope camera.

### Image processing

Photoshop CS6 (Adobe Systems, San Jose, USA) was used to stitch several images of the same specimen together *e.g.* of longitudinal semi-thin-sections using the photomerge function. Z-projections of confocal scans were obtained in Fiji [98]. Further image processing and annotations were done in GIMP (GNU Image Manipulation Program, Version 2.8.14, GNU general public licence), Scribus (Version 1.4.6, GNU general public licence, using Ghostscript version 9.19) and Inkscape (Version 0.91, GNU general public licence). Illustrations were created in Inkscape. Photos of serial semi-thin sections were converted to 8bit greyscale and reduced in size in Adobe Photoshop CS6 or Fiji and subsequently loaded into Amira (Version 5.4 and 5.5, FEI Visualization Sciences Group, Mérignac, France) and aligned using the alignment editor. Results of the automatic alignment were adjusted by hand. Afterwards regions of interest were segmented to produce selected volume renderings or 3D surface models, which were used as reference for the included illustrations.

## Supplementary information

**Additional file 1 Figure S1**. Schematic phylogenetic tree of Syllidae, species for which data were obtained, anterior and segmental innervation in each subfamily. Asterisks indicate a character observed in at least one, but not all of the species in a subfamily. Fine innervation patterns only observed in some of the species are omitted. Details on the distribution of somata of the nervous system and of the nuchal organ can only be given for Exogoninae and Syllinae, as histological data is missing in the other subfamilies. Information about Nereididae combined from [10, 11, 25, 37, 72, 79]. Other families of the Phyllodocida were omitted for better readability. Abbreviations: mn – main nerve, mvn – median ventral nerve, pmn – paramedian nerve, I - IV – segmental neurite bundles.

**Additional file 2 Figure S2.***Streptosyllis websteri*. Innervation of head, pharynx, segments and pygidium. Maximum intensity z-projections of α-tubulin-lir (grey). **A**: Detail of the brain showing the nuchal organ. The white arrowhead indicates neurite bundles leading from the nuchal organ towards the posterior neurite bundles of the brain. Orange arrowhead indicates a ciliary patch behind the nuchal organ. **B**: Innervation of the pharynx. **C**: Ring neurite bundles forming dorsal commissures and segmental ciliary bands. **D**: Ventral nerve cord and segmental neurite bundles. *S. websteri* does not have a forth, intersegmental ring neurite bundle **E**: Parapodial innervation. Neurites bundles reaching from the ventral nerve cord to the parapodium appear either as two or three separate bundles, depending on the scan. **E**: Pygidial innervation. **Scale bars = 50 μm. Abbreviations:** br – brain; cno – cilia of support cells of nuchal organ; cc – circumoesophageal connective; cr – ciliary receptors; in – intestine; lan – neurite bundle innervating lateral antenna; lfs – laterofrongal sense organ (homology unclear); man – neurite bundles innervating median antenna; mn – main ventral nerve; mvn – median ventral nerve; pcm – pygidial commissure; pln – neurite bundles innervating parapodial lobe; pon – posterior neurite bundles of the brain; ppa – pharyngeal papilla; r1 – stomatogastric ring neurite bundle 1; r2 – stomatogastric ring neurite bundle 2; rp1 - first root of main parapodial neurite bundle; rp2 - second root of main parapodial neurite bundle; stgn – stomatogastric neurite bundle; **Segmental neurite bundles in yellow**: I-III – segmental neurite bundles forming ring commissures. II.I-II.III – neurite bundles innervating parapodium.

**Additional file 3 Figure S3**. *Myrianida sp.* Innervation of head, nuchal eupalettes and segments*.* Maximum intensity z-projections of α-tubulin-lir (grey). **A**: Prostomium and anterior segments of *Myrianida sp*. The pharynx shows a strong autofluorescent signal. The nuchal eupalettes are marked in light blue. **B**: Detail of the brain of *Myrianida sp*. The posterior neurite bundles of the brain reach directly into the nuchal eupalettes, connecting to the primary sensory cells. **C**: Segmental innervation of *Myrianida prolifera*. The fourth segmental neurite bundle is missing in irregular patterns (red arrows). **Scale bars = 50 μm** (missing for C). **Abbreviations**: br – brain; cr – ciliary receptors; dc – dorsal cirrus; lan – neurite bundle innervating lateral antenna; lfs – laterofrongal sense organ (homology unclear); man – neurite bundles innervating median antenna; mn – main ventral nerve; mvn – median ventral nerve; ph – pharynx; pon – posterior neurite bundles of the brain. **Segmental neurite bundles in yellow**: I-IIV– segmental neurite bundles forming ring commissures. Micrograph C Courtesy of Dr. M. Kuper.

**Additional file 4 Figure S4**. Anterior nervous system of Syllidae and Nereididae as described previously. The neuropil of the brain is connected to the ventral nerve cord via the circumoesophageal connective. Both the dorsal and the ventral root of the circumoesophageal connective form a dorsal and a ventral commissure within the brain. The dorsal commissure of the dorsal root of the circumoesophageal connective (dcdr) forms nerve tracts that reach toward the posterior ganglia, which are involved in the innervation of the nuchal organ. Smaller commissures were omitted. **A**: Syllidae redrawn after [32] and [25]. The dcdr sends fibres to the lateral and median antennae. A pair of dorsal ganglia lies above each dorsal root of the circumoesophageal connection. It is innervated by fibres originating from the ventral side of the neuropil of the brain. A cluster of globuli cells lies at the beginning of each palp. The palps are innervated by root 6 coming from the ventral commissure the ventral root of the circumoesophageal connective and root 9 coming from the dorsal root of the circumoesophageal connective. Two pairs of stomatogastric nerves emanate from the ventral commissure of the ventral root of the circumoesophageal connective and one from the circumoesophageal connective where dorsal and ventral root fuse. **B**: Nereididae redrawn after [10, 11, 25, 72]. The dcdr and the ventral commissure of the dorsal root of the circumoesophageal connective (vcdr) send fibres to the lateral antennae. At least one pair of mushroom bodies (depending on species, rose, dotted line) lies above the neuropil of the brain. Behind them, at the latteral frontal margins of the prostomium lies the Langdons organ (dotted lines). Seven nerves innervate the palps. Five pairs of stomatogastric nerves innervate the pharynx. Abbreviations: cc – circumoesophageal connective; dcdr – dorsal commissure of drcc; dcs – dorsal cluster of somata (dorsal ganglion); dcvr – dorsal commissure of vrcc; drcc – dorsal root of the circumoesophageal connective; gl – ganglion of globuli-like cells; lan – lateral antenna; man – median antenna; nn – nuchal nerve; pgl – posterior ganglion; pn1–12 – palp nerves; stgn 1–3 – stomatogastric nerves 1–3; tcn – neurite bundle innervating tentacular cirri; vcdr – ventral commissure of drcc; vcvr – ventral commissure of vrcc; vrcc – ventral root of the circumoesophageal connective.

## Data Availability

The original datasets and analysed data are available upon request from the corresponding author.

## Abbreviations

1: First fine segmental neurite bundle
2: Second fine segmental neurite bundle
6: Fibres possibly homologous to palp neurite bundle root 6
9: Fibres possibly homologous to palp neurite bundle root 9
ac: acicle
acdr: anterior commissure of dorsal root
acm: acicular muscle
acn: neurite bundle accompanying acicle
acvr: anterior dorsal commissure of vrcc
ae: anterior eye
aen: neurite bundle innervating anterior eye
alc: a-tubulin-lir anterior commissure within the anterior ganglion of the stolon
an: antenna
ann: neurite bundle innervating antenna
anu: anus
ap: anterior parapodial neurite bundle
bc: buccal cavity
bl: extracellular matrix
bm: muscle penetrating the brain
br: brain
bv: blood vessel
cae: caecum
cc: circumoesophageal connective
ccp: cells of crescent shaped ciliary patch on prostomium of *Syllis* species
ch: chaetae
chm: chaetal muscles
cin: cilia of the intestine
cm: cirrus muscle
cn: neurite bundle accompanying chaetae
cno: cilia of support cells of nuchal organ
cp: cuticularised layer of the pharynx
cpl: cuticularised plates inside proventricle of *P. brevipes*
cr: ciliary receptor
cso: clusters of somata
cu: cuticle
cun: modified cuticle of nuchal organ
cvr: commissure of vrcc
dc: dorsal cirrus
dcb: dorsal ciliary band
dcdr: dorsal commissure of the drcc
dci: inclusions of dorsal cirri in *S. garciai*
dcn: neurite bundle innervating dorsal cirrus
dcs: dorsal cluster of somata
dct: dorsal anterior commissure of tentacular segment
dcvr: dorsal commissure of vrcc
dep: dorsal epidermal plexus
dl: dorsal lobe
dlm: dorsal longitudinal muscle
dln: dorsal longitudinal neurite bundle
dlp: distal innervation of parapodial lobe
dmc: dorsal median a-tubulin-lir commissure in brain of stolon of *S. tyrrhena*
dpc: neurite bundles forming distal parapodial connecitve
dps: dorsoposterior clusters of somata of the vnc
drcc: dorsal root of circumoesophageal connective
dvm: dorsoventral muscles
e: eye
em: embryo
ep: epidermal layer of pharynx
es: eye spot
glp: pharyngeal glands
I-IV: segmental neurite bundles forming ring commissures
Ilr: dorsal commissure of root 2 of II
inm: muscle fibers of the intestine
int: intestine
Isrl: small root of segmental neurite bundle I
la: lateral antenna
lan: neurite bundle innervating lateral antenna
lfs: laterofrontal sense organ
llm: lateral longitudinal muscle
lln: lateral longitudinal neurite bundle
ma: median antenna
man: neurite bundles of median antenna
me: median eye
mcvr: median dorsal commissure of vrcc
mlp: muscular layer of the pharynx
mn: median ventral nerve
mp: main parapodial neurite bundle
mpa: muscle of pharyngeal papilla
mpc: median dorsal parapodial commissure
mvn: main ventral nerve
n: nephridium
nc: notochaetae
ncn: a-tubulin-lir neurite bundle innervating notochaetae
ndln: fibers from nuchal organ joining dorsal longitudinal neurite bundle
nl: nuchal lobe
nn: nuchal neurite bundle
no: nuchal organ
np: nervous plexus
nsc: serotonin-lir signal accompanying capillary notochaetae
nsp: neurite bundle innervating sensory papilla
oc: oocyte
olc: olfactory chamber
pa: palp
pam: palp muscle
pc: parapodial cluster of somata
pcb: posterior commissure of the brain
pcm: pygidial commissure
pcn: neurite bundle innervating pygidial cirrus
pcs: ciliary sense organs on palps
pe: posterior eye
pen: neurite bundle innervating posterior eye
pg: parapodial gland
pgl: posterior ganglion
pgr: pigment granules of the eye
ph: pharynx
pin: nervous plexus innervating intestine
pk: serotonin-lir perikarya
plg: parapodial ganglion/cluster of somata
pln: neurite bundles innervating parapodial lobe
pm: parapodial muscle
pmn: paramedian nerve
pn: palp neurite bundle
pn1: main palp neurite bundle
pn2: palp neurite bundle from drcc
pn3: palp neurite bundle from vrcc
pon: posterior neurite bundle of the brain
ppa: pharyngeal papilla
ppl: parapodial lobe
ppl1: parapodial lobe of first chaetiger
prc: proximal dorsal parapodial commissure
prn: neurite bundles innervating prostomium
psh: pharyngeal sheath
pv: proventricle
pvn: paramedian ventral nerve
pxm: pharynx muscle
py: pygidium
pyc: pygidial cirrus
r1–2: stomatogastric ring neurite bundle 1–2
rp1: first root of main parapodial neurite bundle
rp2: second root of main parapodial neurite bundle
scs: segmental cluster of somata
smf: spherical median front of brain
smp: striated muscles of the proventricle
sn: segmental neurite bundles
sn1,2: segmental neurite bundles of tentacular segment
snp: serotonin-lir nervous plexus of body wall
so: soma
sp.: sensory papilla
spc: spermatocytes
sr: serotonin-lir stomatogastric ring neurite bundle
srl: small root of segmental neurite bundle I in *Pr. marmarae*
srn: segmental ring neurite bundle
stgn 1–5: stomatogastric neurite bundles 1–5
stk: stock
tc: tentacular cirrus
tcn: neurite bundle innervating tentacular cirri
tcnd: neurite bundle innervating dorsal tentacular cirrus
tcnv: neurite bundle innervating ventral tentacular cirrus
th: tooth
tln: a-tubulin-lir fibers entering nuchal lobe
vc: ventral cirrus
vcn: neurite bundle innervating ventral cirrus
ven: ventricle
venn: neurite bundles innervating ventricle
vgl: ventral ganglion (of ventral nerve cord)
vlm: ventral longitudinal muscle
vln: ventral longitudinal neurite bundle
vm: ventral median longitudinal muscle above vnc
vnc: ventral nerve cord
vrcc: ventral root of circumoesophageal connective
