# Supplementary material for: Within-family plasticity of nervous system architecture in Syllidae (Annelida, Errantia)
Source: Front Zool. 2020 Jun 23;17:20. doi: 10.1186/s12983-020-00359-9 (PMC7310387; doi:10.1186/s12983-020-00359-9)
Supplement: Supplementary file 1 — Additional file 1 Figure S1. Schematic phylogenetic tree of Syllidae, species for which data were obtained, anterior and segmental innervation in each subfamily. Asterisks indicate a character observed in at least one, but not all of the species in a subfamily. Fine innervation patterns only observed in some of the species are omitted. Details on the distribution of somata of the nervous system and of the nuchal organ can only be given for Exogoninae and Syllinae, as histological data is missing in the other subfamilies. Information about Nereididae combined from [10, 11, 25, 37, 72, 79]. Other families of the Phyllodocida were omitted for better readability. Abbreviations: mn – main nerve, mvn – median ventral nerve, pmn – paramedian nerve, I - IV – segmental neurite bundles. [file 12983_2020_359_MOESM1_ESM.pdf]

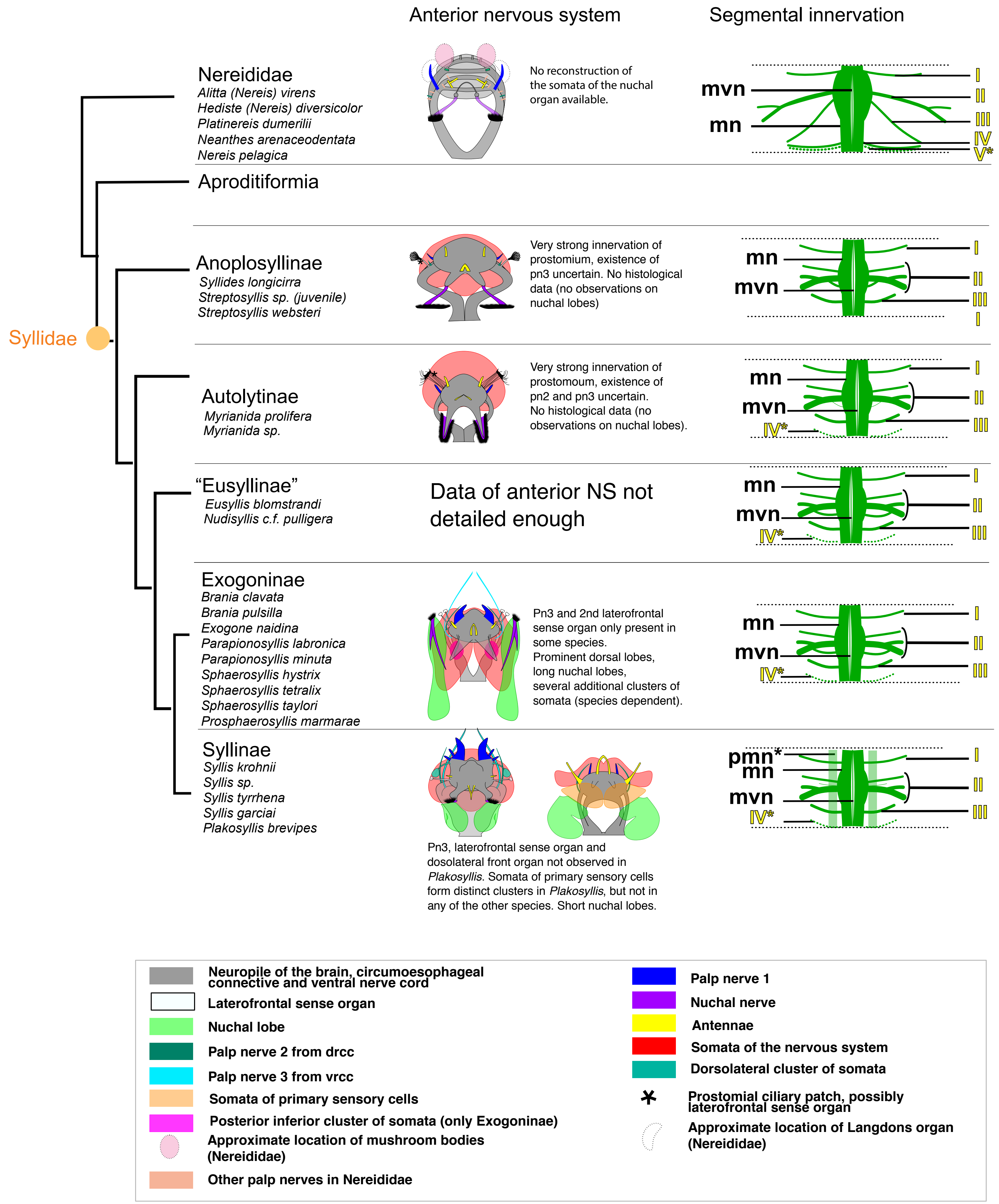
Anterior nervous system
Segmental innervation
Nereididae
Alitta (Nereis) virens
Hediste (Nereis) diversicolor
Platinereis dumerilii
Neanthes arenaceodentata
Nereis pelagica
No reconstruction of the somata of the nuchal organ available.
mvn
mn
I
II
III
IV
V*
Aproditiformia
Syllidae
Anoplosyllinae
Syllides longicirra
Streptosyllis sp. (juvenile)
Streptosyllis websteri
Very strong innervation of prostomium, existence of pn3 uncertain. No histological data (no observations on nuchal lobes)
Autolytinae
Myrianida prolifera
Myrianida sp.
Very strong innervation of prostomoum, existence of pn2 and pn3 uncertain. No histological data (no observations on nuchal lobes).
"Eusyllinae"
Eusyllis blomstrandi
Nudisyllis c.f. pulligera
Data of anterior NS not detailed enough
Exogoninae
Brania clavata
Brania pulsilla
Exogone naidina
Parapionosyllis labronica
Parapionosyllis minuta
Sphaerosyllis hystrix
Sphaerosyllis tetralix
Sphaerosyllis taylori
Prosphaerosyllis marmarae
Pn3 and 2nd laterofrontal sense organ only present in some species. Prominent dorsal lobes, long nuchal lobes, several additional clusters of somata (species dependent).
Syllinae
Syllis krohnii
Syllis sp.
Syllis tyrrhena
Syllis garciai
Plakosyllis brevipes
pmn*
Pn3, laterofrontal sense organ and dosolateral front organ not observed in Plakosyllis. Somata of primary sensory cells form distinct clusters in Plakosyllis, but not in any of the other species. Short nuchal lobes.
Neuropile of the brain, circumoesophageal connective and ventral nerve cord
Laterofrontal sense organ
Nuchal lobe
Palp nerve 2 from drcc
Palp nerve 3 from vrcc
Somata of primary sensory cells
Posterior inferior cluster of somata (only Exogoninae)
Approximate location of mushroom bodies (Nereididae)
Other palp nerves in Nereididae
Palp nerve 1
Nuchal nerve
Antennae
Somata of the nervous system
Dorsolateral cluster of somata
Prostomial ciliary patch, possibly laterofrontal sense organ
Approximate location of Langdons organ (Nereididae)
