# Supplementary figures and images for: Within-family plasticity of nervous system architecture in Syllidae (Annelida, Errantia)
Source: Front Zool. 2020 Jun 23;17:20. doi: 10.1186/s12983-020-00359-9 (PMC7310387; doi:10.1186/s12983-020-00359-9)

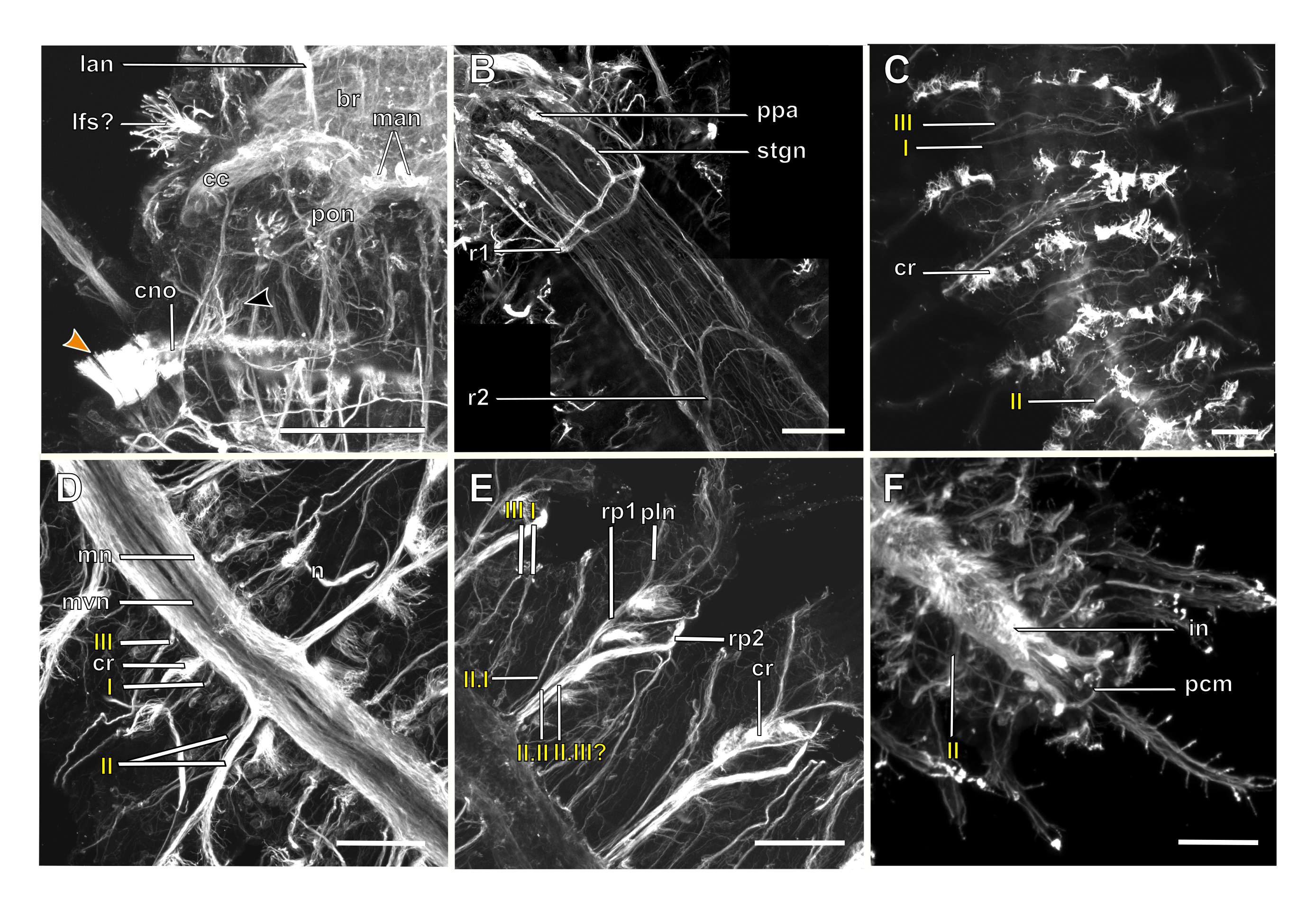

Supplement: Supplementary file 2 — Additional file 2 Figure S2.Streptosyllis websteri. Innervation of head, pharynx, segments and pygidium. Maximum intensity z-projections of α-tubulin-lir (grey). A: Detail of the brain showing the nuchal organ. The white arrowhead indicates neurite bundles leading from the nuchal organ towards the posterior neurite bundles of the brain. Orange arrowhead indicates a ciliary patch behind the nuchal organ. B: Innervation of the pharynx. C: Ring neurite bundles forming dorsal commissures and segmental ciliary bands. D: Ventral nerve cord and segmental neurite bundles. S. websteri does not have a forth, intersegmental ring neurite bundle E: Parapodial innervation. Neurites bundles reaching from the ventral nerve cord to the parapodium appear either as two or three separate bundles, depending on the scan. E: Pygidial innervation. Scale bars = 50 μm. Abbreviations: br – brain; cno – cilia of support cells of nuchal organ; cc – circumoesophageal connective; cr – ciliary receptors; in – intestine; lan – neurite bundle innervating lateral antenna; lfs – laterofrongal sense organ (homology unclear); man – neurite bundles innervating median antenna; mn – main ventral nerve; mvn – median ventral nerve; pcm – pygidial commissure; pln – neurite bundles innervating parapodial lobe; pon – posterior neurite bundles of the brain; ppa – pharyngeal papilla; r1 – stomatogastric ring neurite bundle 1; r2 – stomatogastric ring neurite bundle 2; rp1 - first root of main parapodial neurite bundle; rp2 - second root of main parapodial neurite bundle; stgn – stomatogastric neurite bundle; Segmental neurite bundles in yellow: I-III – segmental neurite bundles forming ring commissures. II.I-II.III – neurite bundles innervating parapodium. [file 12983_2020_359_MOESM2_ESM.png]

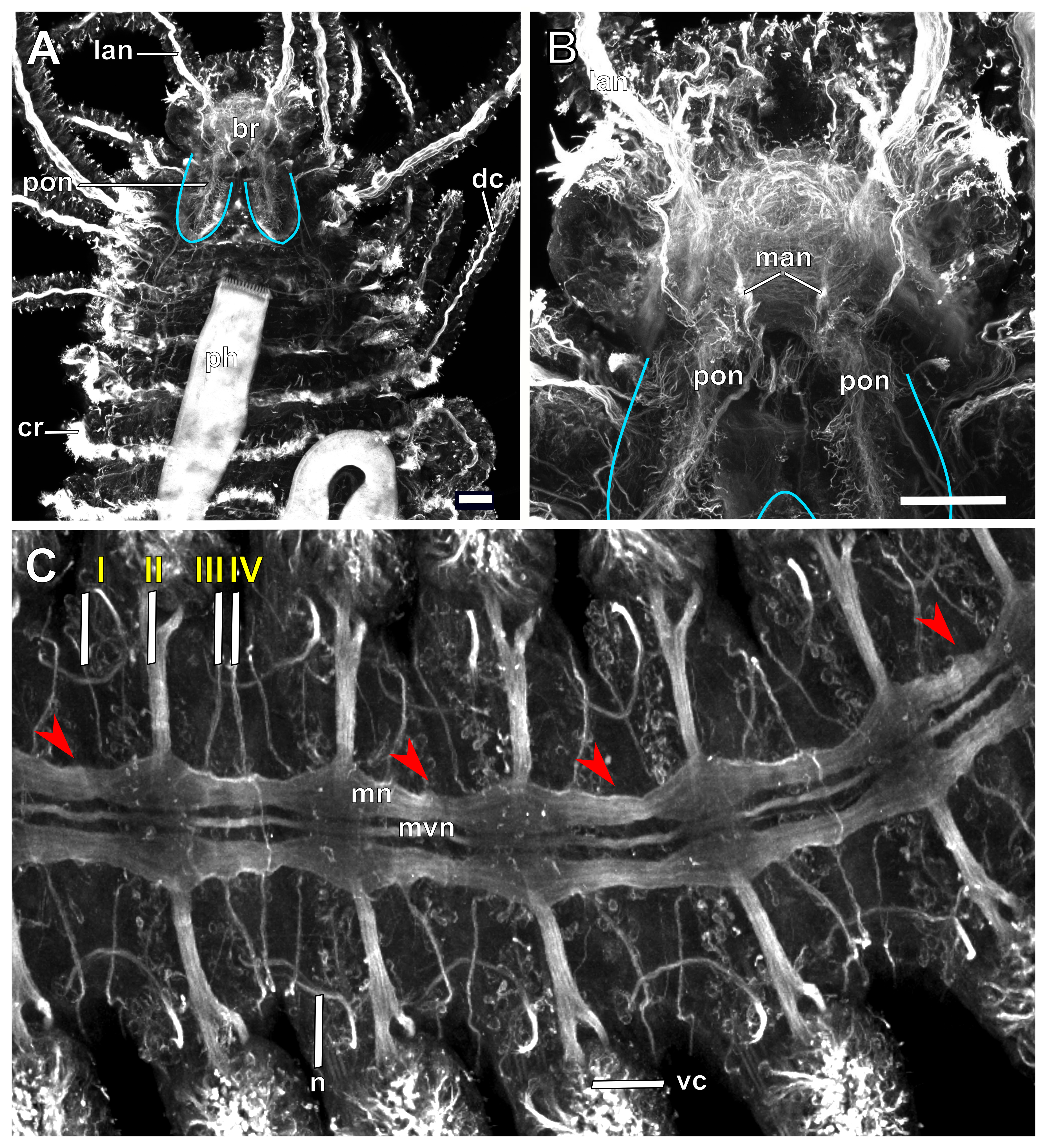

Supplement: Supplementary file 3 — Additional file 3 Figure S3. Myrianida sp. Innervation of head, nuchal eupalettes and segments. Maximum intensity z-projections of α-tubulin-lir (grey). A: Prostomium and anterior segments of Myrianida sp. The pharynx shows a strong autofluorescent signal. The nuchal eupalettes are marked in light blue. B: Detail of the brain of Myrianida sp. The posterior neurite bundles of the brain reach directly into the nuchal eupalettes, connecting to the primary sensory cells. C: Segmental innervation of Myrianida prolifera. The fourth segmental neurite bundle is missing in irregular patterns (red arrows). Scale bars = 50 μm (missing for C). Abbreviations: br – brain; cr – ciliary receptors; dc – dorsal cirrus; lan – neurite bundle innervating lateral antenna; lfs – laterofrongal sense organ (homology unclear); man – neurite bundles innervating median antenna; mn – main ventral nerve; mvn – median ventral nerve; ph – pharynx; pon – posterior neurite bundles of the brain. Segmental neurite bundles in yellow: I-IIV– segmental neurite bundles forming ring commissures. Micrograph C Courtesy of Dr. M. Kuper. [file 12983_2020_359_MOESM3_ESM.png]

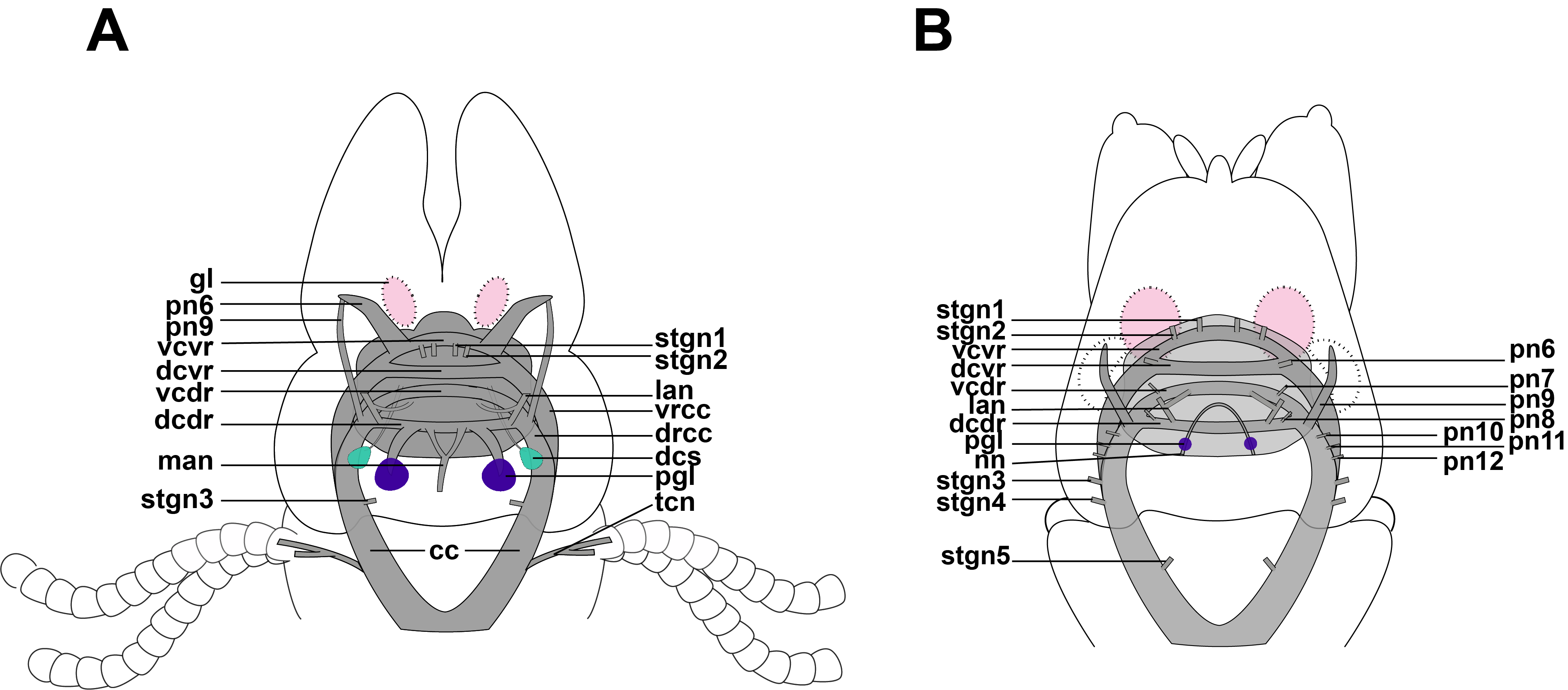

Supplement: Supplementary file 4 — Additional file 4 Figure S4. Anterior nervous system of Syllidae and Nereididae as described previously. The neuropil of the brain is connected to the ventral nerve cord via the circumoesophageal connective. Both the dorsal and the ventral root of the circumoesophageal connective form a dorsal and a ventral commissure within the brain. The dorsal commissure of the dorsal root of the circumoesophageal connective (dcdr) forms nerve tracts that reach toward the posterior ganglia, which are involved in the innervation of the nuchal organ. Smaller commissures were omitted. A: Syllidae redrawn after [32] and [25]. The dcdr sends fibres to the lateral and median antennae. A pair of dorsal ganglia lies above each dorsal root of the circumoesophageal connection. It is innervated by fibres originating from the ventral side of the neuropil of the brain. A cluster of globuli cells lies at the beginning of each palp. The palps are innervated by root 6 coming from the ventral commissure the ventral root of the circumoesophageal connective and root 9 coming from the dorsal root of the circumoesophageal connective. Two pairs of stomatogastric nerves emanate from the ventral commissure of the ventral root of the circumoesophageal connective and one from the circumoesophageal connective where dorsal and ventral root fuse. B: Nereididae redrawn after [10, 11, 25, 72]. The dcdr and the ventral commissure of the dorsal root of the circumoesophageal connective (vcdr) send fibres to the lateral antennae. At least one pair of mushroom bodies (depending on species, rose, dotted line) lies above the neuropil of the brain. Behind them, at the latteral frontal margins of the prostomium lies the Langdons organ (dotted lines). Seven nerves innervate the palps. Five pairs of stomatogastric nerves innervate the pharynx. Abbreviations: cc – circumoesophageal connective; dcdr – dorsal commissure of drcc; dcs – dorsal cluster of somata (dorsal ganglion); dcvr – dorsal commissure of vrcc; drcc – dorsa [file 12983_2020_359_MOESM4_ESM.png]
